# Discovery of a Minimally Charged Cell-Penetrating Peptide

**DOI:** 10.1021/acs.biochem.6c00003

**Published:** 2026-03-11

**Authors:** Saikat Mandal, Jeremy L. Ritchey, Prabhat Bhat, Dehua Pei

**Affiliations:** Department of Chemistry and Biochemistry, 2647 The Ohio State University, 484 West 12th Avenue, Columbus, Ohio 43210, United States

## Abstract

Cell-penetrating peptides (CPPs) are powerful tools for delivering membrane-impermeable biomolecules into eukaryotic cells, with broad applications ranging from therapeutics to biopesticides. However, conventional linear CPPs typically require a high density of positive charges (at least +6) to function, often resulting in dose-limiting toxicity and off-target effects. Reducing this charge without sacrificing delivery efficiency remains a significant challenge. In this study, we performed a structure–activity relationship (SAR) analysis and medicinal chemistry optimization of the bismuth-mediated bicyclic CPP, BCP16. This campaign led to the discovery of BCP16e, a potent analog that carries only a +2 charge at physiological pH. Compared to its parent molecule, BCP16e exhibits significantly higher cytosolic entry efficiency, similar proteolytic stability, and a superior safety profile. Our findings demonstrate that high cationic charge is not a prerequisite for efficient translocation, providing a framework for the design of minimally charged, high-efficiency vehicles for intracellular delivery.

## Graphical abstract

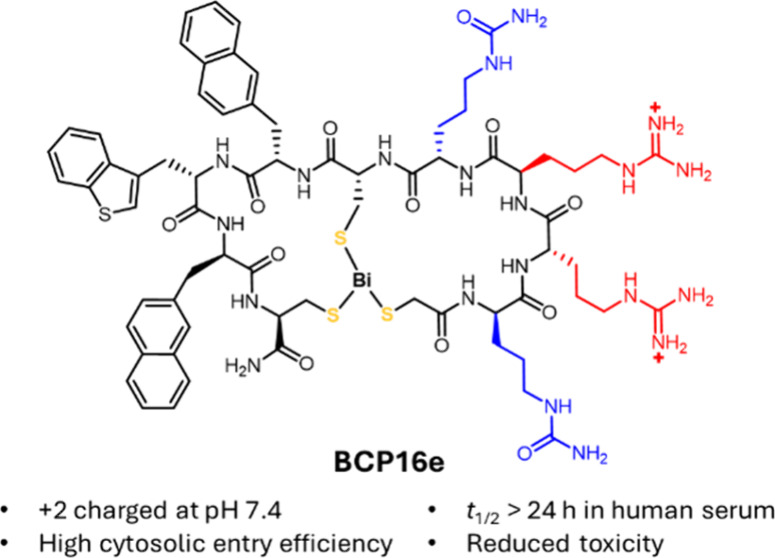

## Introduction

Cell-penetrating peptides (CPPs) are short peptides (typically 5–30 amino acids) capable of autonomously crossing biological membranes without compromising cellular integrity.^1^ Traditionally, CPPs are classified by their chemical composition into cationic (e.g., Tat,^2^ Penetratin,^3^ and R_9_ ^4,5^ ), amphipathic (e.g., MAP,^6^ Transportan,^7^ and L17E^8^), or, less commonly, hydrophobic^9,10^ and anionic families.^11–14^ These peptides facilitate the intracellular delivery of diverse cargoes—including peptides, proteins, nucleic acids, and nanoparticles—via two primary pathways: energy-dependent endocytosis followed by endosomal escape, and energy-independent direct translocation across the plasma membrane.^15^

We recently elucidated that many cationic and amphipathic CPPs utilize a vesicle budding-and-collapse (VBC) mechanism for translocation across the endosomal^16^ or plasma membrane.^17^ In this process, CPPs bind to the membrane to form a CPP-enriched lipid domain that buds into the cytosol as an unstable vesicle. The subsequent spontaneous disintegration (collapse) of this vesicle releases the CPP and its cargo into the cytosol. This topological movement allows large, folded biomolecules to enter the cell without physically traversing the lipid bilayer or disrupting membrane homeostasis.^18^

Despite their versatility in research, the clinical translation of CPPs remains elusive; to date, no CPP-based therapeutic has received FDA or EMA approval. Early linear CPPs were plagued by poor metabolic stability and low cytosolic entry efficiency due to endosomal entrapment.^19–21^ While peptide cyclization has largely overcome these hurdles by enhancing their proteolytic resistance and endosomal escape efficiency,^22–24^ systemic toxicity of polycationic CPPs remains a critical challenge.

Cationic CPPs can induce hemolysis,^25^ pathological Ca^2+^ influx,^26^ and interference with intracellular protein-nucleic acid binding,^27^ among other cytotoxic mechanisms.^28^ In vivo, linear cationic peptides like Tat and R_8_ exhibit significant acute toxicity in rodents, with LD_50_ values as low as 18–27 mg/kg.^29,30^ Crucially, toxicity scales proportionally with the number of arginine or lysine residues—the very same residues required for efficient cellular entry.^31^ This correlation creates a ″charge-activity paradox,″ raising a pivotal question: can we design minimally charged CPPs that maintain high delivery efficiency while minimizing toxicity?

It is generally accepted that linear cationic CPPs require a minimum of six positive charges for significant activity, with R_9_ representing the gold standard.^4,5^ While the inclusion of hydrophobic residues [e.g., Trp, Phe, or 2-naphthylalanine (Nal)] and macrocyclization has reduced this requirement to four Arg residues [as seen in CPP12 (Figure 1)^22^], further reduction has proven difficult.

**Figure 1:**
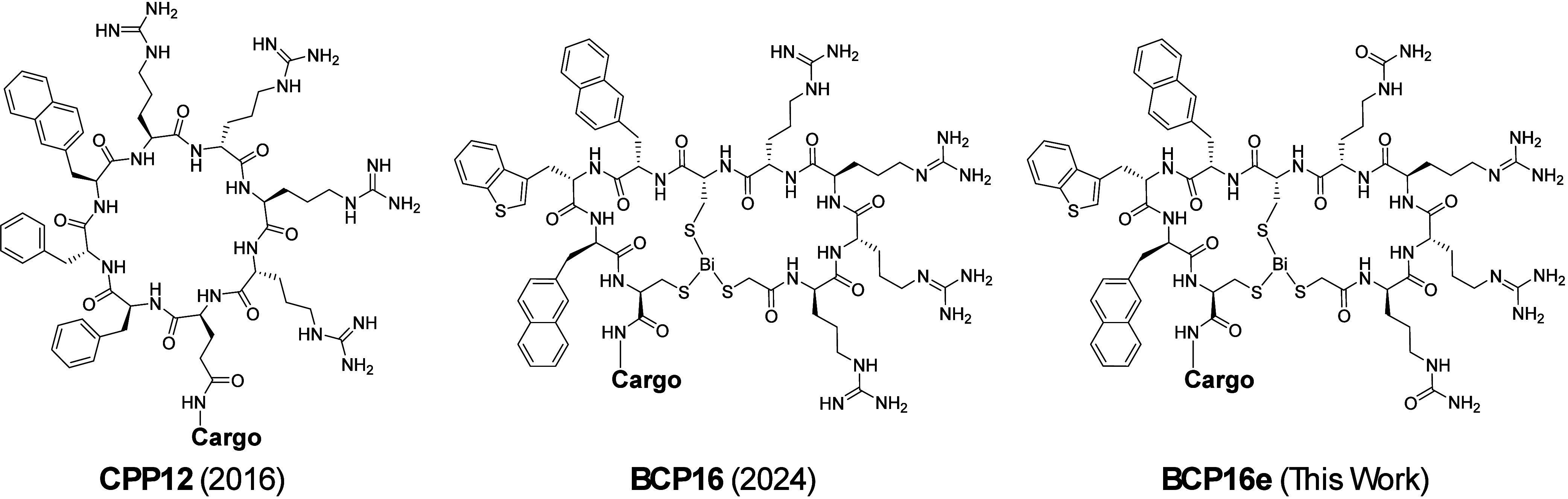
Structures of CPP12, BCP16, and BCP16e.

Our previous work introduced BCP16, a bicyclic CPP formed via the rapid, Bi­(III)-mediated complexation of a thiol-rich precursor (Figure 1).^32^ While BCP16 exhibits excellent cytosolic delivery efficiency and proteolytic stability, its safety profile in HeLa cells and rodents requires further refinement for therapeutic application. Herein, we describe a medicinal chemistry campaign to optimize the BCP16 scaffold. These efforts culminated in the discovery of BCP16e (Figure 1), a variant which carries only two positive charges, exhibits significantly reduced toxicity, but retains high delivery efficiency, demonstrating that high cationic density is not a requirement for potent CPP activity.

## Materials and Methods

### Materials

Fmoc-protected amino acids were purchased from Chem-Impex (Wood Dale, IL), Ambeed (Arlington Heights, IL), or Sigma-Aldrich (St. Louis, MO). DMF, dichloromethane (DCM), piperidine, diisopropylcarbodiimide (DIC), triisopropylsilane (TIPS), and 3,6-dioxa-1,8-octanedithiol (DODT) were purchased from Sigma-Aldrich (St. Louis, MO). Rink amide resin (0.49 mequiv/g, 100–200 mesh) was purchased from Chem-Impex (Wood Dale, IL). Other reagents for microwave-assisted solid-phase peptide synthesis were from CEM (Matthews, NC). LC/MS-grade solvents for HPLC and UPLC, (5(6)-carboxytetramethylrhodamine *N*-hydroxysuccinimidyl ester (TMR-NHS), and naphthofluorescein *N*-hydroxysuccinimidyl ester (NF-NHS) were purchased from Fisher Scientific (Hampton, NH). Carboxy-NF was purchased from Toronto Research Chemicals (Toronto, ON). Cell culture solutions and tissue-treated plates were purchased from Corning (Corning, NY) or Grenier Bio-One (Monroe, NC). OneStep Luciferase assay kit was purchased from BPS Biosciences (San Diego, CA). Human serum (normal pool) was purchased from Fisher Scientific (Hampton, NH). Cell-Titer Glo 2.0 was purchased from Promega (Madison, WI).

### Peptide Synthesis

Peptides were synthesized by using standard solid-phase Fmoc chemistry on a Liberty Blue microwave-assisted peptide synthesizer at 25-μmol scale using Rink amide ProTide resin (0.20 mmol/g, 100–200 mesh, CEM). Coupling reactions were carried out with 5 equiv of Fmoc-amino acid, 10 equiv of DIC, and 5 equiv of Oxyma Pure, typically at 90 °C for 4 min, except for arginine residues which were double-coupled under these conditions. Cysteine residues were introduced at 50 °C for 10 min to minimize epimerization. Following the incorporation of aspartate residues, Fmoc deprotection was performed at room temperature to reduce aspartimide formation. N-Terminal acetylation was carried out on-resin by the treatment (3 times) with 10 equiv of acetic anhydride and 10 equiv of piperidine in DCM for 15 min at room temperature. When required for fluorescent labeling, a miniPEG-lysine motif was appended at the C-terminus. For CPP12 cyclization, after the addition of last (N-terminal) residue, the allyl group on the α-carboxyl group of C-terminal l-Glu residue was removed by treating the resin with 2.5 equiv of Pd­(PPh_3_)_4_ and 15 equiv of phenylsilane in DCM at 35 °C for 5 min (3 times). The resin was then thoroughly washed and the N-terminal Fmoc group was removed by treatment with 20% piperidine in DMF. After washing, the resin was treated (3 times) with DIC/HOBt (10 equiv each) in DMF at 90 °C for 10 min. Peptides were deprotected and cleaved from the resin by treating with a cocktail consisting of TFA/DODT/H_2_O/TIPS (91:3:3:3, v/v) for 3 h at room temperature, and the crude products were precipitated with cold diethyl ether. For bismuth-mediated cyclization, crude peptides were dissolved in DMSO and then diluted into 0.1 M HEPES buffer (pH 8.0) before treatment with 1.2 equiv of BiBr_3_ and 2.5 equiv of tris­(carboxyethyl)­phosphine (TCEP). Excess bismuth was removed by centrifugation. Crude Tat and CPP12 were dissolved in DMF, diluted into a 50:50 (v/v) mixture of water and acetonitrile containing 0.05% TFA. All peptides were purified by reversed-phase HPLC on a Waters C18 column. Peptide purity (>95%) and authenticity were confirmed by LC/MS using a Waters ACQUITY UPLC system equipped with a reversed-phase BEH C18 column (130 Å, 1.7 μm, 2.1 × 100 mm), an SQD2 single quadrupole mass spectrometer, and an electrospray ionization (ESI) source (Figure S1).

Fluorescent labeling of peptides was carried out by incubating HPLC purified cyclic peptides with 1.2 equiv of NF–NHS or TMR–NHS in a 2:1 (v/v) mixture of DMSO and 0.1 M sodium bicarbonate buffer, pH 8.0, for 15–30 min at room temperature. The labeled peptides were purified again by reversed-phase HPLC, and their concentrations were determined spectrophotometrically from the absorbance of TMR at 555 nm (ε = 80,000 M^–1^cm^–1^) or NF at 595 nm (ε = 44,000 M^–1^cm^–1^). For fluorescent labeling of Tat, a 4-methyltrityl (MTT)-protected lysine residue was introduced at the C-terminus during solid phase synthesis. The MTT group was selectively removed by the sequential treatment of the resin with 2% TFA and 1% TIPS in DCM for 10 min each, followed by neutralization with 10% diisopropylethylamine (DIPEA) in DMF and thorough washing with DCM and DMF. The exposed ε-amine was then reacted with 3 equiv of carboxy-naphthofluorescein, 8 equiv of DIC, and 4 equiv of Oxyma Pure for 30 min at 50 °C.

### EDTA Competition Assay

Peptides (50 μM) were incubated with increasing concentrations of EDTA (0–10 equiv) in HEPES buffer (pH 7.4) in ANSI 384-well polypropylene collection plate (100-μL square well, waters Corp) at room temperature. The samples were analyzed on a Waters ACQUITY UPLC system equipped with a reversed-phase C18 column, an SQD2 mass spectrometer, and an ESI source. The column was eluted with a linear gradient system of 5–70% acetonitrile in H_2_O containing 0.1% trifluoroacetic acid (v/v) over 8 min at a flow rate of 0.46 mL/min and elution was monitored at 280 nm. The SQD2 mass spectrometer was operated in positive electrospray ionization (ESI) mode, acquiring full-scan mass spectra over a mass-to-charge (*m*/*z*) range of 100–3050. Capillary voltage and cone voltage were set to 4 kV and 40 V, respectively. The source temperature was set to 150 °C and the desolvation temperature was set to 350 °C.

The amount of remaining Bi^3+^-bound bicyclic peptide at each EDTA concentration was determined by integrating the area underneath the corresponding UPLC peak and compared to that of the peak in the absence of EDTA, which was defined as 100% (Figure S2). The percentage of remaining bicyclic peptide was plotted as a function of EDTA equivalents and the Bi^3+^-binding affinity was assessed by the equivalents of EDTA that resulted in 50% loss of Bi^3+^ ion (IC_50_).

### Cell Culture

HeLa cells were cultured in Dulbecco’s modified eagle media (DMEM) supplemented with 10% fetal bovine serum (FBS) and 1% ampicillin/streptomycin. ARE Reporter (Luc)-HepG2 cells were cultured in eagle’s minimal essential media (EMEM) supplemented with 10% FBS and 1% ampicillin/streptomycin. All cells were grown at 37 °C in a humidified incubator in the presence of 5% CO_2_.

### Flow Cytometry

HeLa cells were seeded in a tissue culture-treated 12-well plate at a density of 15 × 10^4^ cells per well and allowed to adhere overnight. The next day, cells were washed twice with Dulbecco’s phosphate-buffered saline (DPBS) and incubated with 2 μM NF-labeled peptides for 2 h at 37 °C in DMEM supplemented with 1% FBS. After incubation, cells were washed twice with cold DPBS and detached using 0.25% trypsin. The cell suspension was diluted in DPBS and pelleted by centrifugation at 300 g at 4 °C for 5 min, washed twice with cold DPBS and resuspended in 200 μL of cold DPBS. Flow cytometry analysis was performed on a BD FACS LSR II instrument using 633 nm excitation and the florescence emission was analyzed in the APC channel. For each sample, three independent biological replicates were analyzed, with 10,000 live cells acquired per replicate. The data were analyzed using FlowJo software.

### ARE Luciferase Reporter Assay

ARE-reporter HepG2 cells^33^ were seeded in 96-well plates at a density of 5,000 cells per well in EMEM supplemented with 10% FBS and allowed to adhere overnight. The following day, cells were washed with DPBS and incubated with varying concentrations of peptide (0–10 μM) for 18 h in EMEM in the presence of 10% FBS. After incubation, 100 μL of BPS One-step luciferase assay reagent was added to each well, and the plates were gently shaken on an orbital shaker in the dark for 20 min before the luminescence was recorded on a Tecan Infinite M1000 Pro plate reader. Luminescence values were normalized to background and analyzed using GraphPad Prism 6.0.

### Serum Stability

Peptide stability in human serum was assessed by incubating peptides (final concentration 100 μM) in 25% (v/v) human serum prepared in PBS (pH 7.4) at 37 °C. At varying time points between 0 and 24 h, 100-μL aliquots were withdrawn and immediately quenched with 200 μL of 7.5% (w/v) trichloroacetic acid prepared in a 1:1 (v/v) mixture of methanol and acetonitrile. Samples were thoroughly mixed on a vortex device and centrifuged at 15,000g for 5 min to precipitate serum proteins, and the clarified supernatants were analyzed by UPLC–MS. The area under the peak corresponding to the intact peptide was integrated and plotted as a function of incubation time to generate serum stability profiles.

### Cytotoxicity

HeLa cells were seeded in 96-well plates at a density of 5000 cells per well in DMEM supplemented with 10% FBS and allowed to adhere overnight. On the following day, the culture medium was replaced with 100 μL of fresh medium containing peptide at the indicated concentrations (0–50 μM), and the cells were incubated at 37 °C for 24 h. Cell viability was then assessed by adding 100 μL of CellTiter-Glo 2.0 reagent to each well, followed by gentle shaking on an orbital shaker for 20 min at room temperature to induce cell lysis and stabilize the luminescent signal. Luminescence was recorded using a Tecan Infinite M1000 Pro plate reader.

### Confocal Microscopy

HeLa cells were seeded in 10/35 mm glass-bottom dishes at a density of 5 × 10^4^ cells/mL in DMEM supplemented with 10% fetal bovine serum (FBS) and 1% penicillin–streptomycin and allowed to adhere overnight. The following day, cells were washed twice with DPBS and treated with fluorescently labeled peptide at the indicated concentrations in DMEM containing 1% FBS and 1% penicillin–streptomycin. Cells were incubated for 2 h at 37 °C, washed twice with DPBS, and counterstained with Hoechst 33342 (Thermo Fisher Scientific; final concentration, 5 μg/mL) for 10 min at 37 °C. Cells were then maintained in phenol red-free DMEM supplemented with 1% FBS for imaging. Live-cell imaging was performed immediately using a Nikon A1R confocal laser-scanning microscope (ECLIPSE Ti-E inverted platform) equipped with an environmental chamber maintained at 37 °C and 5% CO_2_. Images were acquired using a 60x water-immersion objective, with laser power, detector gain, and acquisition settings held constant across all samples. Image processing and analysis were conducted using Nikon NIS-Elements AR software.

### Maximum Tolerated Dose (MTD) Determination in Mice

All animal experiments were conducted in accordance with institutional animal care and use guidelines under committee-approved protocols. Mice were housed in a controlled environment under a 12 h light–dark cycle at 20–25 °C and 40–60% relative humidity, with ad libitum access to food and water. Animals were acclimated for at least 1 week prior to experimentation and housed by sex in groups of five per cage. To assess dose-dependent toxicity, 6–8-week-old BALB/c mice (Jackson Laboratory, strain #000651) underwent a preliminary intravenous (IV) dose-escalation toxicity study. Following peptide administration, mice were continuously monitored for 1 h for signs of acute adverse effects, including hypoactivity, hunched posture, abnormal respiration, reduced responsiveness to stimuli, tremors, convulsions, or seizures. Animals were further observed for delayed toxicity, and body weight and rectal temperature were recorded 24 h postinjection. Groups of three mice were administered escalating doses of peptide in separate cohorts to identify the maximum tolerated dose (MTD), defined as the highest dose that did not result in mortality or severe adverse effects requiring euthanasia.

## Results and Discussion

### Effect of Ring Sizes on Bi^3+^-Binding Affinity

BCP16 (Figure 1) consists of the sequence of 2-mercaptoacetyl-d-Arg-Arg-d-Arg-Arg-d-Cys-Nal-Bta-d-Nal-Cys-NH_2_ [where d-Arg (or r) is d-arginine, d-Cys (or c) is d-cysteine, Nal (or Φ) is L-2-naphthylalanine, Bta (or Ψ) is L-3-benzothioenylalanine, and d-Nal (or ϕ) is d-2-naphthylalanine].^32^ The peptide precursor was rapidly and quantitatively converted into the bicyclic structure through the strong interaction between the three thiol groups and a Bi^3+^ ion.^34^ BCP16 features high cytosolic delivery efficiency, excellent proteolytic stability (*t*_1/2_ > 24 h in human serum), and synthetic accessibility. However, BCP16 was more cytotoxic to HeLa cells than cyclic CPP12 (Figure 1),^22^ despite their similar amino acid sequences (both contain four arginine residues). We hypothesized that the highly efficient cell entry of BCP16 might result in the accumulation of intracellular Bi^3+^ ions and inhibition of cellular proteins. To test this hypothesis and minimize the cytotoxicity of BCPs, we set out to design BCPs with enhanced Bi^3+^-binding affinity. We reasoned that tighter binding of Bi^3+^ would reduce the amount of Bi^3+^ loss from internalized BCPs and potentially the cytotoxicity of the BCPs. Stronger Bi^3+^ binding would also increase the proteolytic stability of BCPs, both during circulation and within the intracellular environment.

To determine the impact of ring sizes on Bi^3+^ binding, we designed and synthesized a panel of bismuth-cyclized peptides of variable loop sizes and of the sequence CX_m_CX_n_C, where C is l-cysteine, X represents l-alanine, l-tyrosine, or l-arginine, and m, n = 2–4 (Table 1, peptides **1**–**5**). The Bi^3+^-binding affinities of peptides **1**–**5** were assessed by using an EDTA competition assay. Briefly, peptides **1**–**5** were incubated with increasing molar equivalents of EDTA in a HEPES buffer (pH 7.4), and the amount of remaining bismuth-bound bicyclic peptide was quantified by UPLC and compared to that of the bicyclic peptide in the absence of EDTA. The results show that the Bi^3+^ binding affinity is inversely correlated with the size of the loops (Table 1 and Figure 2). Thus, peptide **1**, which contains the smallest loop sizes (m, n = 2), exhibited the highest binding affinity for Bi^3+^, requiring 2.3 equiv of EDTA to remove 50% Bi^3+^ from the peptide (IC_50_ = 2.3), whereas peptide **5** (m = 3; n = 4) had the lowest IC_50_ value of 1.2 equiv of EDTA. This result is not unexpected, as larger loops are more conformationally flexible and incur greater entropic loss during cyclization.

**Table 1:** Sequences and Bi^3+^-Binding of Bicyclic Peptides

peptide	sequence	X_m_X_n_	EDTA IC_50_ (equiv)
**1**	CARCYRC	2, 2	2.3
**2**	CARCAYRC	2, 3	2.1
**3**	CARCAAYRC	2, 4	1.7
**4**	CAARCAYRC	3, 3	1.4
**5**	CAARCAAYRC	3, 4	1.2

**Figure 2:**
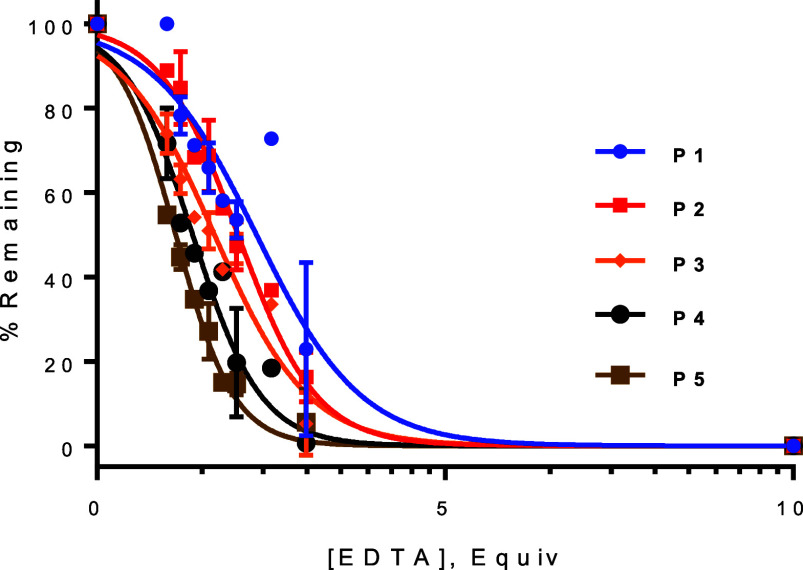
Bi^3+^-binding affinity of peptides **1**–**5** (50 μM) as determined by an EDTA competition assay. Data shown represent the average of two independent sets of experiments.

### Effect of Peptide Sequence on Bi^3+^-Binding Affinity and BCP Properties

Having established the peptide **1** scaffold (m, n = 2) as optimal for Bi^3+^ binding, we next attempted to integrate the BCP16 sequence into the scaffold. Since BCP16 contains four Arg/d-Arg residues and three aromatic hydrophobic residues, we placed an l-Arg-l-Arg motif into the N-terminal loop and the l-Nal-l-Bta sequence into the C-terminal loop, anticipating that cyclization would protect these sequences from proteolytic degradation. For the remaining three residues, we placed two d-Arg residues to the N-terminus of the bicyclic core and a d-Nal to its C-terminus, to give BCP18 (Table 2). The d-amino acids are expected to be stable against proteolytic degradation, despite their exocyclic positions. Deletion of one of the N-terminal d-Arg residues gave BCP19, which results in one less positive charge than BCP16/18 and may potentially reduce toxicity. Finally, we replaced the central d-Cys of BCP19 with l-Cys to produce BCP20.

**Table 2:** Sequences, Cytosolic Entry Efficiencies, and Bi^3+^-Binding Affinities of Loop-Optimized BCPs

CPP	sequence^a^	cell entry efficiency (%)^b^	EDTA IC_50_ (equiv)
BCP16	HS-rRrRcΦΨϕC	100	2.6 ± 0.1
BCP18	Ac-rrCRRcΦΨCϕ	163 ± 1	0.80 ± 0.01
BCP19	Ac-rCRRcΦΨCϕ	107 ± 1	0.84 ± 0.01
BCP20	Ac-rCRRCΦΨCϕ	143 ± 4	1.7 ± 0.2
CPP12	cyclo(FfΦRrRrQ)	65 ± 3	n/a

a All peptides contained a C-terminal miniPEG-Lys­(NF). Ac, acetyl group; HS, 2-mercaptoacetyl; Φ, l-2-napththylalanine; ϕ, d-2-napththylalanine; Ψ, l-3-benzothioenylalanine; r, d-arginine; f, d-phenylalanine; c, d-cysteine.

b All values are relative to those of BCP16 (100%) and represent the mean ± SD of three independent experiments.

To evaluate the cytosolic entry efficiencies of BCP16–20, a lysine residue was appended to the C-terminus of all peptides via a miniPEG linker and the lysine side chain was labeled with NF, a pH-sensitive fluorophore (p*K*_a_ ≈ 7.8) that is essentially nonfluorescent inside the acidic endosomal and lysosomal compartments (pH 4.5–6.5) but becomes highly fluorescent upon entering the neutral environment of the cytosol and nucleus (pH ∼7.4).^35^ HeLa cells were incubated with 2 μM NF-labeled BCPs for 2 h and analyzed by flow cytometry. The mean fluorescence intensity (MFI) of the cells provides a convenient readout of the amount of peptide that has reached the cytosol. Gratifyingly, BCP18–20 retain or have improved cell-penetrating activities, exhibiting cytosolic entry efficiencies of 163%, 107%, and 143%, respectively, relative to BCP16 (100%) (Table 2 and Figure S3). As a comparison, CPP12 showed a cytosolic entry efficiency of 65% under the same condition.

The Bi^3+^-binding affinities of BCP16–20 (unlabeled) were evaluated by using the EDTA competition assay. BCP16 has the highest bismuth-binding affinity, exhibiting an IC_50_ value of 2.6 equiv of EDTA (Figure 3). BCP18–20 showed IC_50_ values 0.80, 0.84, and 1.7 equiv of EDTA, respectively (Table 2). The cytotoxicity of BCP18–BCP20 was assessed by monitoring their effect on the viability of HeLa cells. BCP18–20 showed IC_50_ values of 28, 30, and 32 μM, respectively, which are moderately higher than that of BCP16 (IC_50_ ≈ 19 μM) (Figure 4). Thus, BCP20 has a favorable balance of properties including higher cell entry efficiency and lower cytotoxicity, while its Bi^3+^ binding affinity remains somewhat weaker than that of BCP16. Unfortunately, BCP20 is proteolytically labile, showing a *t*_1/2_ of 2.3 h in human serum (Figure S4). This reduced stability likely stems from the inclusion of seven consecutive l-amino acids (Cys-Arg-Arg-Cys-Nal-Bta-Cys) and a decreased binding affinity for the Bi^3+^ ion (relative to BCP16). Additional modifications of BCP20 failed to further increase the Bi^3+^-binding affinity or improve its overall properties.

**Figure 3:**
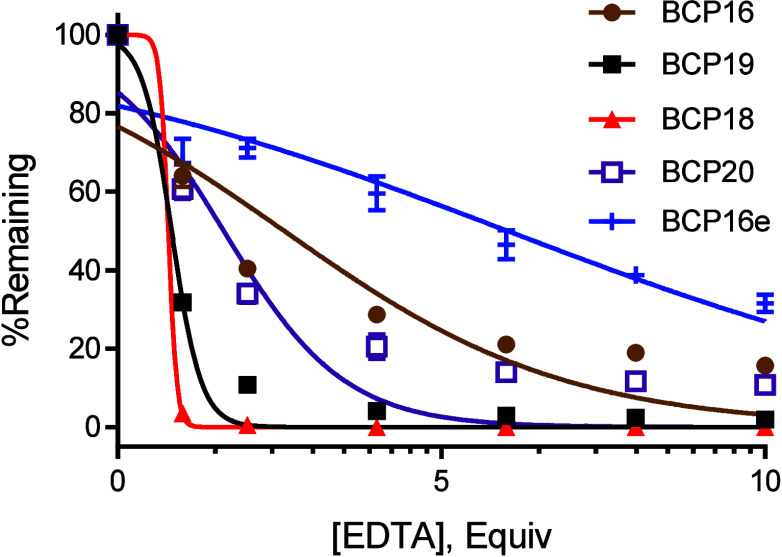
Bi^3+^-binding affinity of BCP16–20 and BCP16e (50 μM) as determined by the EDTA competition assay. Data shown represent the mean and standard deviation of two independent sets of experiments (*n* = 3).

**Figure 4:**
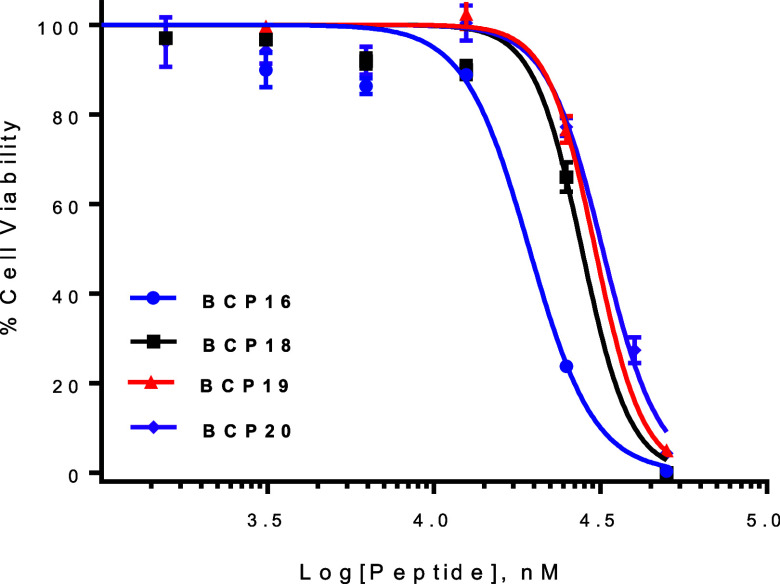
Effect of BCP16 and BCP18–BCP20 on the viability of HeLa cells. All values reported are relative to those of the vehicle (no peptide, 100%) and represent the mean and standard deviation of three independent sets of experiments (*n* = 3).

Our results demonstrate that Bi^3+^ coordination is governed by not only the loop sizes but also the sequence-dependent peptide conformation, which preorganizes the thiol ligands into a favorable three-dimensional binding geometry. They also highlight the considerable challenges in balancing the different (and often conflicting) properties of an effective CPP. Since BCP16 exhibited the highest Bi^3+^-binding affinity among the BCPs tested, high cell entry efficiency, and excellent proteolytic stability, we decided to focus our subsequent efforts on reducing the number of positive charges (and potentially the toxicity) of BCP16, instead of further optimization of BCP20.

### Optimization of BCP16

To reduce the number of positive charges and potentially toxicity, we replaced each of the arginine residues of BCP16 individually with a neutral isostere, l- or d-citrulline, to produce BCP16a-d (Table 3). Replacement of the second or third Arg/d-Arg reduced the cytosolic entry efficiency by ∼2-fold (BCP16b and BCP16c); on the other hand, substitution of l- or d-citrulline for the first or fourth arginine had little effect on the cell entry efficiency (92% and 102% cytosolic entry efficiencies for BCP16a and BCP16d, respectively; Figure S5). We next replaced both the first and fourth arginine residues with the corresponding d- and l-citrulline to generate BCP16e, which carries a +2 charge under physiological pH. Surprisingly, BCP16e exhibited a 2.2-fold higher cytosolic entry efficiency (Table 3 and Figure S5) as well as 2.3-fold tighter binding to Bi^3+^ than BCP16 (IC_50_ of 6.1 equiv of EDTA for BCP16e; Figure 3). The greater Bi^3+^-binding affinity is likely due to a reduction in repulsive interactions when arginine residues are replaced with citrullines.

**Table 3:** Sequences and Cytosolic Entry Efficiencies of BCP16 Analogs

CPP	sequence^a^	cytosolic entry efficiency (%)^b^
BCP16	HS-rRrRcΦΨϕC	100
BCP16a	HS-δRrRcΦΨϕC	92 ± 4
BCP16b	HS-rΔrRcΦΨϕC	49 ± 1
BCP16c	HS-rRδRcΦΨϕC	45 ± 2
BCP16d	HS-rRrΔcΦΨϕC	102 ± 2
BCP16e	HS-δRrΔcΦΨϕC	222 ± 10

a All peptides contained a C-terminal miniPEG-Lys­(NF). HS, 2-mercaptoacetyl; Φ, l-2-napththylalanine; ϕ, d-2-napththylalanine; Ψ, l-3-benzothioenylalanine; δ, d-citrulline; Δ, l-citrulline; r, d-arginine; c, d-cysteine.

b All values are relative to that of BCP16 (100%) and represent the mean ± SD of three independent experiments.

The cell entry of BCP16e was further confirmed and compared with BCP16 and CPP12 by confocal microscopy of HeLa cells treated with the fluorescently labeled peptides. Cells treated with 2 μM TMR-labeled BCP16 (BCP16^TMR^) or BCP16e (BCP16e^TMR^) showed intense fluorescence throughout the cell volume including the nucleus, whereas cells treated with CPP12^TMR^ had weaker and predominantly punctate signals (Figure 5). The presence of strong TMR fluorescence inside the nucleus demonstrates that a significant fraction of the internalized BCP16 and BCP16e reached the cytosol. We also treated HeLa cells with 2 μM NF-labeled BCP16 (BCP16^NF^), BCP16e (BCP16e^NF^), or CPP12 (CPP12^NF^) and examined their intracellular distribution by confocal microscopy. Interestingly, BCP16e^NF^ exhibited strong and diffuse fluorescence throughout the cell volume, while BCP16^NF^ produced strong but mostly punctate signals (Figure S6). The fluorescence pattern of CPP12^NF^ was somewhere between those of BCP16^NF^ and BCP16e^NF^; it showed diffuse fluorescence within the nucleus but punctate signals in the cytoplasmic region. Since NF is only weakly fluorescent inside the acidic endolysosomal compartments, the observed intracellular NF fluorescence should represent peptides that have successfully escaped the endosome into the cytosol. A plausible explanation of the cytosolic puncta is that these CPPs escape the endosome by the VBC mechanism and form initial lipid/peptide aggregates. Depending on the nature of the CPP, rapid dissolution of the aggregates results in diffuse fluorescence inside the cytoplasm and nucleus, whereas slow dissolution manifests as fluorescence puncta inside the cytoplasm.^36^ However, our data does not rule out the possibility that some of the puncta might be from CPPs entrapped inside the endolysosomal compartments. Note that BCP16e^NF^ and, to a less extent, BCP16e^TMR^ also produced fluorescence puncta outside the cells. The latter are likely caused by the limited aqueous solubility of dye-labeled BCP16e and their precipitation when bound to components of the growth media.

**Figure 5:**
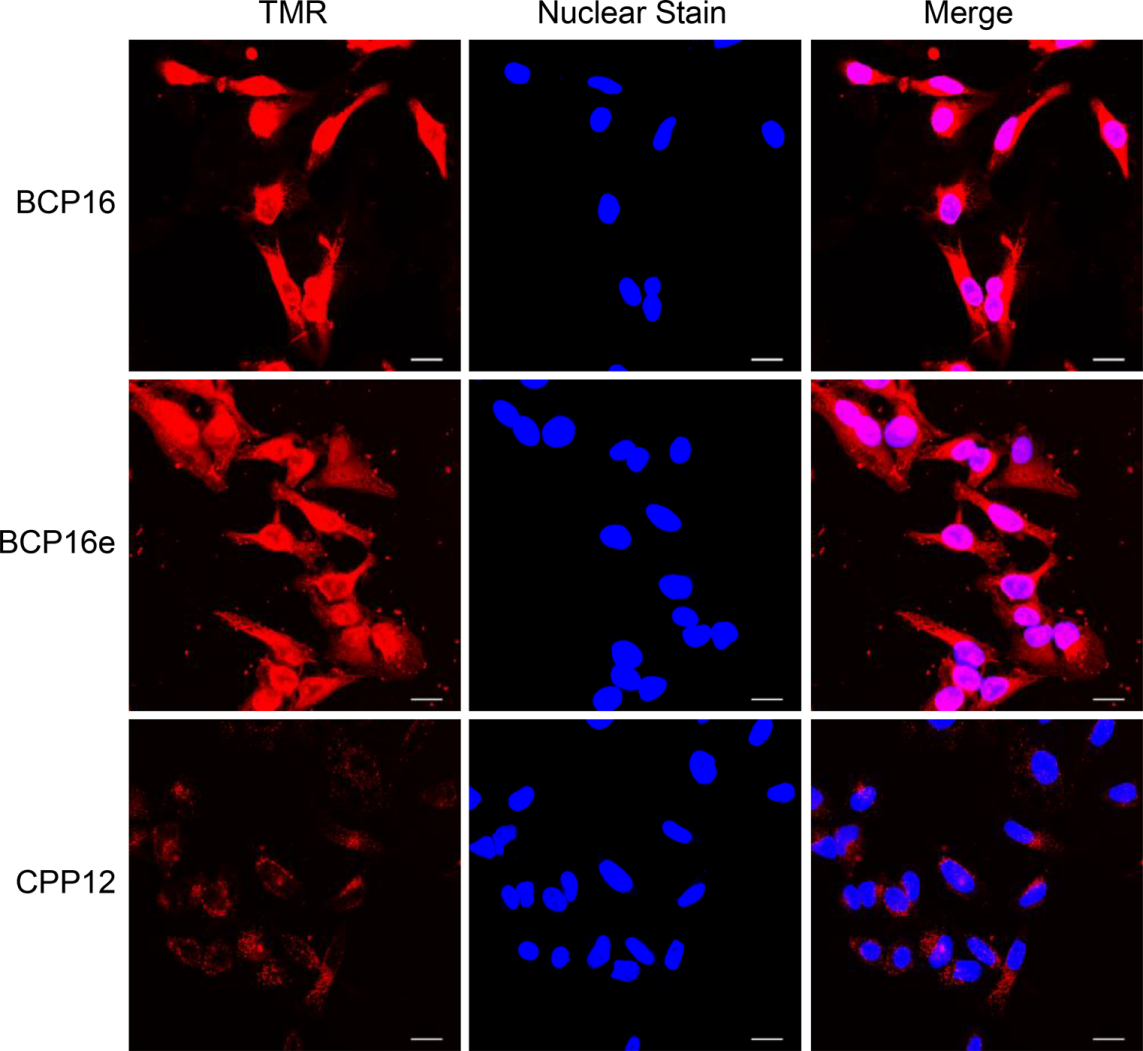
Representative confocal microscopy images of HeLa cells after treatment with 2 μM TMR-labeled BCP16, BCP16e, or CPP12 for 2 h in DMEM supplemented with 1% FBS. Scale bar, 20 μm.

As expected, BCP16a–e showed reduced cytotoxicity toward HeLa cells, with IC_50_ values of 32, 31, 24, 31, and 35 μM, respectively, in the cell viability assay, which are significantly greater than that of BCP16 (19 μM) (Figure 6). BCP16e displayed similar proteolytic stability to BCP16, having a *t*_1/2_ of 25 h in human serum (Figure S4).

**Figure 6:**
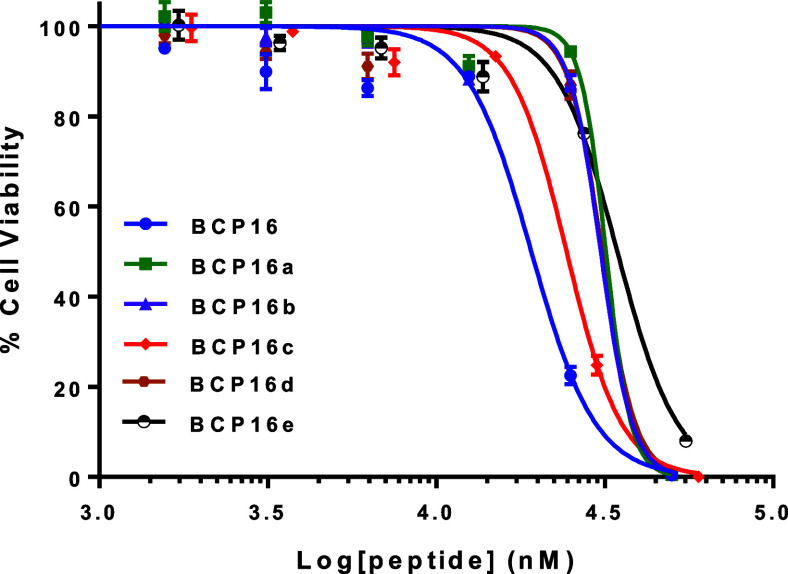
Effect of BCP16 and BCP16a–e on the viability of HeLa cells after 24 h of incubation. Data shown represent the mean and standard deviation of three independent sets of experiments (*n* = 3).

### Intracellular Delivery of Peptidyl Cargo by BCP16e

We next tested the ability of BCP16e to deliver biologically active peptidyl cargo into mammalian cells. To this end, we conjugated BCP16e, BCP16 and CPP12 to a previously reported linear peptidyl inhibitor of the Keap1-Nrf2 interaction, LDPETGEYL (P1, *K*_D_ = 21 nM for Keap1),^37^ via a flexible miniPEG linker (Figure 7A). For comparison, P1, which is membrane impermeable, was synthesized as a negative control. The ability of the peptides to inhibit the intracellular Keap1–Nrf2 interaction was evaluated using an ARE reporter assay in HepG2 cells, which express a firefly luciferase gene under Nrf2-dependent transcriptional control.^33^ Under basal conditions, Nrf2 interacts with Keap1 and is retained in the cytosol or targeted for proteasomal degradation. However, upon blocking the Keap1–Nrf2 interaction, Nrf2 accumulates and translocates into the nucleus, turning on the expression of the luciferase.

**Figure 7:**
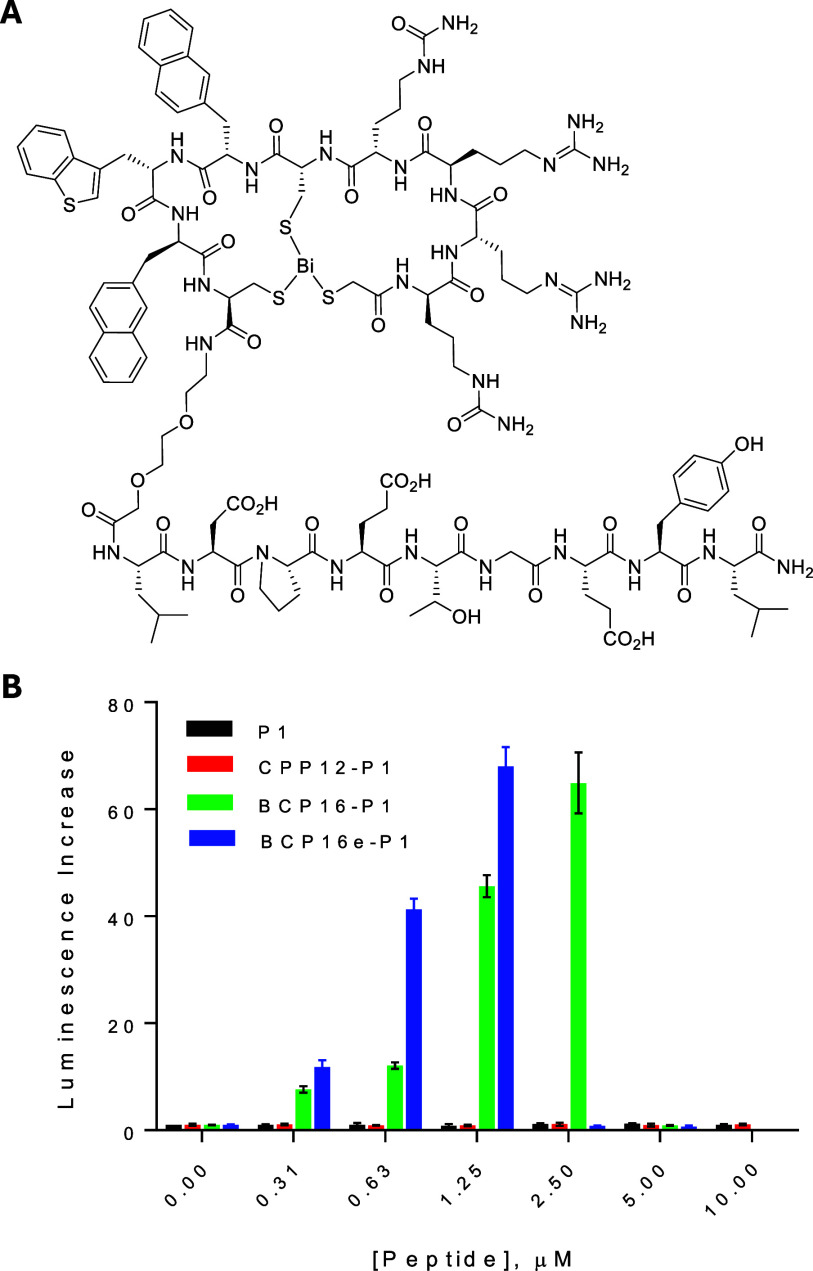
(A) Structure of BCP16e-P1. (B) Inhibition of the Keap1-Nrf2 interaction in HepG2 cells by peptides P1, CPP12-P1, BCP16-P1, and BCP16e-P1 as monitored by the ARE reporter assay in the presence of 10% FBS. Data reported are relative those of control (no peptide) and represent the mean ± SD of three biological replicates (*n* = 3).

HepG2 cells were treated with the peptides in the presence of 10% FBS. As expected for a membrane-impermeable peptide, P1 did not result in a significant increase in luciferase activity at concentrations up to 10 μM (Figure 7B). Consistent with our previous observation that serum proteins strongly bind CPP12 and markedly diminish its delivery efficiency,^32^ the CPP12-P1 conjugate induced only ∼1.1-fold increase in luminescence at 10 μM. In contrast, BCP16-P1 and BCP16e-P1 strongly increased the luciferase expression in a dose-dependent manner. BCP16-P1 induced up to ∼60-fold luciferase activation at 2.5 μM, with an EC_50_ value (concentration at which the half-maximum is reached) of ∼1.1 μM. BCP16e-P1 was ∼2-fold more potent than BCP16-P1, achieving a maximal induction of ∼70-fold at 1.25 μM and an EC_50_ value of ∼0.6 μM. Further increase in the peptide concentration (>2.5 μM for BCP16-P1 and >1.25 μM for BCP16e-P1), however, resulted in a loss of the luminescence induction (Figure 7B). To determine whether this decrease in luminescence was caused by peptide-induced cytotoxicity, we tested the peptides for their effect on the viability of HepG2 cells. BCP16 reduced the viability of HepG2 cells by ∼30% at 10 μM, while BCP16e had no significant effect up to 10 μM (Figure S7). In stark contrast, HepG2 cells lost ∼90% of the viability when the concentration of BCP16e-P1 and BCP16-P1 reached 2.5 and 5.0 μM, respectively. Our results are consistent with the previous report that stable knockdown of *Keap1* expression by shRNA in hepatocarcinoma cell lines induces spontaneous cell toxicity.^38^

### MTD of BCP16 and BCP16e in Mice

To determine the maximally tolerated dose (MTD) of BCP16 and BCP16e, a dose-escalation study was conducted in BALB/c mice (n = 3 per group) via intravenous administration. Animals were monitored for 7 days for signs of clinical toxicity and body weight fluctuations. At doses up to 3 mg/kg, no significant adverse effects, mortality, or behavior changes were observed for either peptide. At 10 mg/kg, both peptides resulted in transient hypoactivity within 5–10 min postinjection, although mice remained responsive to external stimuli and moved upon provocation. These effects resolved within ∼45 min, with animals returning to baseline activity levels. No evidence of acute toxicity was observed. However, at the 15 mg/kg level, mice injected with BCP16 displayed severe adverse effects characterized by hypoactivity, labored breathing, and responsiveness only to provocation for up to 1 h. All three animals died within 1–2 h after injection. Based on these observations, the LD_50_ of BCP16 lies between 10 and 15 mg/kg. Mice dosed with BCP16e at 15 mg/kg, the highest dose evaluated due to solubility limitations, exhibited similar behavior to the 10 mg/kg group and no mortality was observed after 7 days, indicating a LD_50_ of >15 mg/kg. This LD_50_ value is similar to those reported for Tat^29^ and R8,^30^ despite a 4-fold reduction in the number of positive charges, indicating that other structural moieties (e.g., aromatic hydrophobic residues) also contribute to off-target binding and toxicity. However, the vastly improved cytosolic delivery efficiency of BCP16e relative to Tat and R_8_ (by ∼100-fold) should provide a much greater therapeutic index over the linear CPPs.

## Conclusions

In this work, we discovered that the loop sizes and the peptide sequence interdependently impact the Bi^3+^-binding affinity of peptides containing three thiol groups and consequently their cell-penetrating activity, proteolytic stability, and toxicity. However, by replacing the less critical arginine residues of BCP16 with citrulline, we were able to obtain BCP16e as a highly potent and proteolytically stable CPP of superior safety profile. BCP16e may provide a general vehicle for the intracellular delivery of membrane-impermeable biomolecules. Importantly, our study demonstrates that high cationic charge is not a prerequisite for efficient membrane translocation.

## Supplementary Material



## Notes

Analytical data of peptides used in this work, raw data for Bi^3+^-binding assay and flow cytometry, and serum stability and cytotoxicity of various peptides (PDF)

## ABBREVIATIONS

CPP: cell-penetrating peptide
BCP: bicyclic cell-penetrating peptide
MTD: maximum tolerated dose
NF: naphthofluorescein
TMR: tetramethylrhodamine
