# Supplementary material for: Discovery of a Minimally Charged Cell-Penetrating Peptide
Source: Biochemistry. 2026 Mar 11;65(7):985–93. doi: 10.1021/acs.biochem.6c00003 (PMC13043234; doi:10.1021/acs.biochem.6c00003)
Supplement: Supplementary file 1 [file bi6c00003_si_001.pdf]

Supporting Information

Discovery of a Minimally Charged Cell-Penetrating Peptide

Saikat Mandal, Jeremy L. Ritchey, Prabhat Bhat, and Dehua Pei*

Department of Chemistry and Biochemistry, The Ohio State University, 484 West 12th Avenue, Columbus, Ohio 43210, United States

*E-mail: pei.3@osu.edu

Table of Contents

Figure S1. Structures and analytical data of peptides used in this work…………… …………………….S2

Figure S2. Dissociation of Bi^{3+} from BCPs upon treatment with EDTA…………… ………………......S40

Figure S3. Cytosolic entry efficiency of BCP16-20 ………………………………….…………………S41

Figure S4. Proteolytic stability of BCP20, BCP16 and BCP16e ……………………..…………………S41

Figure S5. Cytosolic entry efficiency of BCP16a-e………………………………...…………………S42

Figure S6. Confocal images of HeLa cells treated with NF-labeled BCPs…………...…………………S43

Figure S7. Effect of BCP16/BCP16e and their P1 conjugates on HepG2 cell viability………..………S44

Figure S1. Structures and analytical data of peptides used in this work. Note: Commercially sourced NF and TMR fluorophores were obtained as mixtures of the 5- and 6-carboxy positional isomers, which produced two distinct peaks in UPLC analyses. When applicable, these isomeric mixtures were used without further separation in the experiments described here.

P1

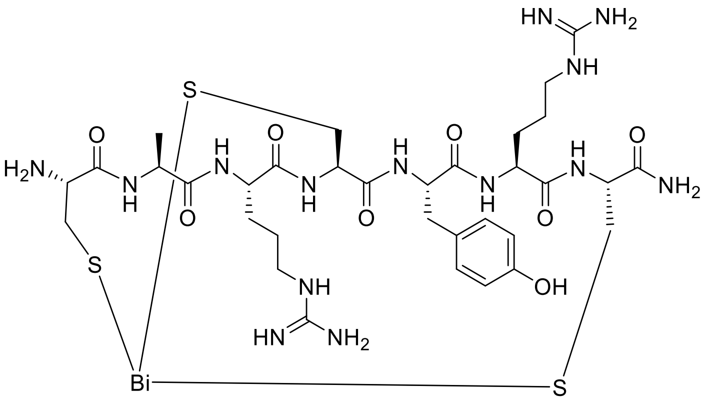

Purity assessment by UPLC (214 nm):

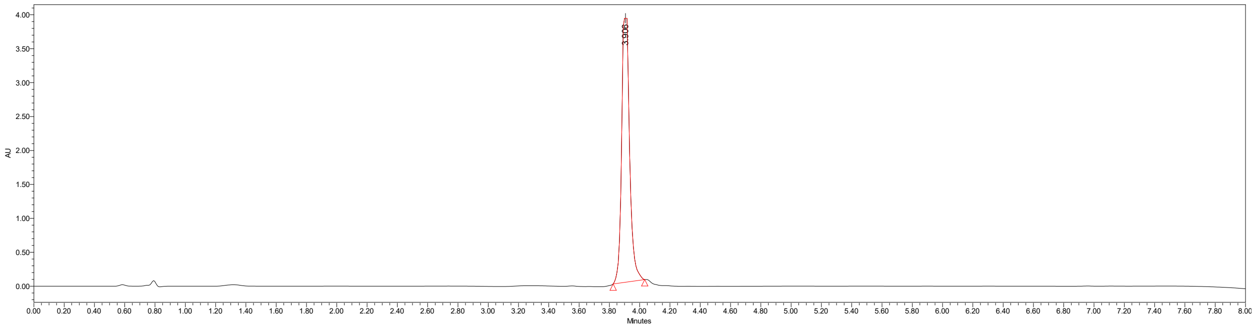

ESI-MS:

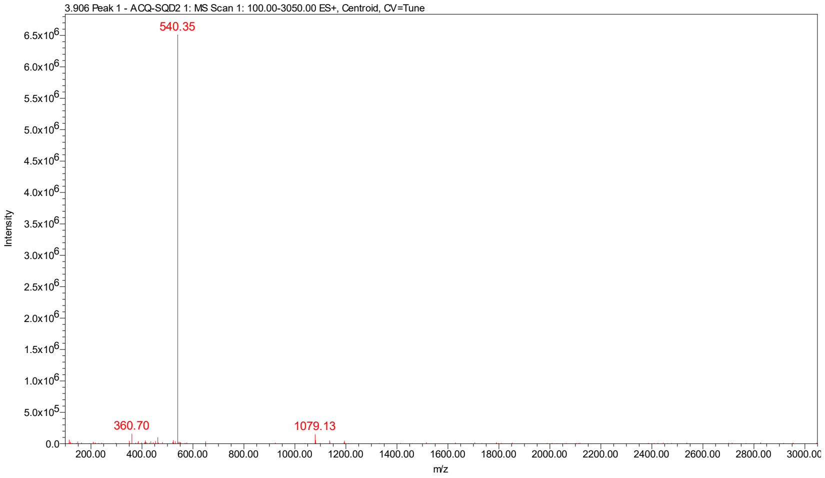

Calculated $[M+3H]^{3+}$: 360.44; observed 360.70
Calculated $[M+2H]^{2+}$: 540.16; observed 540.35
Calculated $[M+H]^{1+}$: 1079.31; observed 1079.13

P2

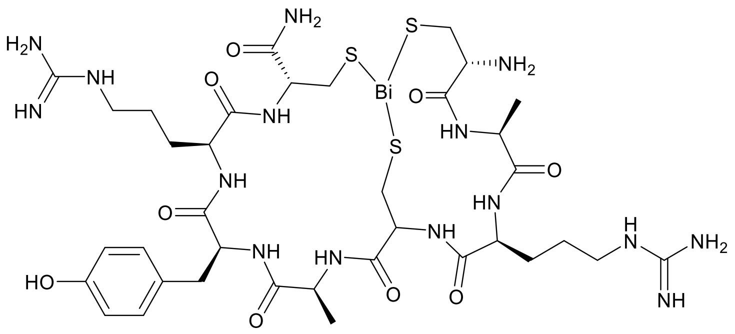

Purity assessment by UPLC (214 nm):

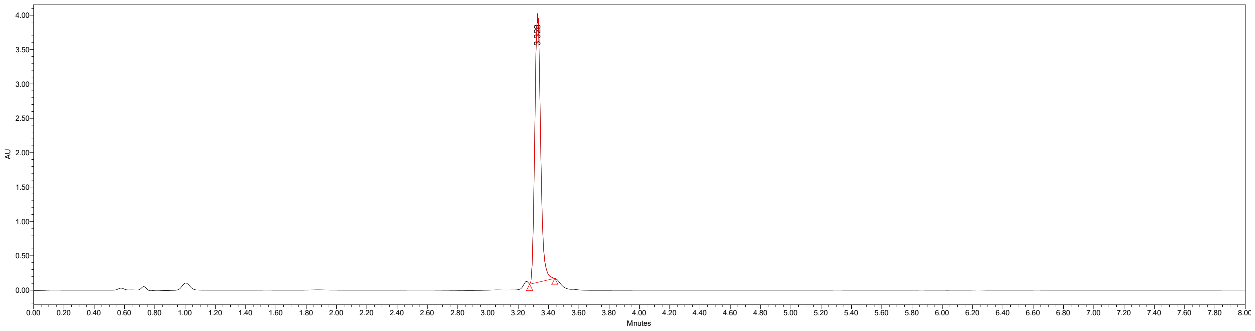

ESI-MS:

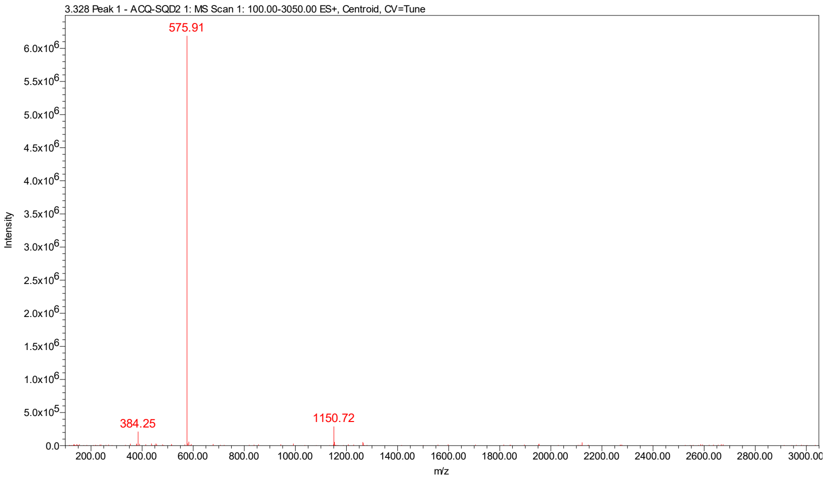

Calculated $[M+3H]^{3+}$:384.12; observed 384.25
Calculated $[M+2H]^{2+}$: 575.68; observed 575.91
Calculated $[M+H]^{1+}$: 1150.35; observed 1050.72

P3

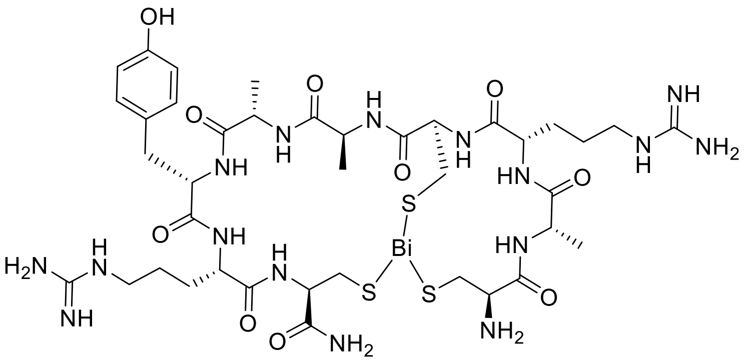

Purity assessment by UPLC (214 nm):

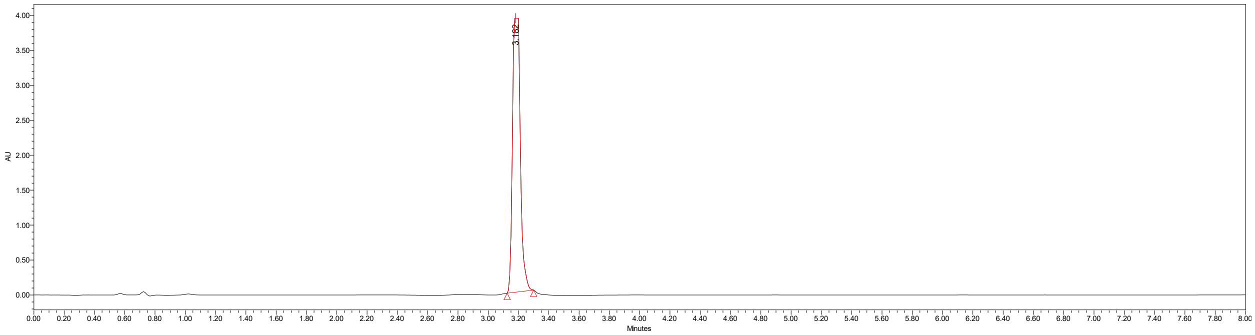

ESI-MS:

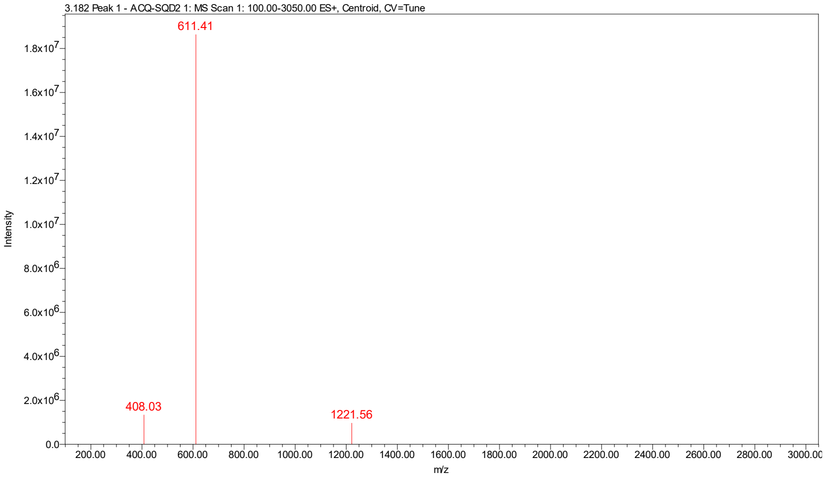

Calculated $[M+3H]^{3+}$:407.80; observed 408.03
Calculated $[M+2H]^{2+}$: 611.20; observed 611.41
Calculated $[M+H]^{1+}$: 1221.39; observed 1221.56

P4

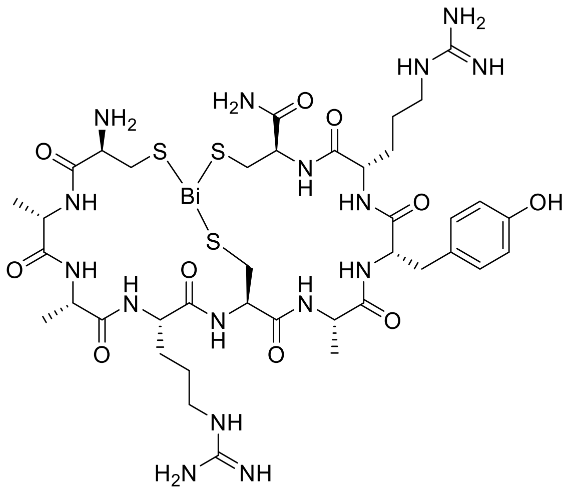

Purity assessment by UPLC (214 nm):

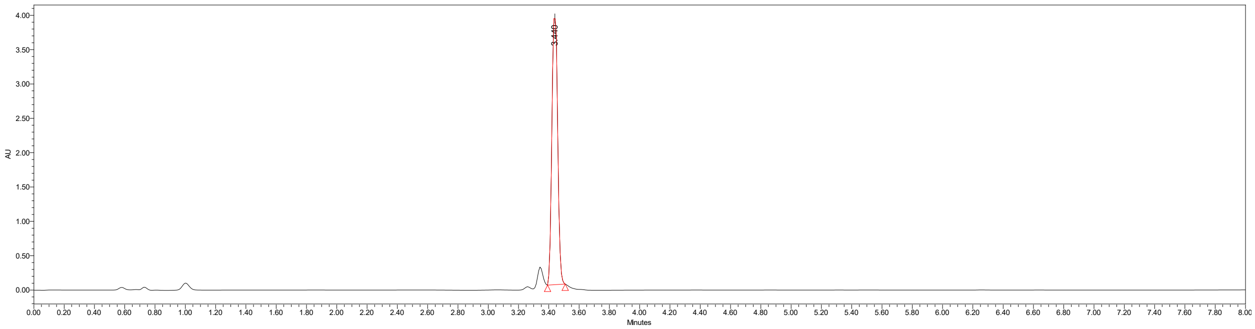

ESI-MS:

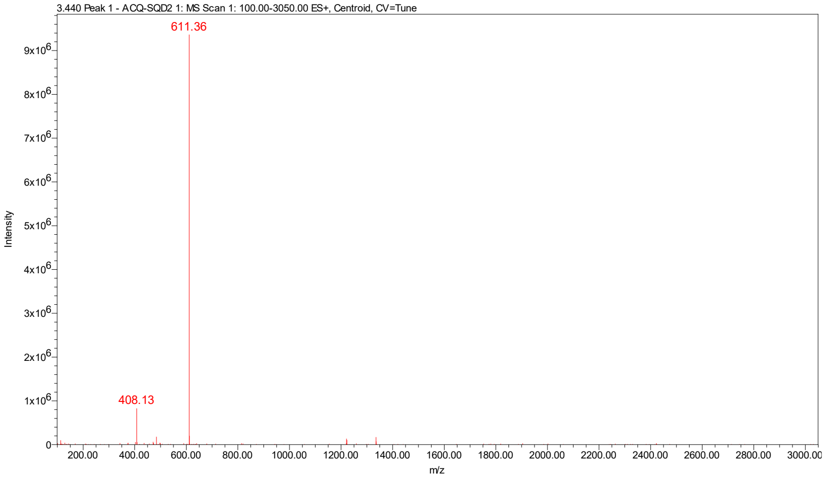

Calculated $[M+3H]^{3+}$:407.80; observed 408.13

Calculated $[M+2H]^{2+}$: 611.20; observed 611.36

P5

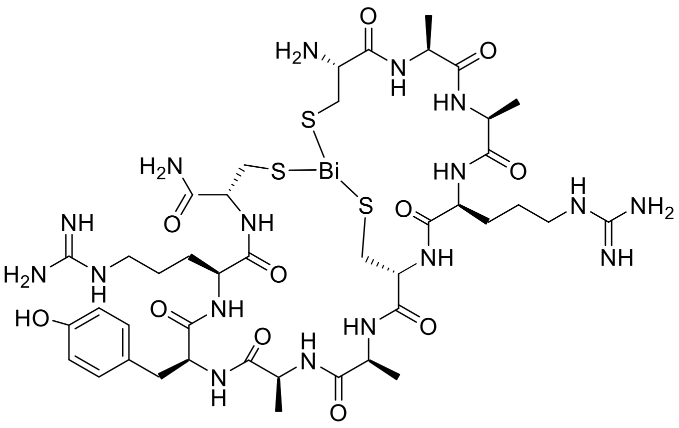

Purity assessment by UPLC (214 nm):

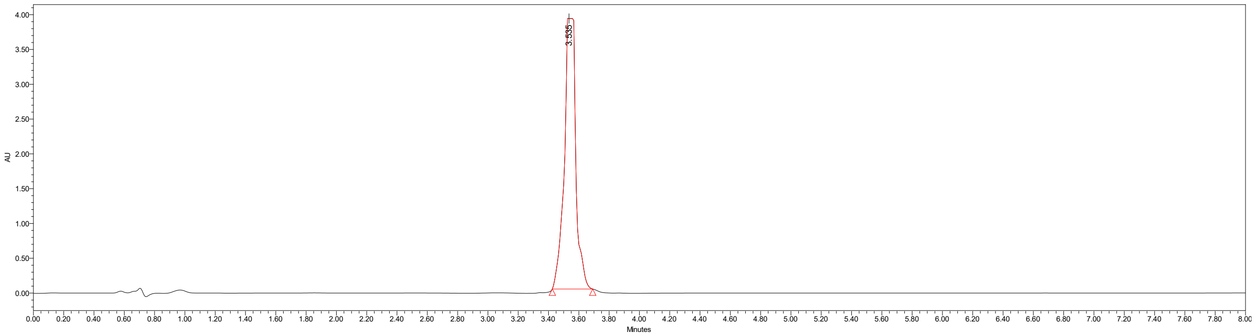

ESI-MS:

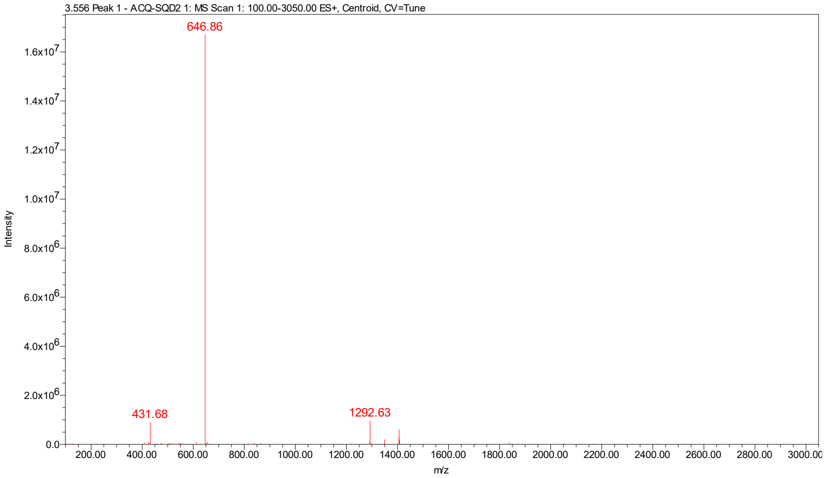

Calculated $[M+3H]^{3+}$:431.48; observed 431.68

Calculated $[M+2H]^{2+}$: 646.72; observed 646.86

Calculated $[M+1H]^{1+}$: 1292.43; observed 1292.63

CPP12

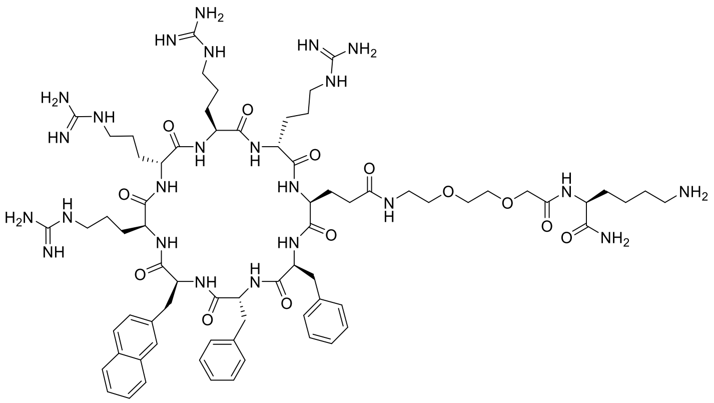

Purity assessment by UPLC (214 nm):

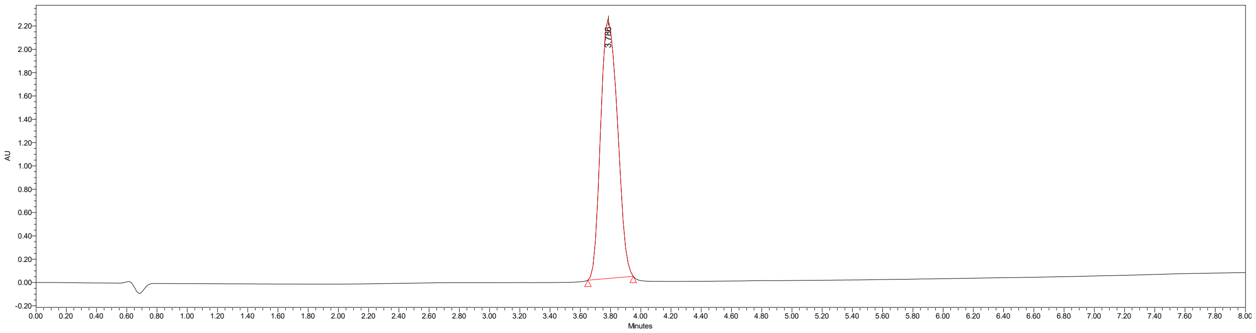

ESI-MS:

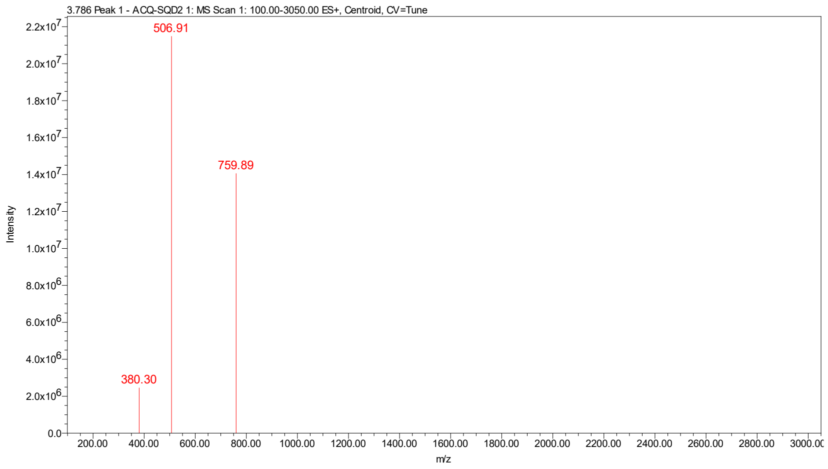

Calculated $[M+4H]^{4+}$: 380.22; observed 380.30
Calculated $[M+3H]^{3+}$: 506.63; observed 506.91
Calculated $[M+2H]^{2+}$: 759.43; observed 759.89

CPP12^NF

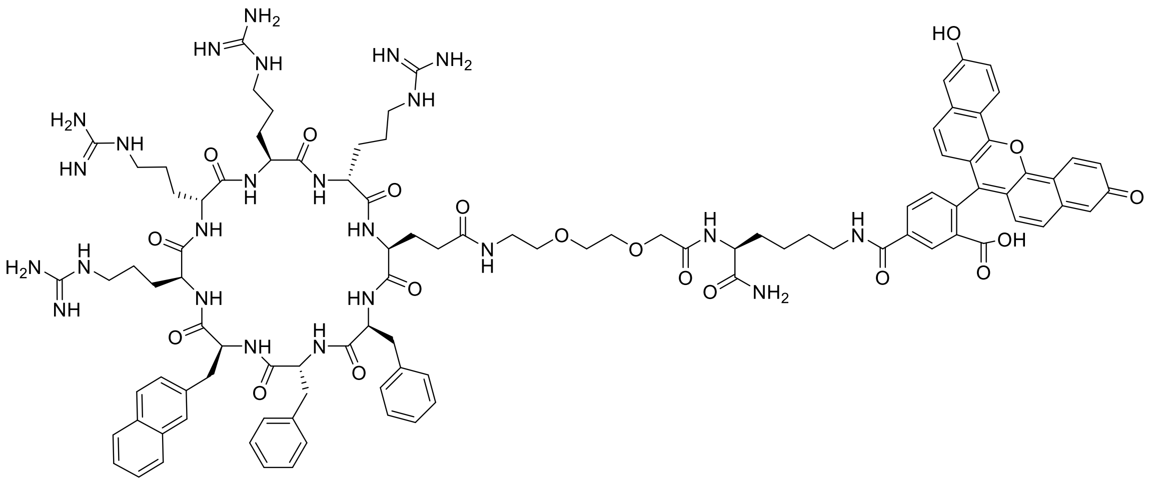

Purity assessment by UPLC (214 nm):

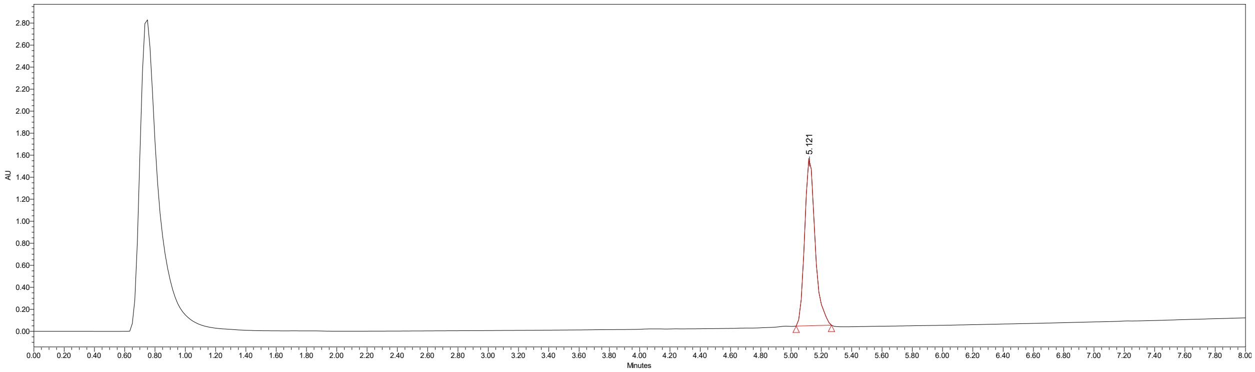

ESI-MS:

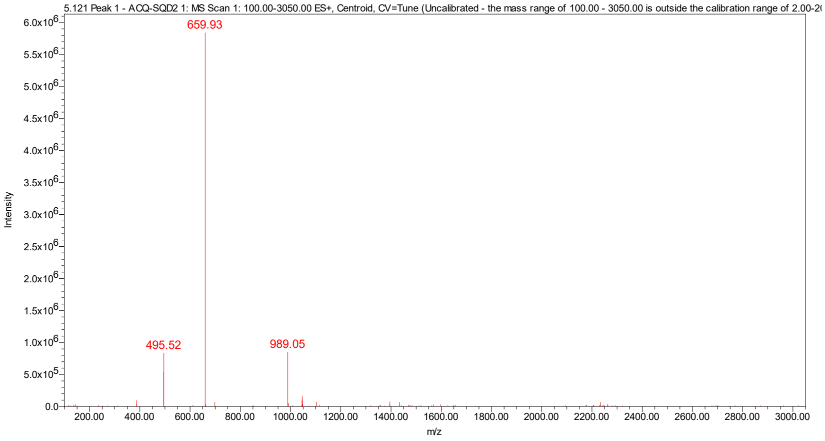

Calculated $[M+4H]^{4+}$:494.73; observed 495.52
Calculated $[M+3H]^{3+}$: 659.31; observed 659.93
Calculated $[M+2H]^{2+}$: 988.47; observed 989.05

CPP12^{TMR}

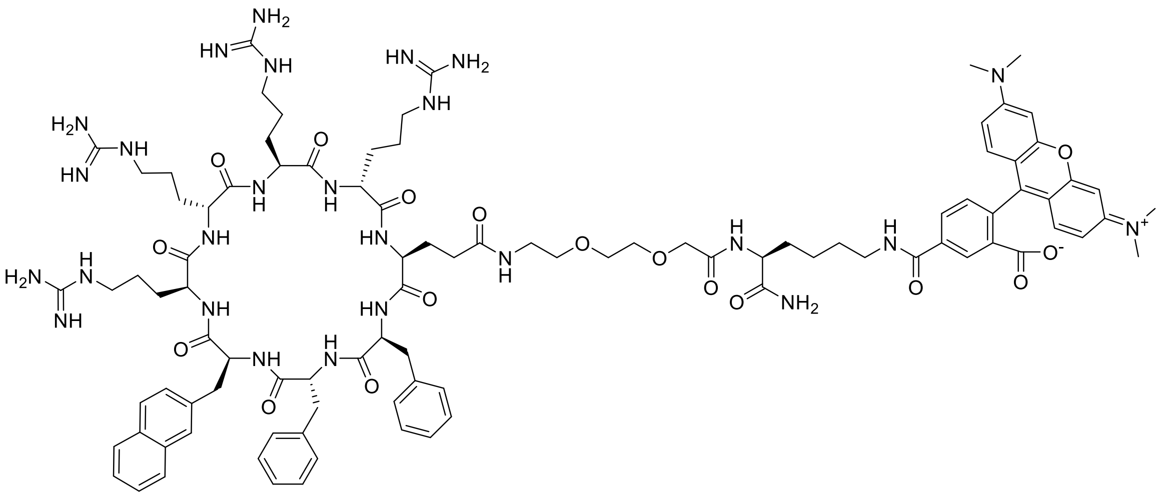

Purity assessment by UPLC (214 nm):

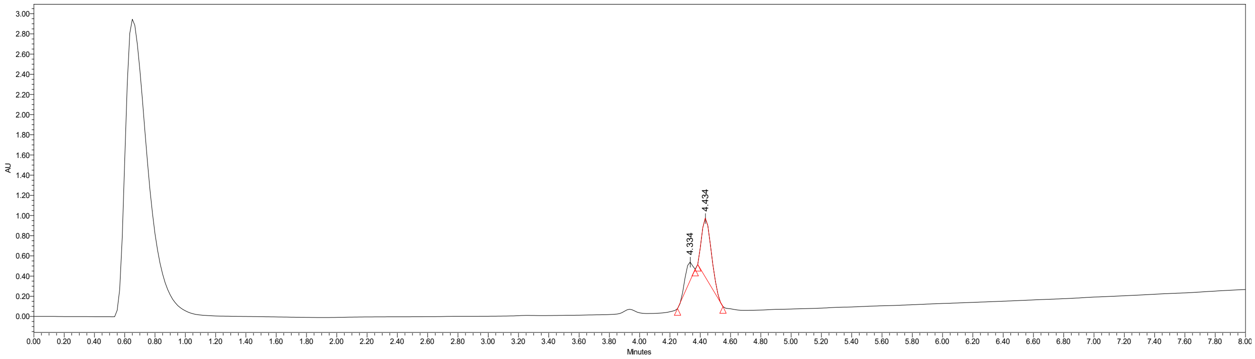

ESI-MS:

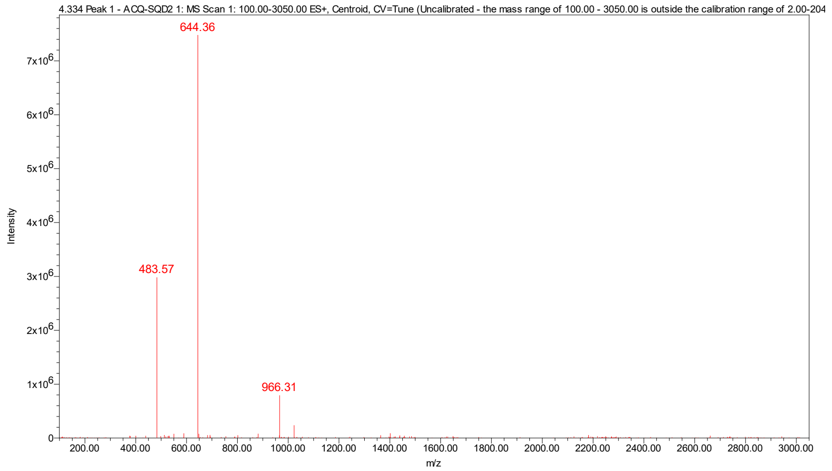

Calculated $[M+4H]^{4+}$:483.25; observed 483.57

Calculated $[M+3H]^{3+}$: 644.00; observed 644.36

Calculated $[M+2H]^{2+}$: 965.5; observed 966.31

BCP18

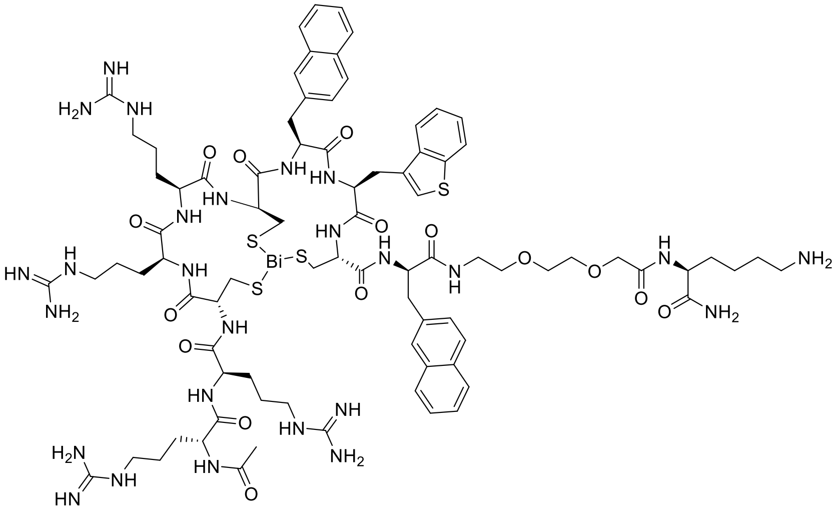

Exact Mass: 2068.8035

Purity assessment by UPLC (214 nm):

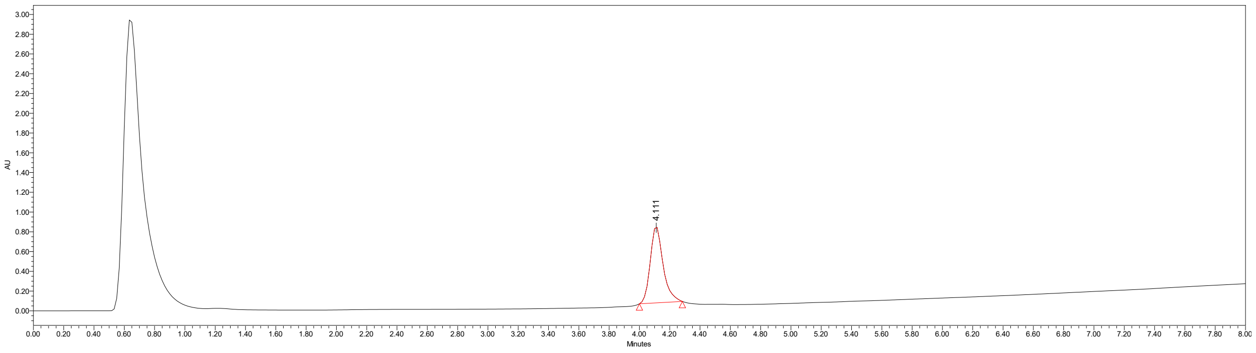

ESI-MS:

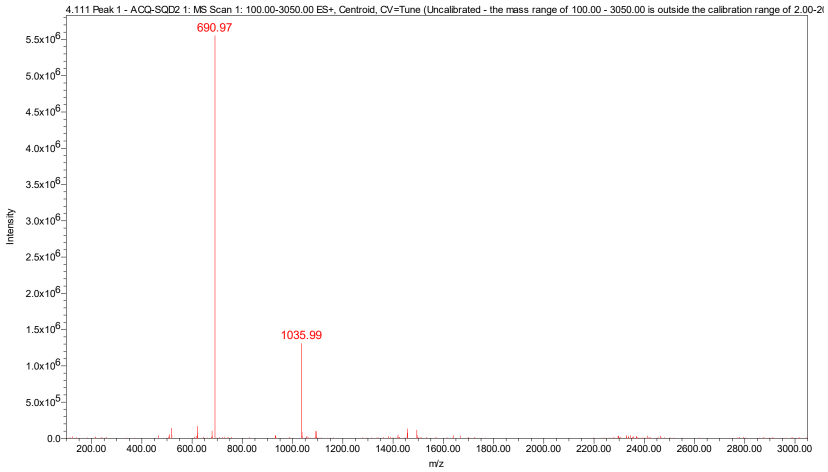

Calculated $[M+3H]^{3+}$: 690.60; observed 690.97
Calculated $[M+2H]^{2+}$: 1035.40; observed 1035.99

BCP18[NF]

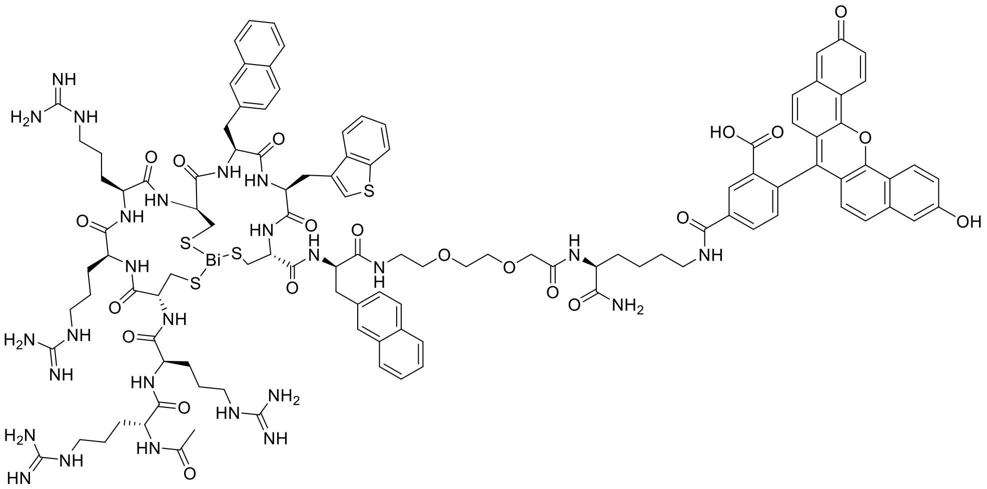

Purity assessment by UPLC (214 nm):

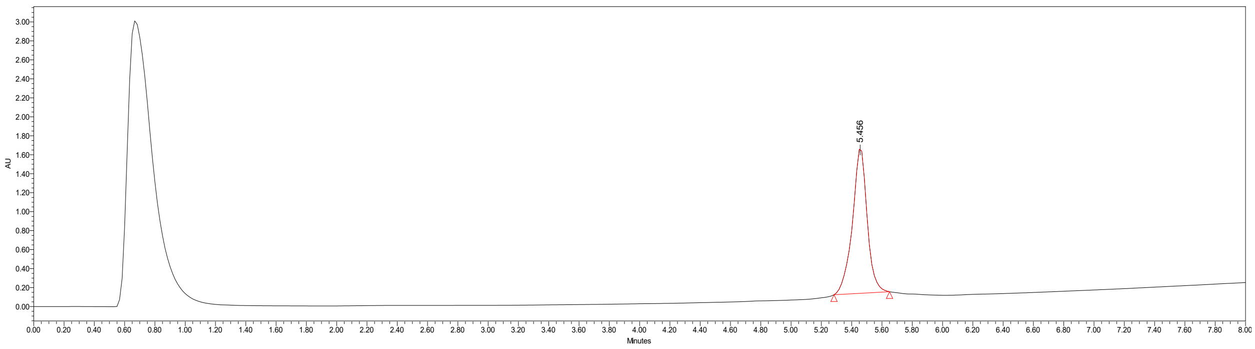

ESI-MS:

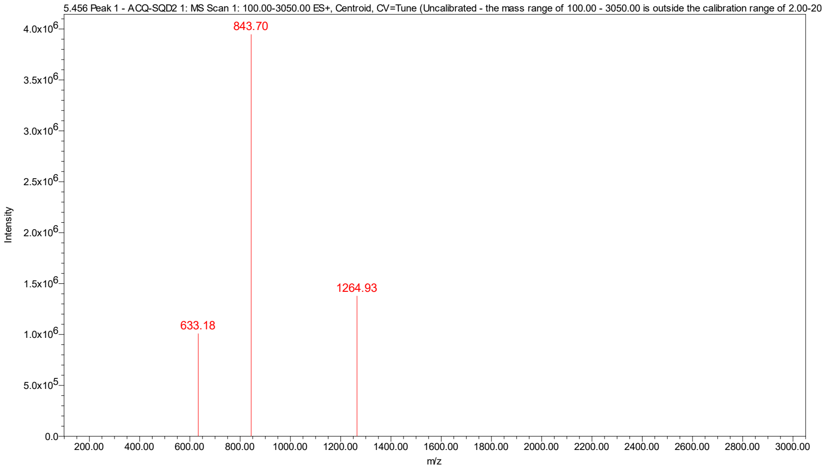

Calculated $[M+4H]^{4+}$:632.72; observed 633.18

Calculated $[M+3H]^{3+}$: 843.29; observed 843.70

Calculated $[M+2H]^{2+}$: 1264.44; observed 1264.93

BCP19

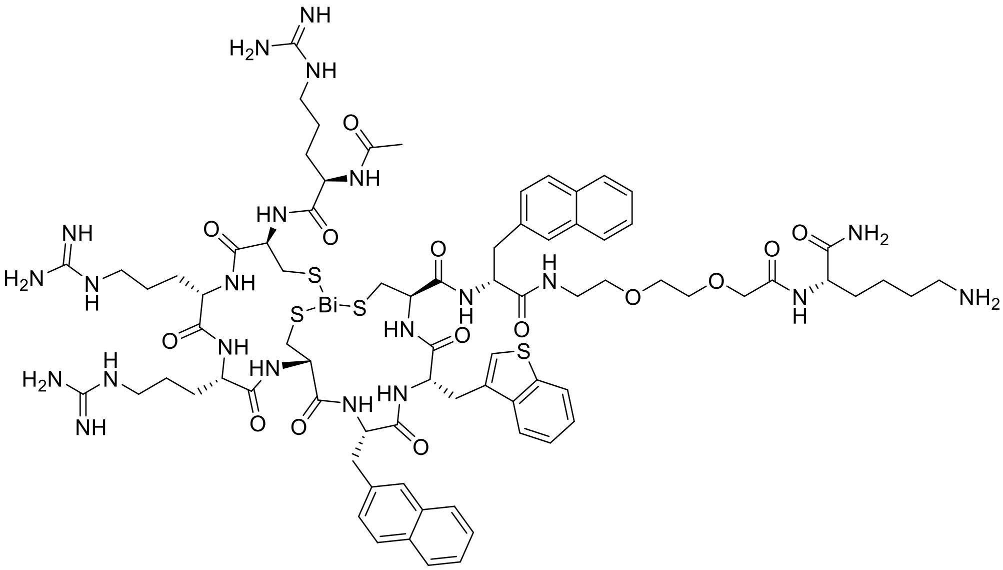

Purity assessment by UPLC (214 nm):

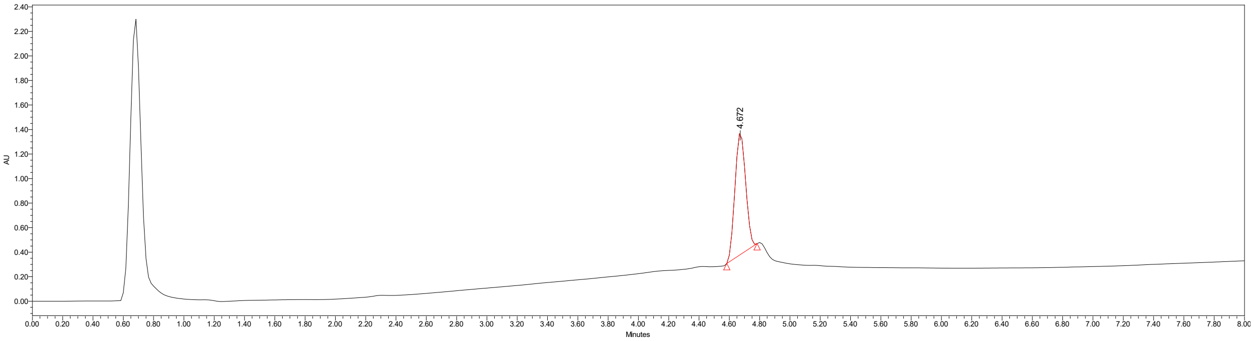

ESI-MS:

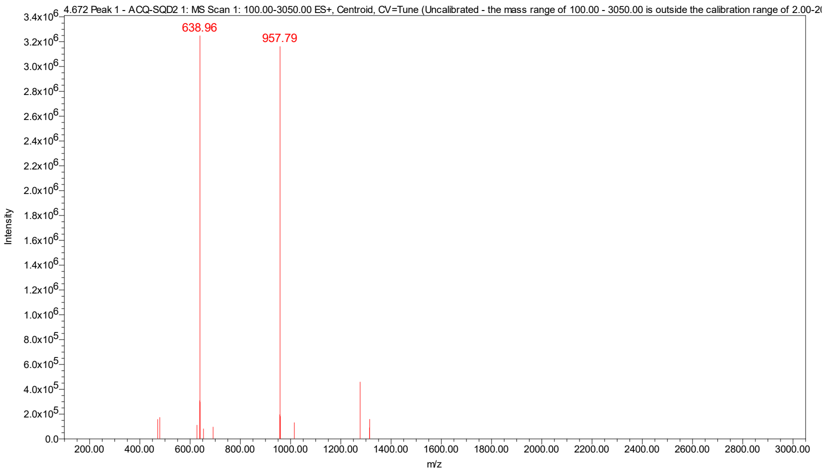

Calculated $[M+3H]^{3+}$:636.57; observed 638.96
Calculated $[M+2H]^{2+}$: 957.35; observed 957.79

BCP19^NF

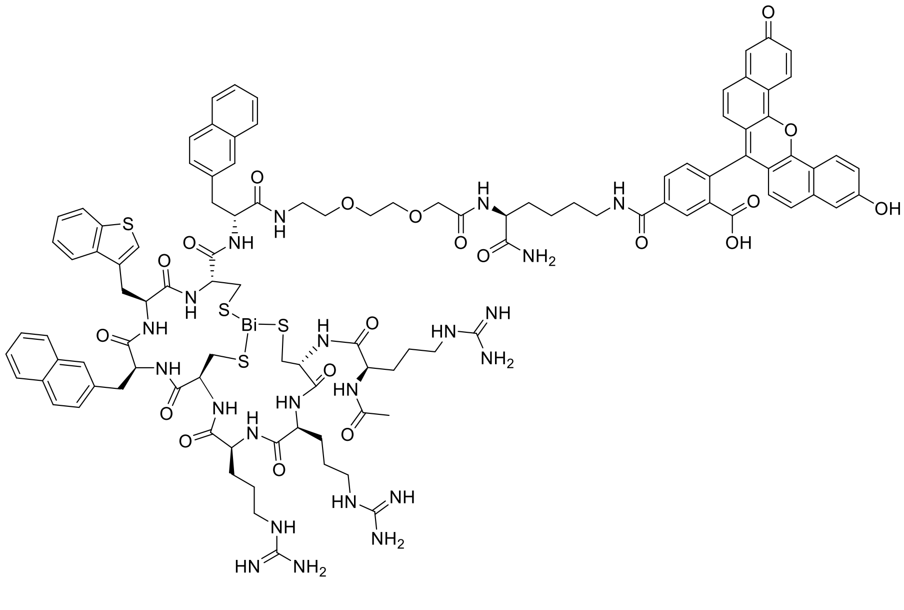

Purity assessment by UPLC (214 nm):

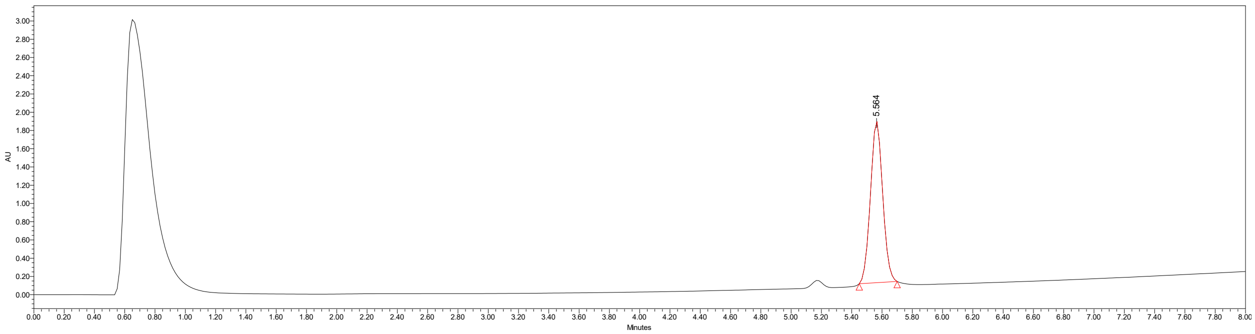

ESI-MS:

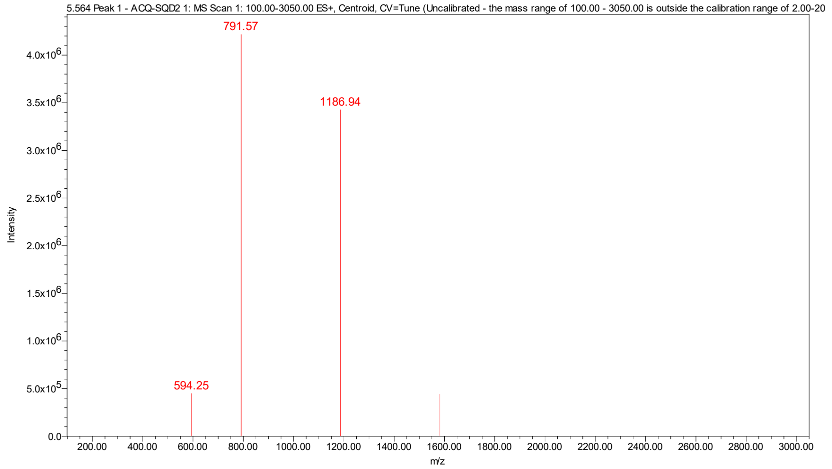

Calculated $[M+4H]^{4+}$:593.69; observed 594.25

Calculated $[M+3H]^{3+}$: 791.26; observed 791.57

Calculated $[M+2H]^{2+}$: 1186.39; observed 1186.94

BCP20

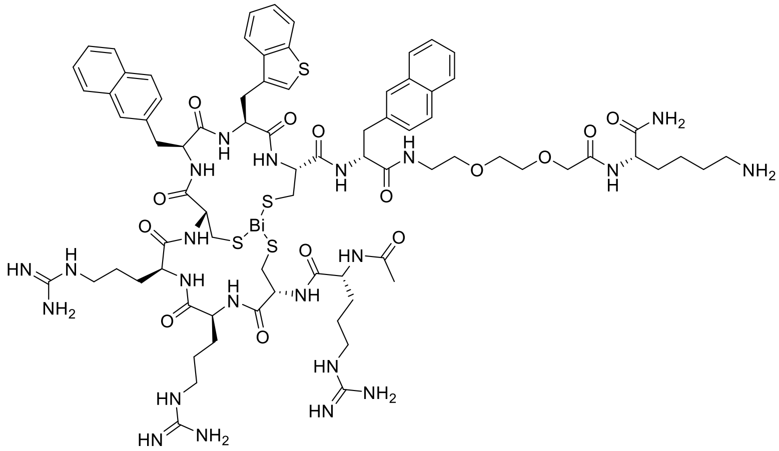

Purity assessment by UPLC (214 nm):

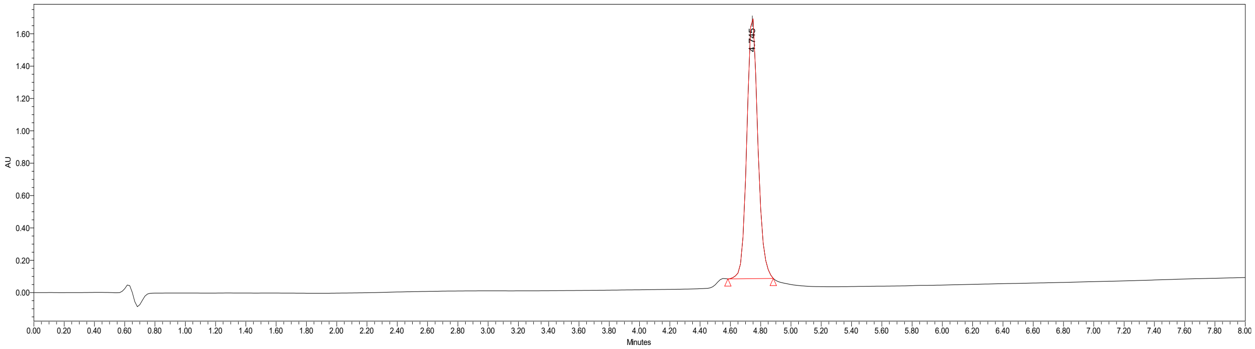

ESI-MS:

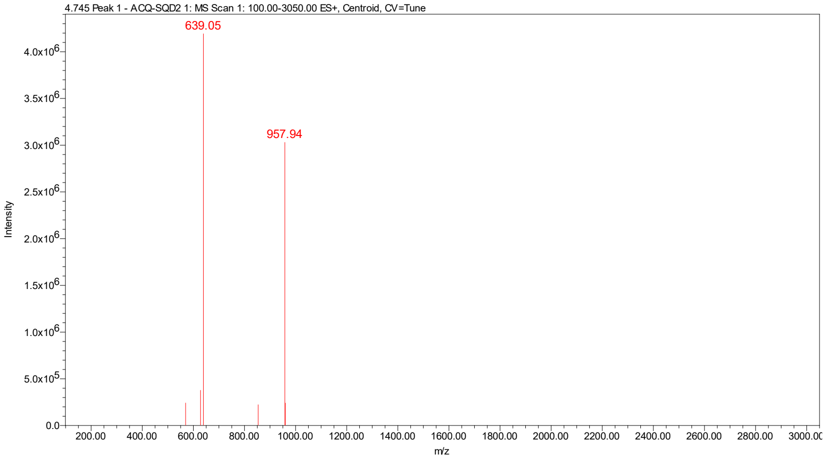

Calculated $[M+3H]^{3+}$: 638.57; observed 639.05
Calculated $[M+2H]^{2+}$: 957.35; observed 957.94

BCP20^{NF}

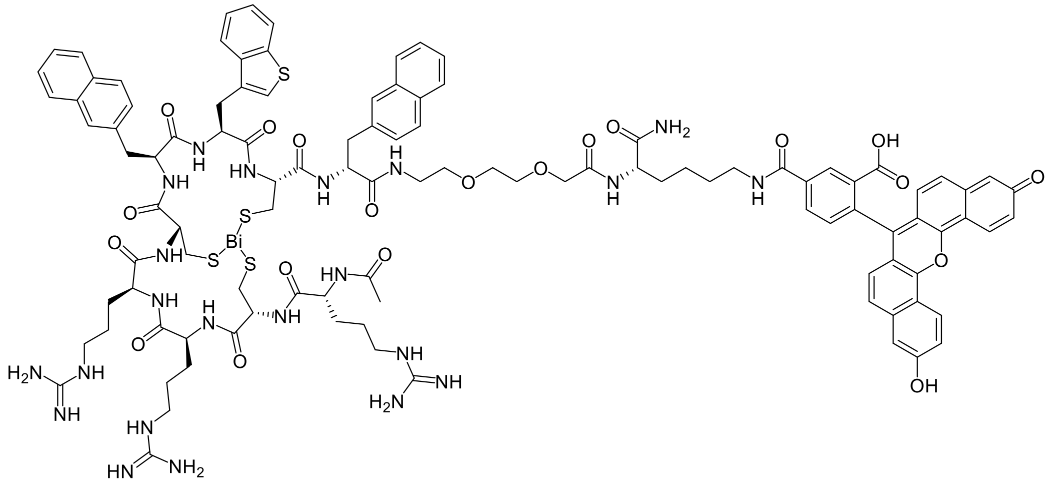

Purity assessment by UPLC (214 nm):

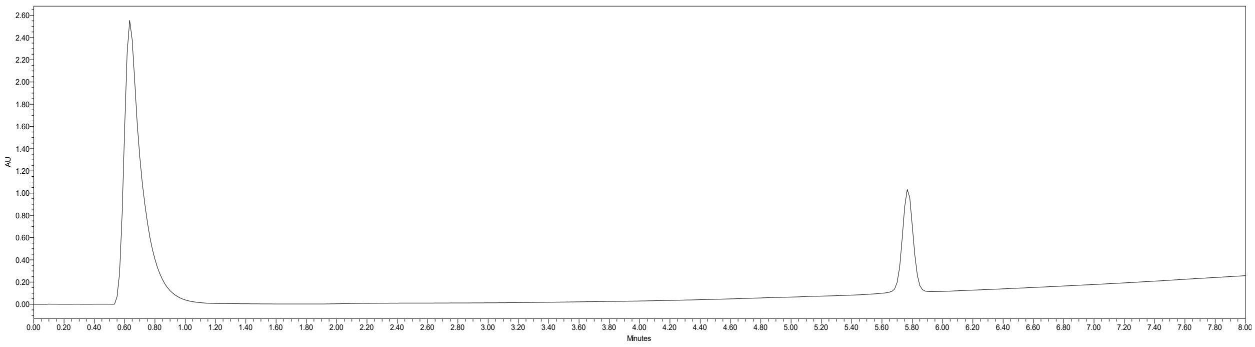

ESI-MS:

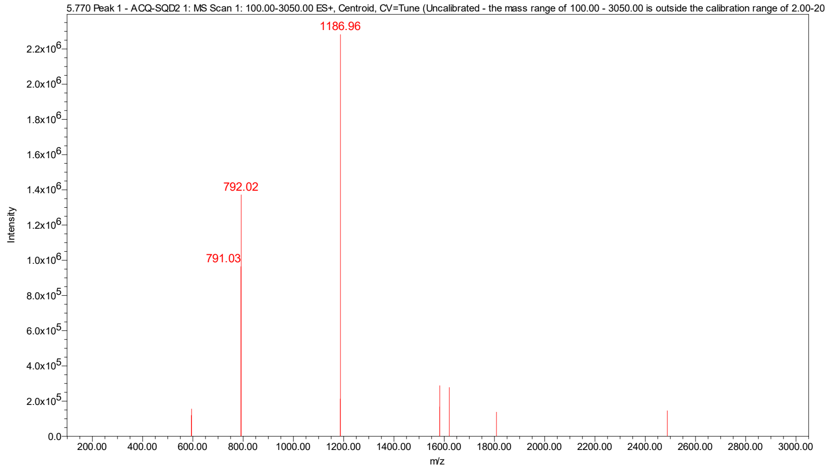

Calculated $[M+3H]^{3+}$: 791.26; observed 792.02
Calculated $[M+2H]^{2+}$: 1186.39; observed 1186.96

BCP16

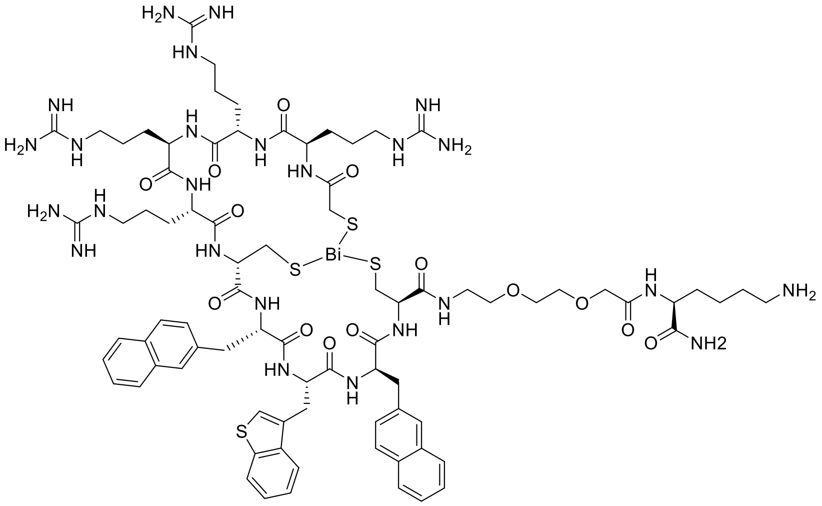

Purity assessment by UPLC (214 nm):

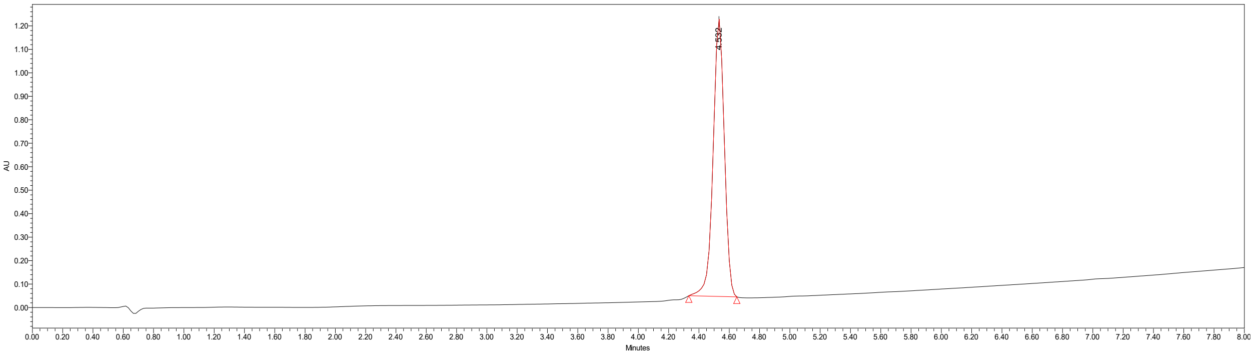

ESI-MS:

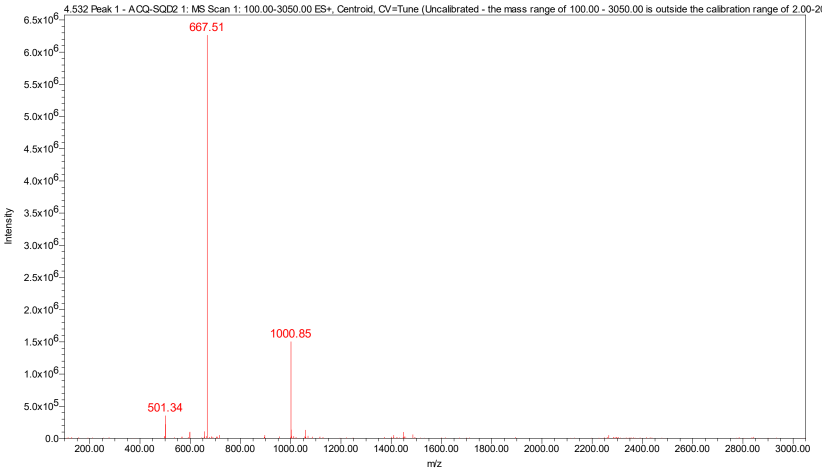

Calculated $[M+4H]^{4+}$:500.44; observed 501.34
Calculated $[M+3H]^{3+}$: 666.92; observed 667.51
Calculated $[M+2H]^{2+}$: 999.89; observed 1000.85

MALDI-TOF-MS:

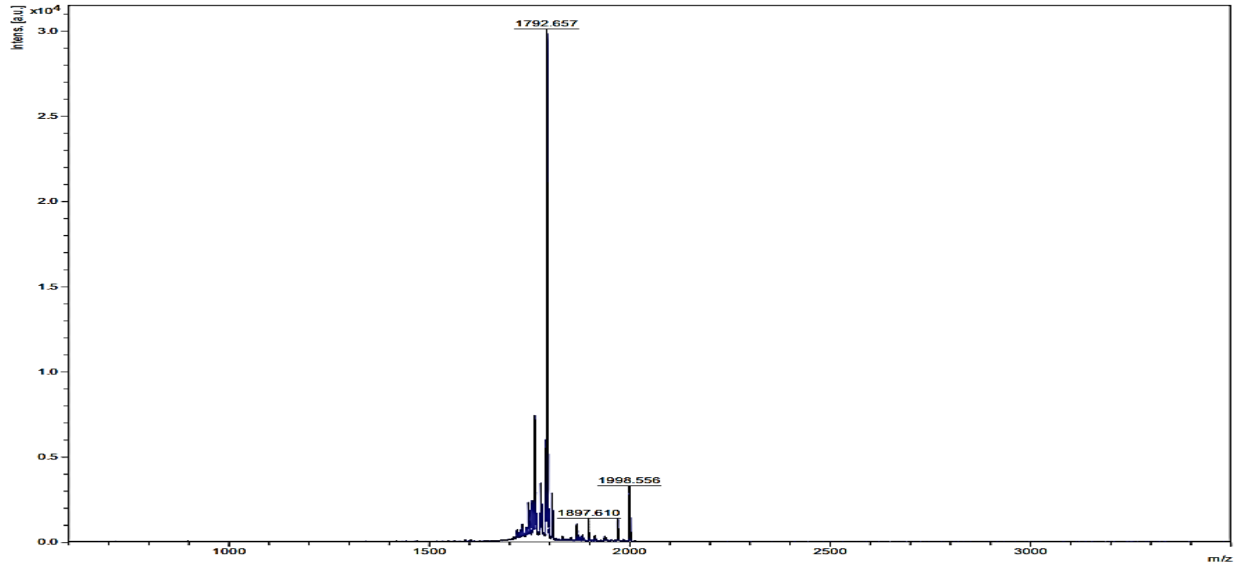

Calculated $[M+H]^+$ (Bismuth bound): 1998.77; observed 1998.556
Calculated $[M+H]^+$ (Bismuth lost): 1792.81; observed 1792.657

BCP16^{NF}

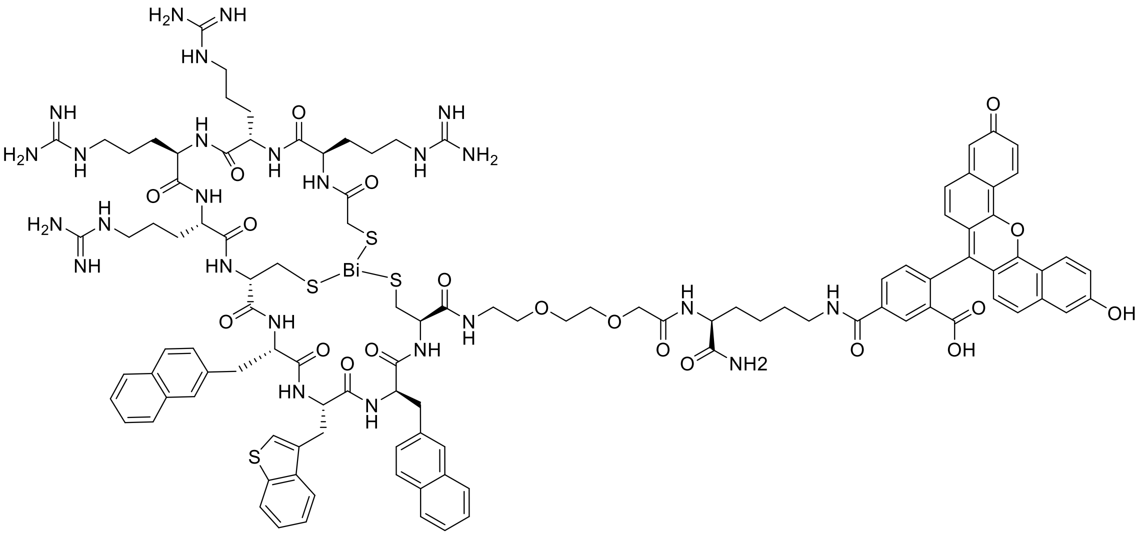

Purity assessment by UPLC (214 nm):

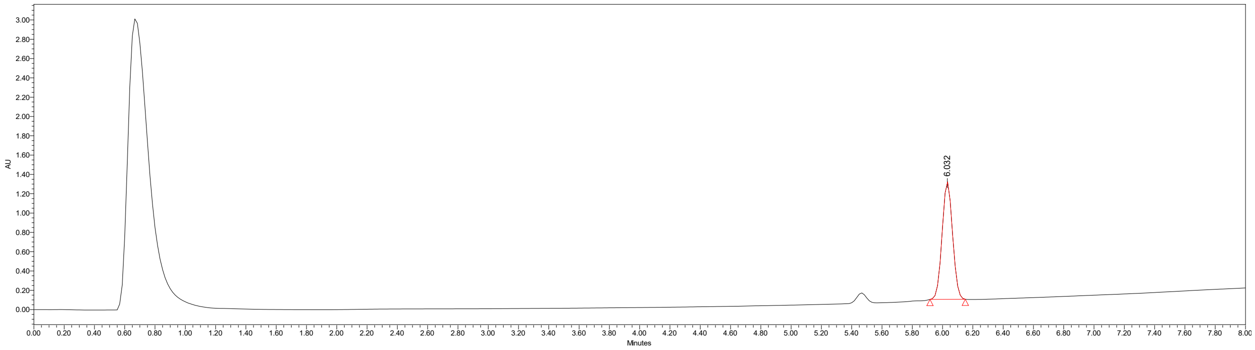

ESI-MS:

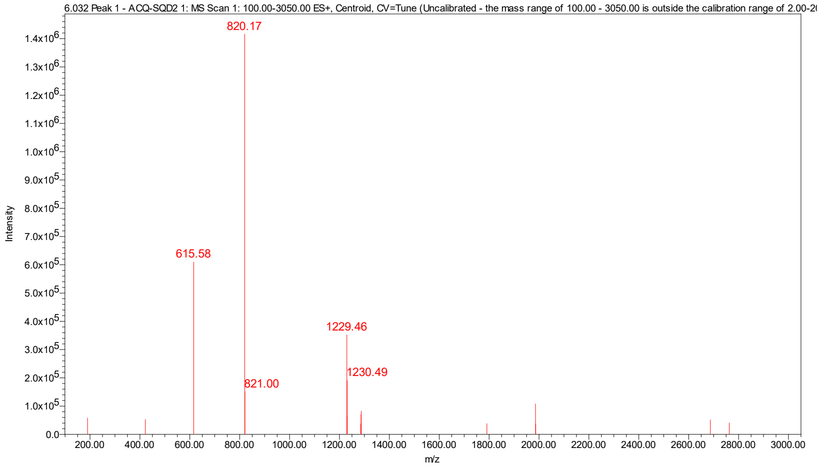

Calculated $[M+4H]^{4+}$:614.96; observed 615.58

Calculated $[M+3H]^{3+}$: 819.62; observed 820.17

Calculated $[M+2H]^{2+}$: 1228.93; observed 1229.46

BCP16^{TMR}

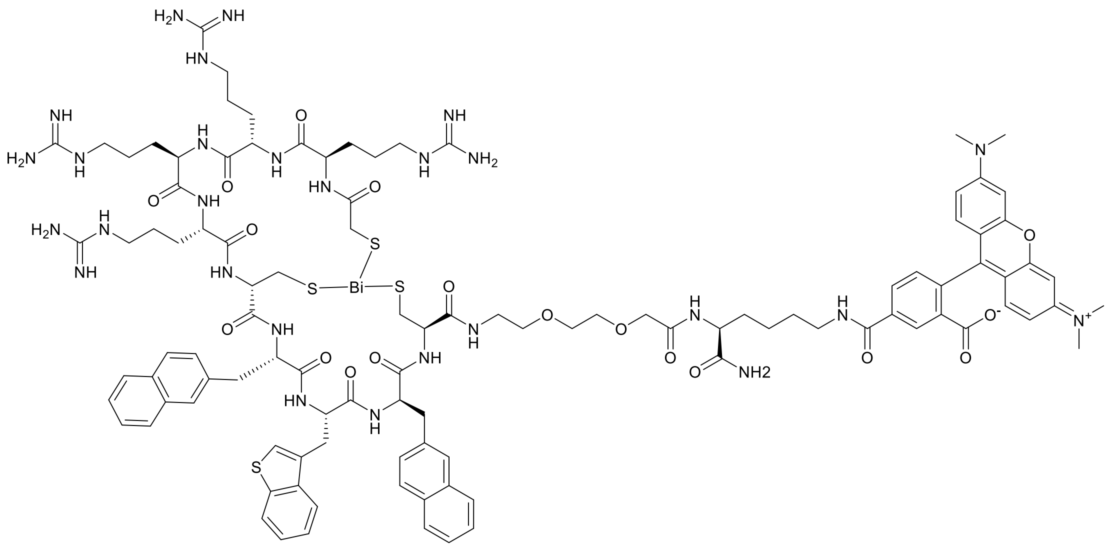

Purity assessment by UPLC (214 nm):

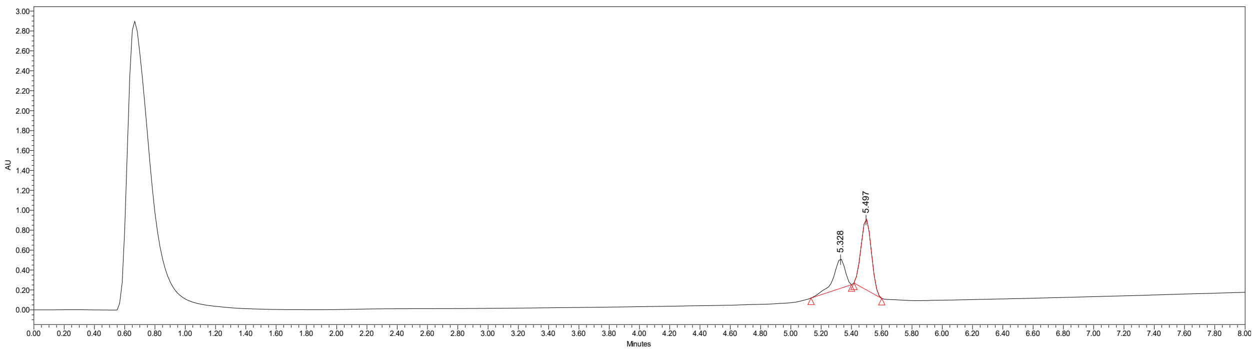

ESI-MS:

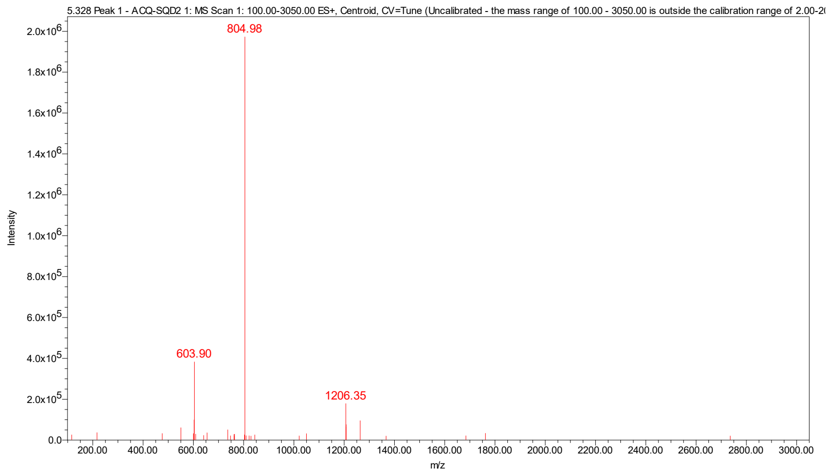

Calculated $[M+4H]^{4+}$:603.47; observed 603.90

Calculated $[M+3H]^{3+}$: 804.30; observed 804.98

Calculated $[M+2H]^{2+}$: 1205.95; observed 1206.35

BCP16a

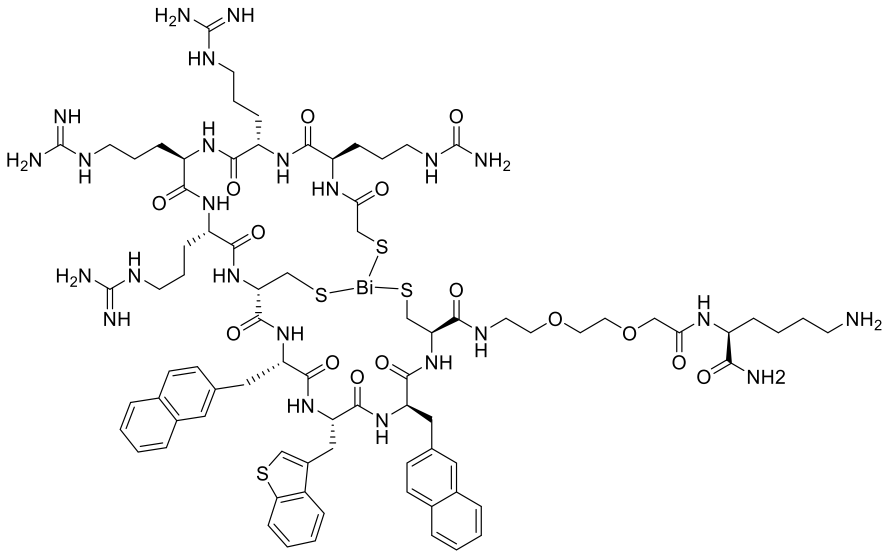

Purity assessment by UPLC (214 nm):

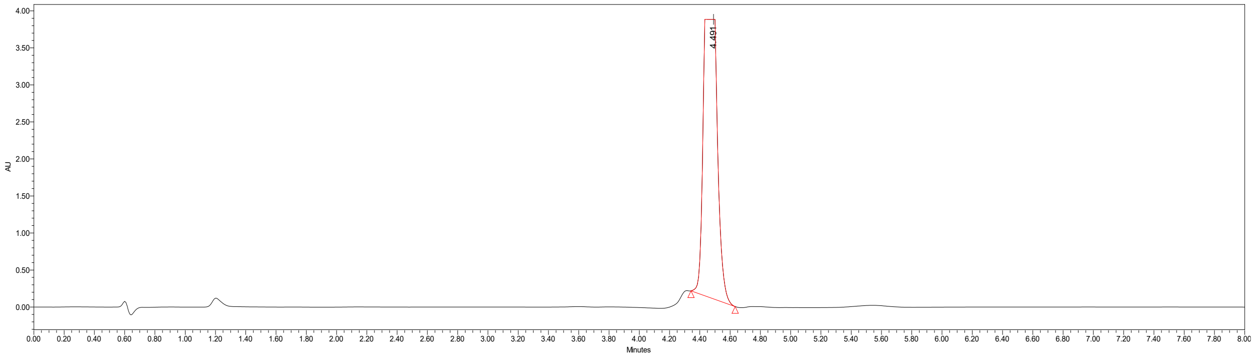

ESI-MS:

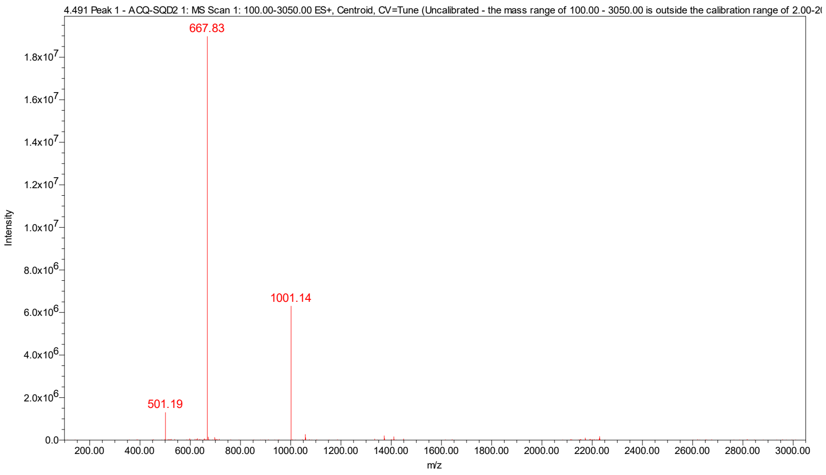

Calculated $[M+4H]^{4+}$:500.68; observed 501.19
Calculated $[M+3H]^{3+}$: 667.25; observed 667.83
Calculated $[M+2H]^{2+}$: 1000.38; observed 1001.14

BCP16a^{NF}

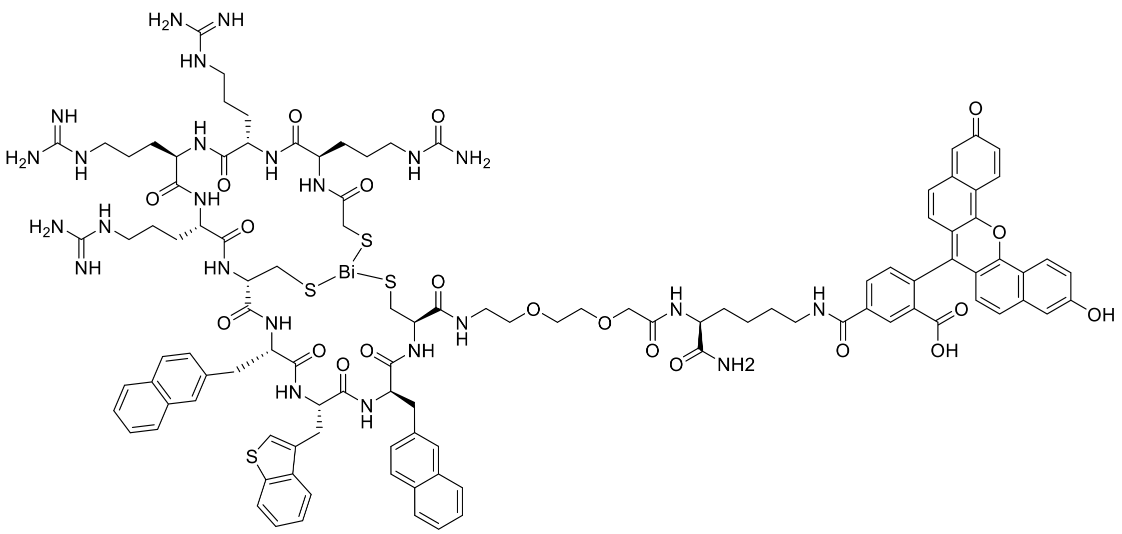

Purity assessment by UPLC (214 nm):

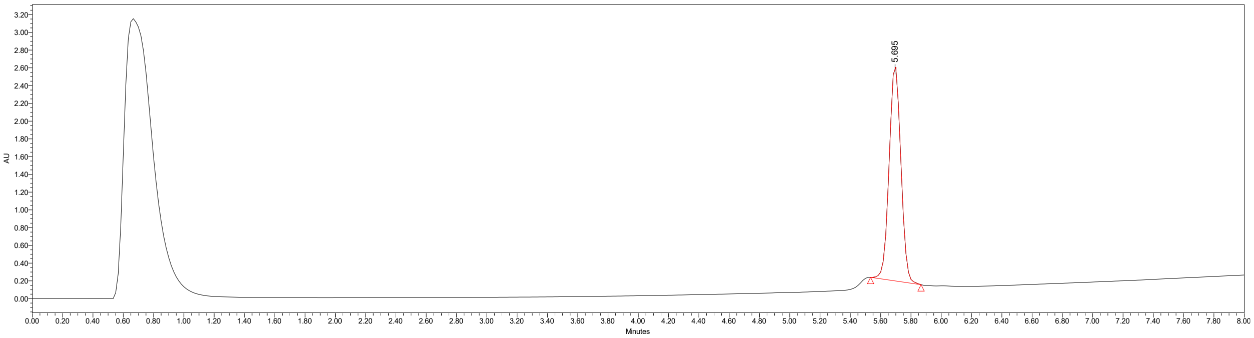

ESI-MS:

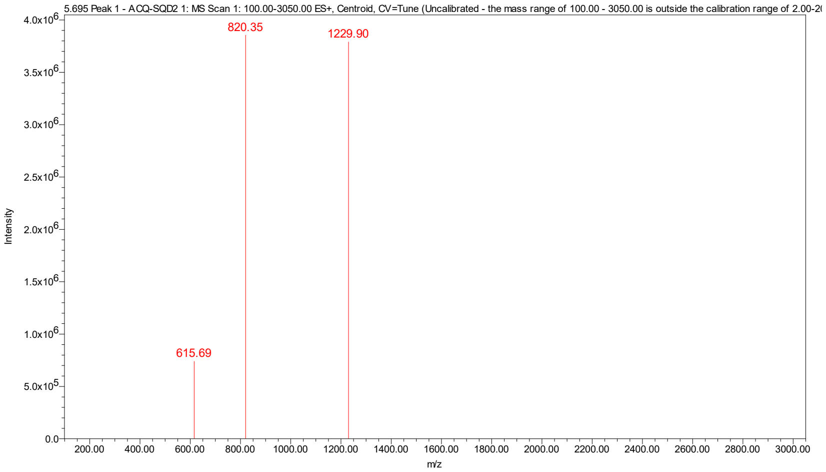

Calculated $[M+4H]^{4+}$:615.21; observed 615.69
Calculated $[M+3H]^{3+}$: 819.94; observed 820.35
Calculated $[M+2H]^{2+}$: 1229.42; observed 1229.90

BCP16b

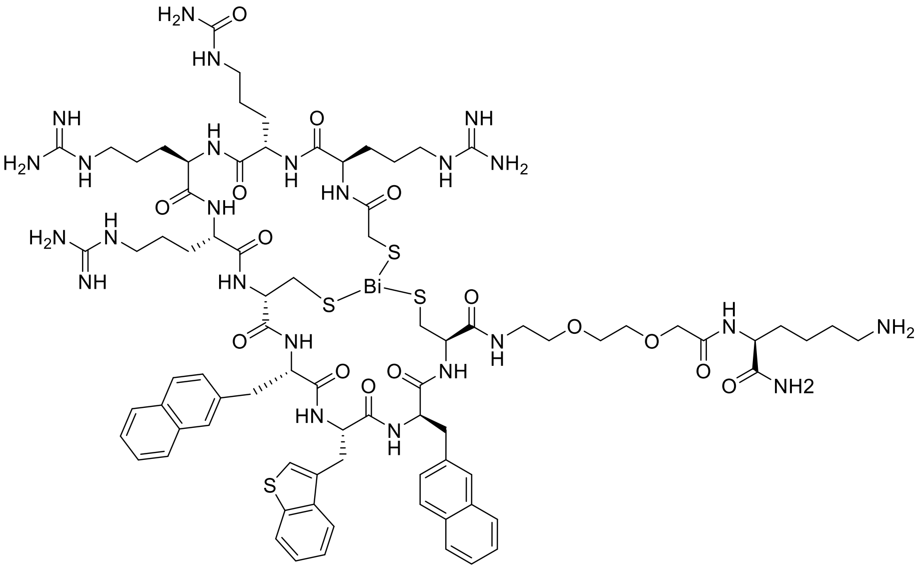

Purity assessment by UPLC (214 nm):

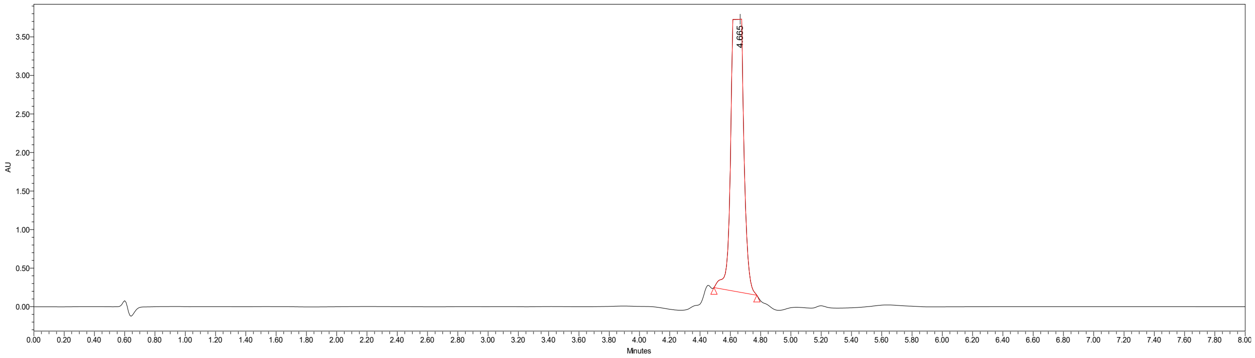

ESI-MS:

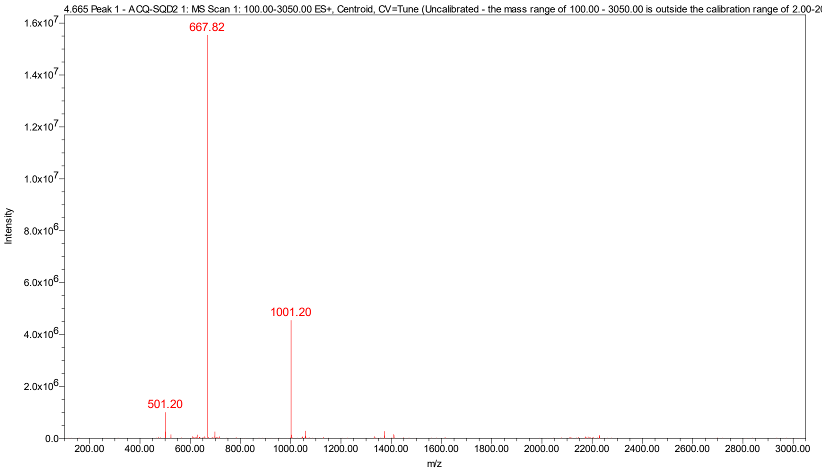

Calculated $[M+4H]^{4+}$:500.68; observed 501.20
Calculated $[M+3H]^{3+}$: 667.25; observed 667.82
Calculated $[M+2H]^{2+}$: 1000.38; observed 1001.20

BCP16b^NF

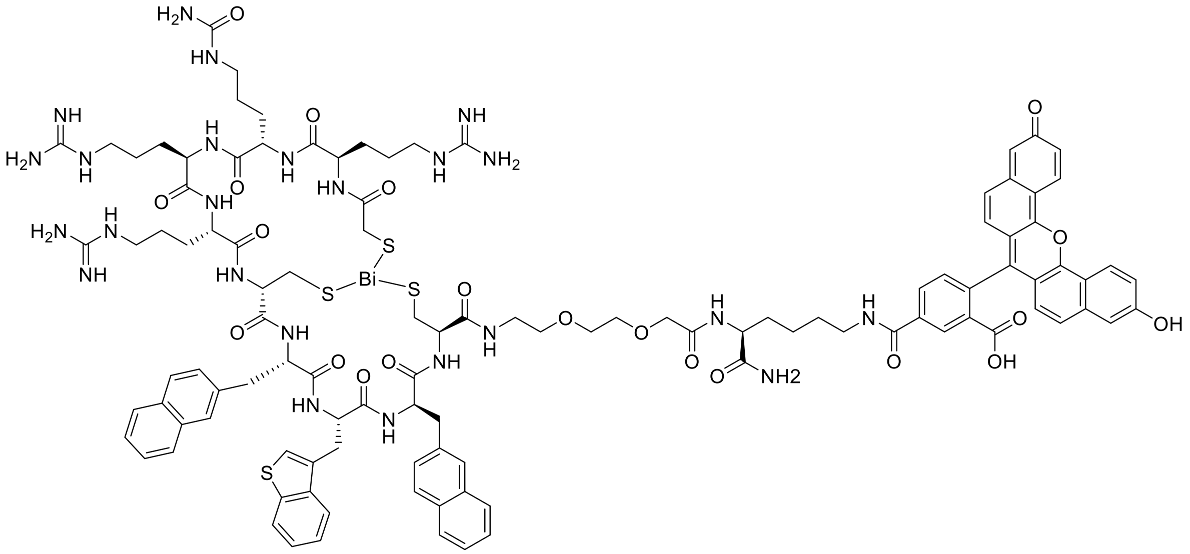

Purity assessment by UPLC (214 nm):

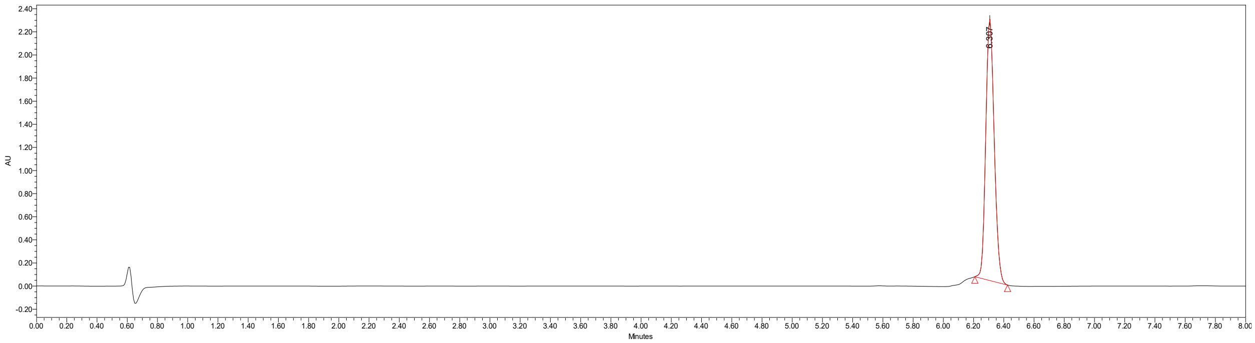

ESI-MS:

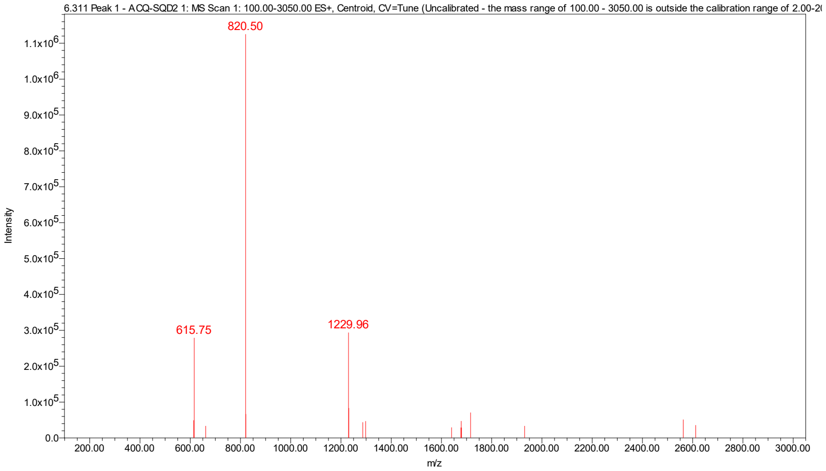

Calculated $[M+4H]^{4+}$:615.21; observed 615.75

Calculated $[M+3H]^{3+}$: 819.94; observed 820.50

Calculated $[M+2H]^{2+}$: 1229.42; observed 1229.96

BCP16c

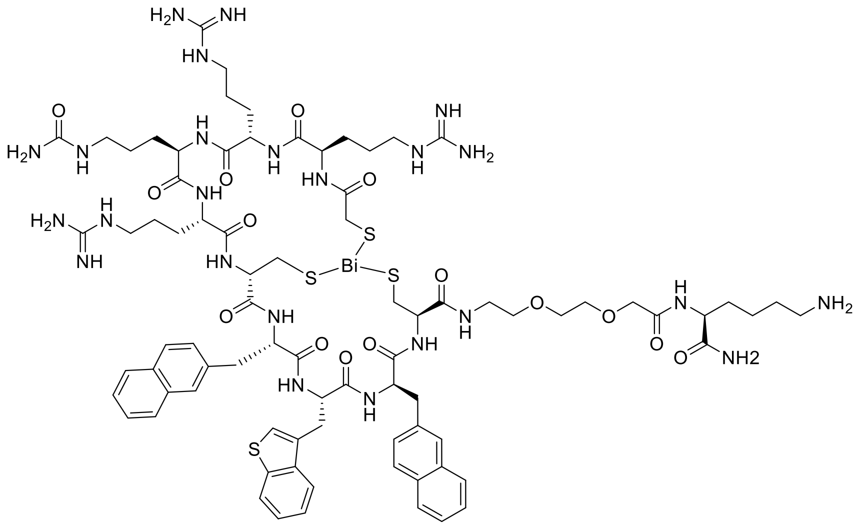

Purity assessment by UPLC (214 nm):

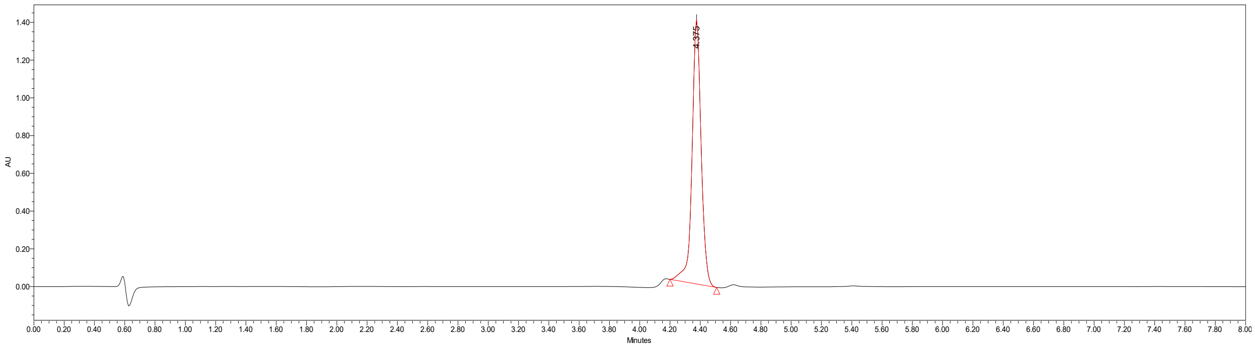

ESI-MS:

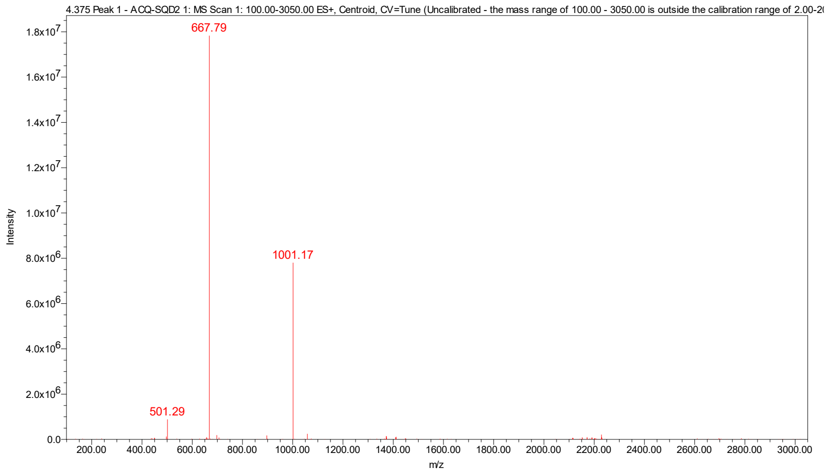

Calculated $[M+4H]^{4+}$:500.68; observed 501.29
Calculated $[M+3H]^{3+}$: 667.25; observed 667.79
Calculated $[M+2H]^{2+}$: 1000.38; observed 1001.17

BCP16c^{NF}

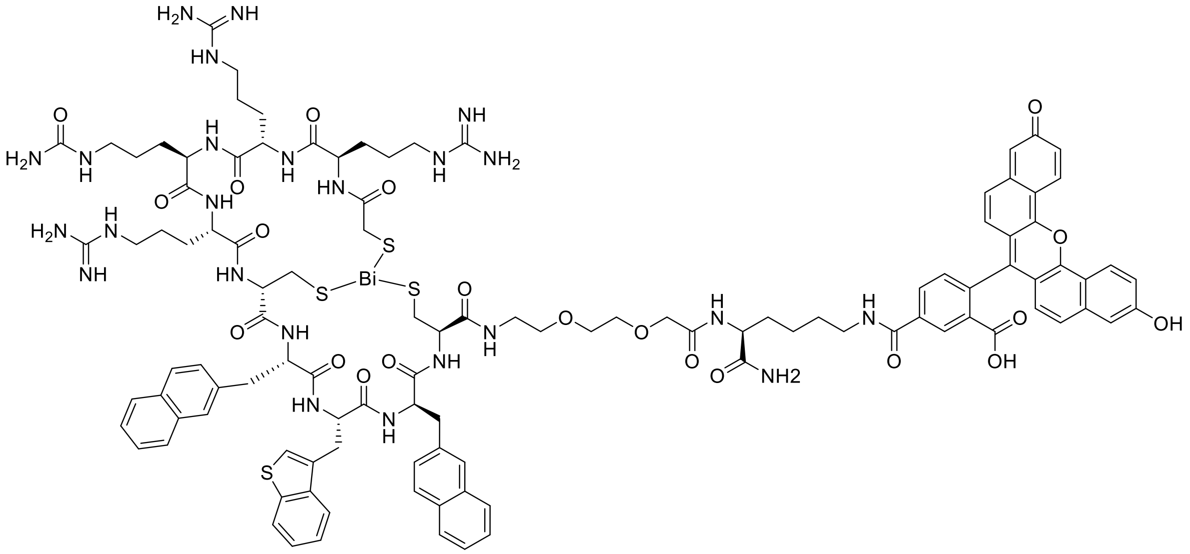

Purity assessment by UPLC (214 nm):

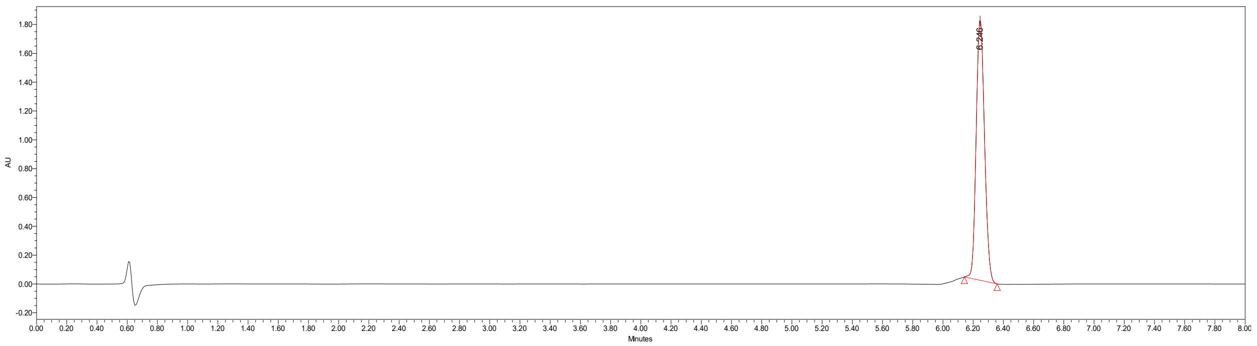

ESI-MS:

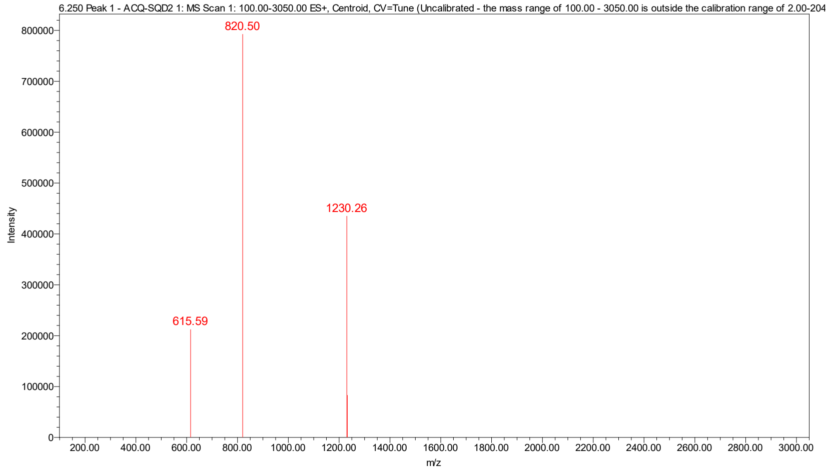

Calculated $[M+4H]^{4+}$:615.21; observed 615.59
Calculated $[M+3H]^{3+}$: 819.94; observed 820.50
Calculated $[M+2H]^{2+}$: 1229.42; observed 1230.26

BCP16d

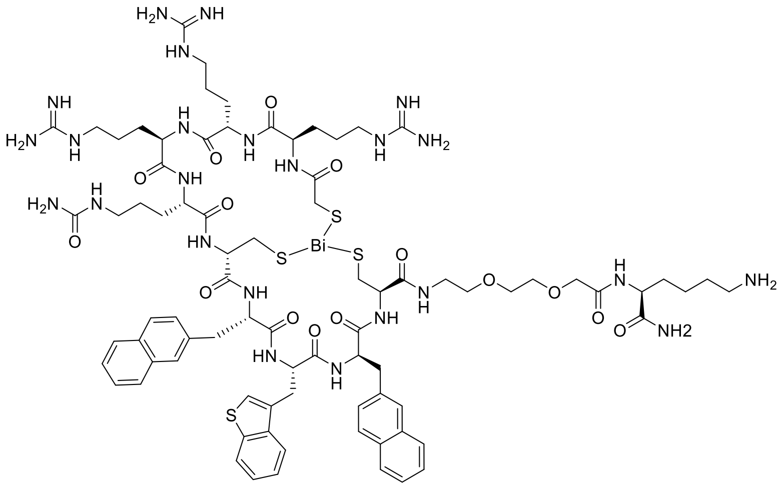

Purity assessment by UPLC (214 nm):

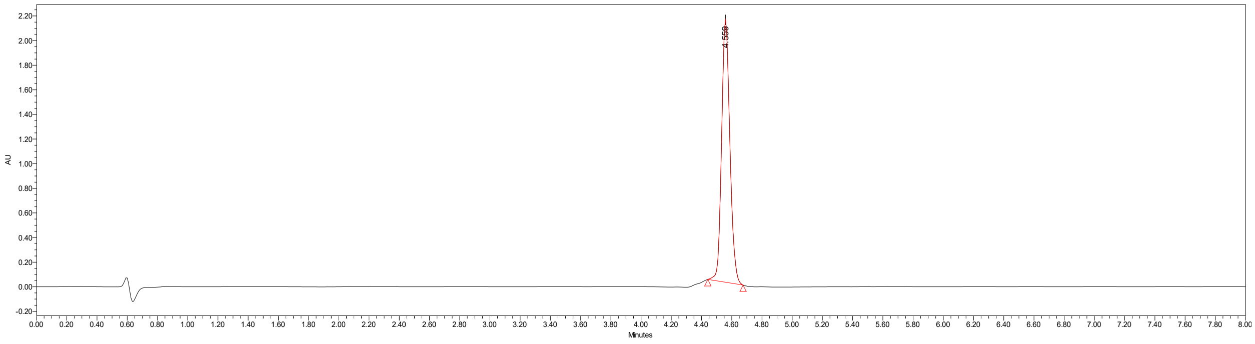

ESI-MS:

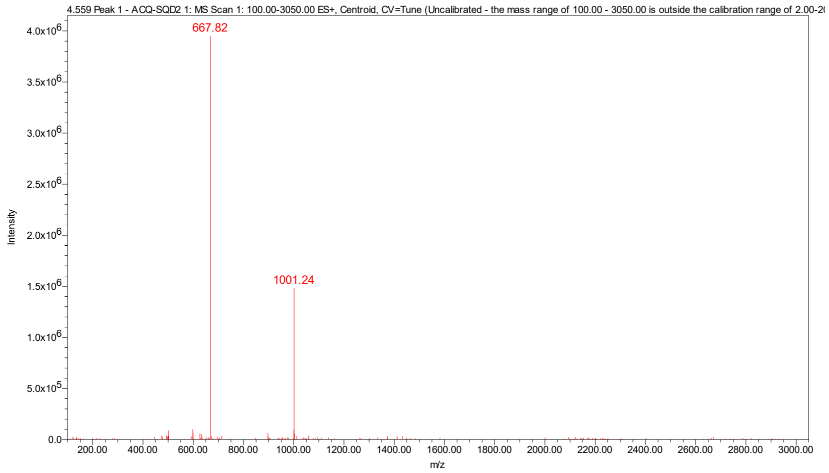

Calculated $[M+3H]^{3+}$: 667.25; observed 667.82
Calculated $[M+2H]^{2+}$: 1000.38; observed 1001.24

BCP16d[NF]

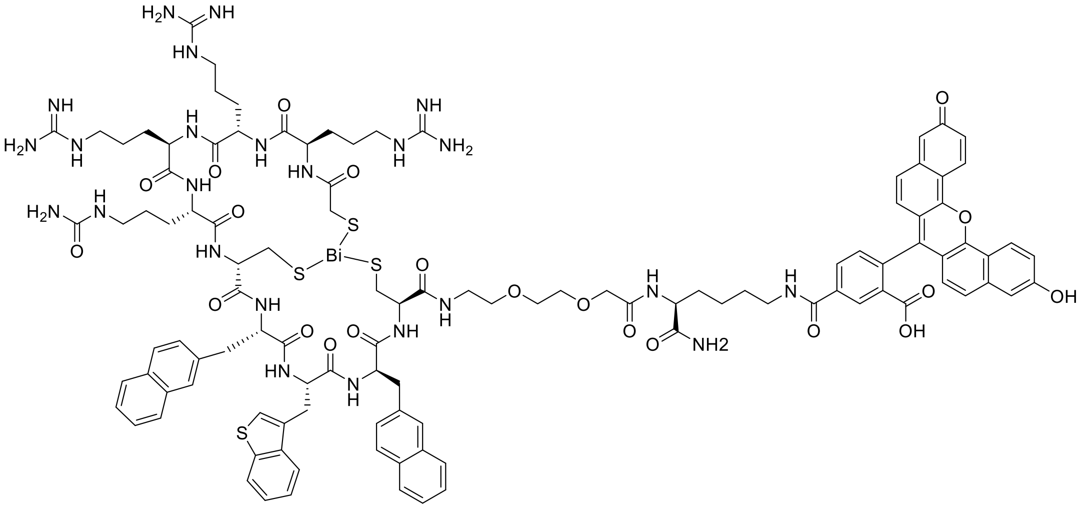

Purity assessment by UPLC (214 nm):

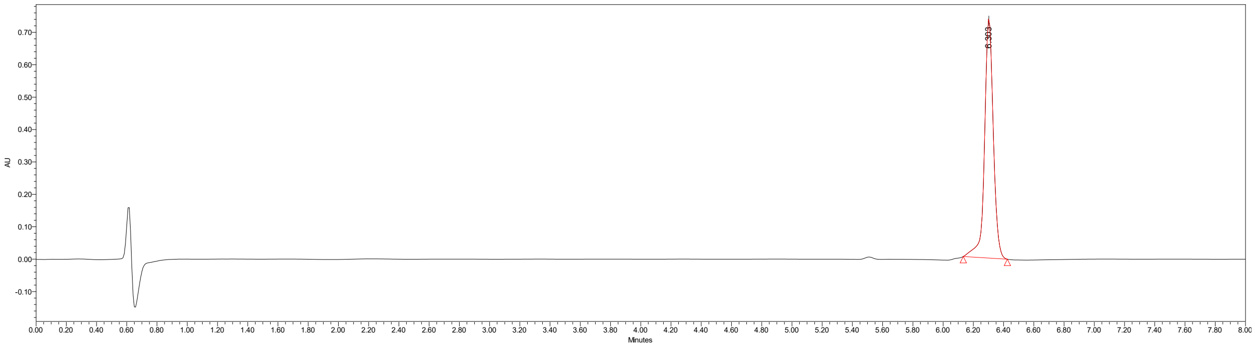

ESI-MS:

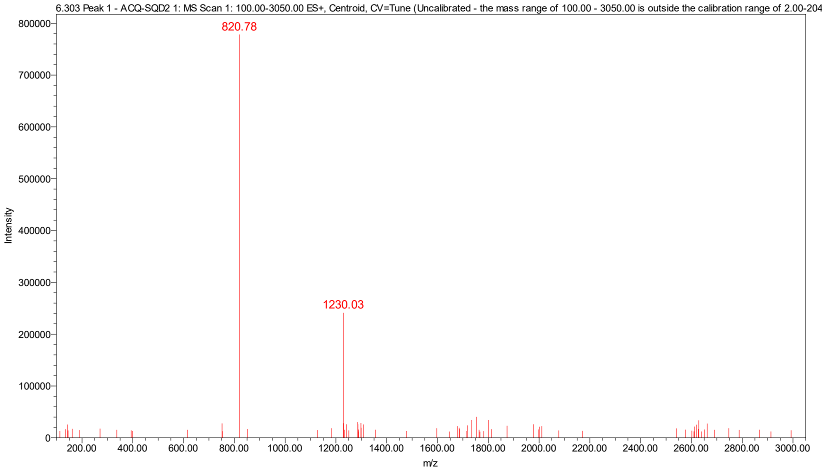

Calculated $[M+3H]^{3+}$: 819.94; observed 820.78

Calculated $[M+2H]^{2+}$: 1229.42; observed 1230.03

BCP16e

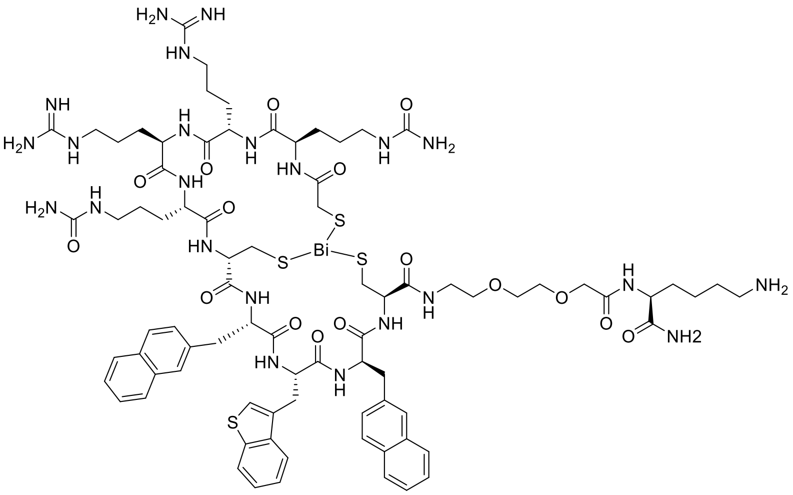

Purity assessment by UPLC (214 nm):

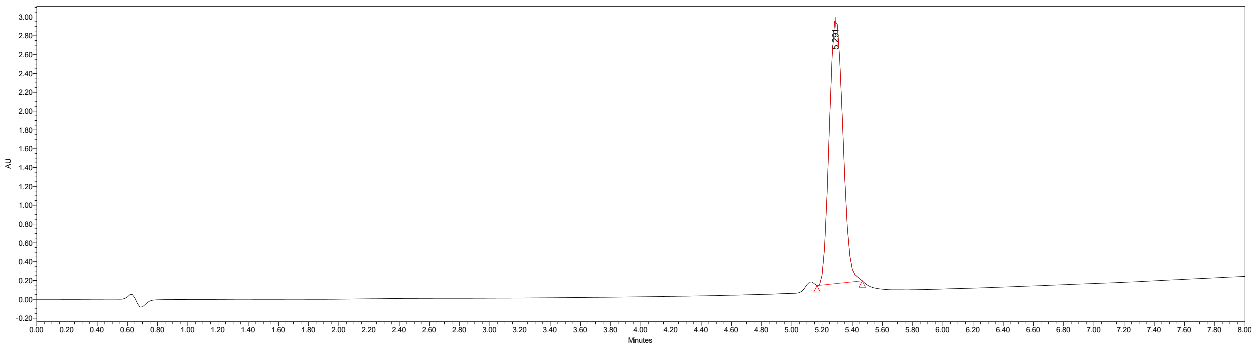

ESI-MS:

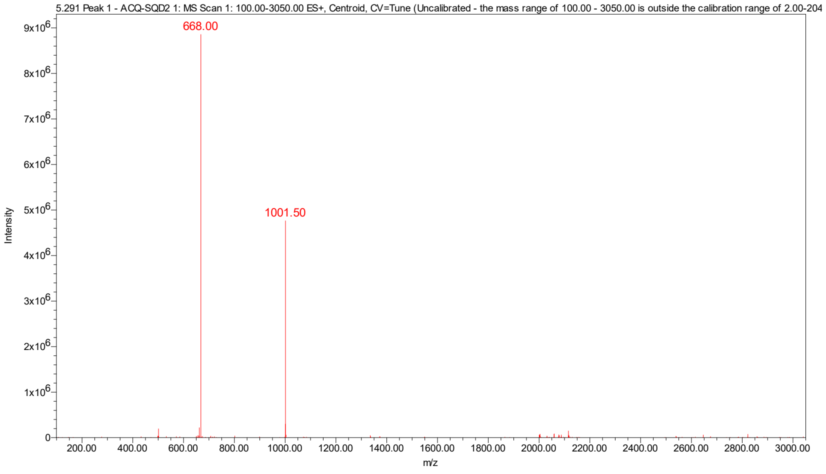

Calculated $[M+3H]^{3+}$: 667.58; observed 668.00
Calculated $[M+2H]^{2+}$: 1000.87; observed 1001.50

MALDI-TOF-MS:

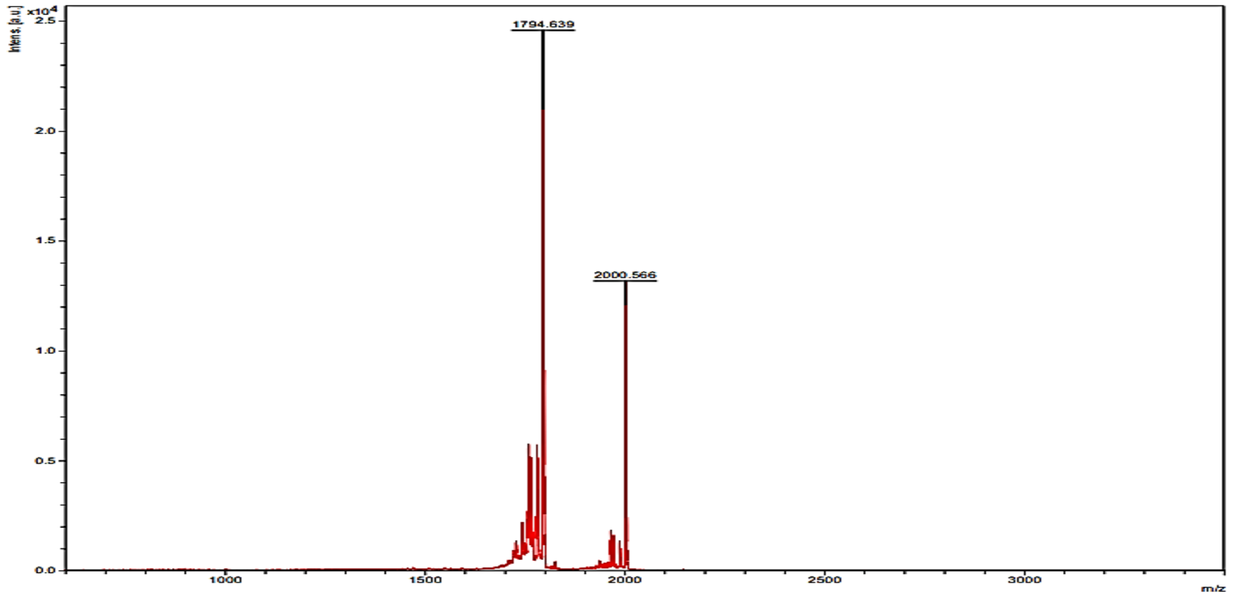

Calculated $[M+H]^+$ (Bismuth bound): 2000.73; observed 2000.566
Calculated $[M+H]^+$ (Bismuth lost): 1794.78; observed 1794.639

BCP16e^NF

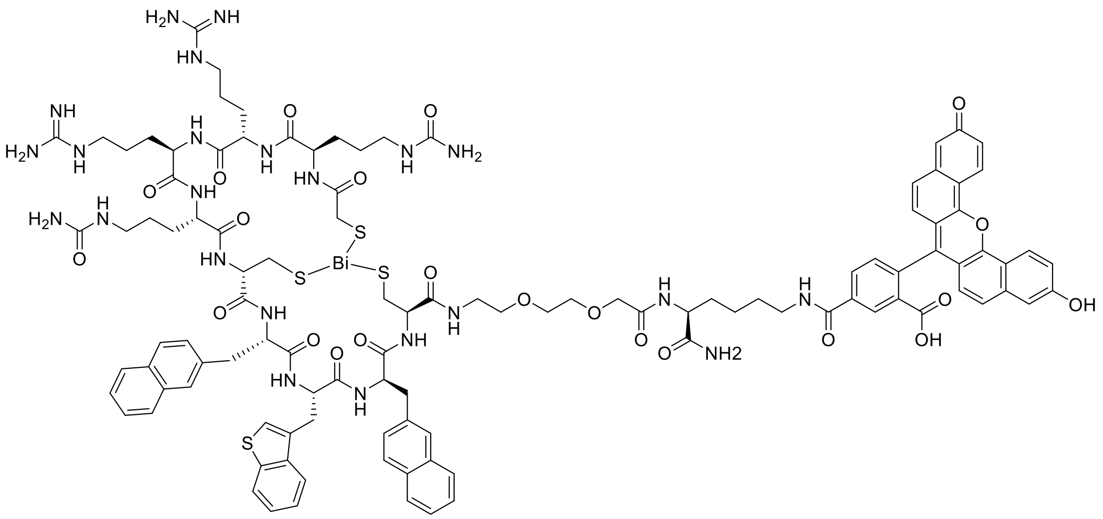

Purity assessment by UPLC (214 nm):

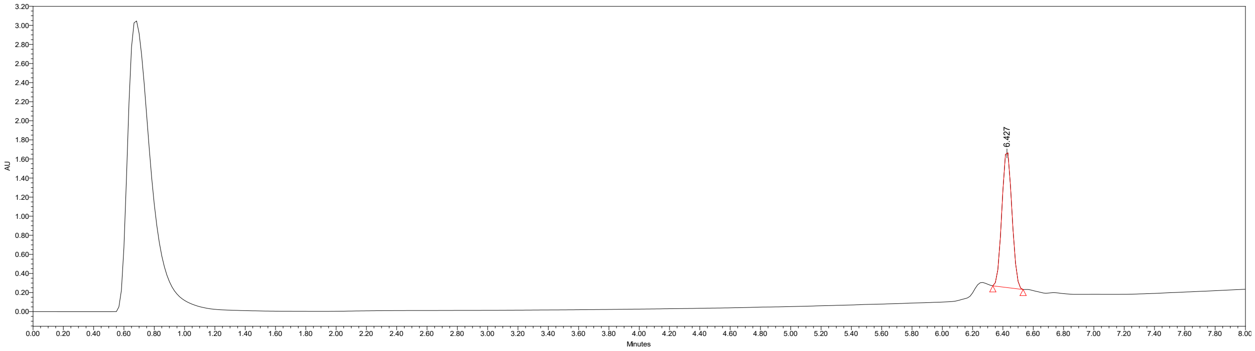

ESI-MS:

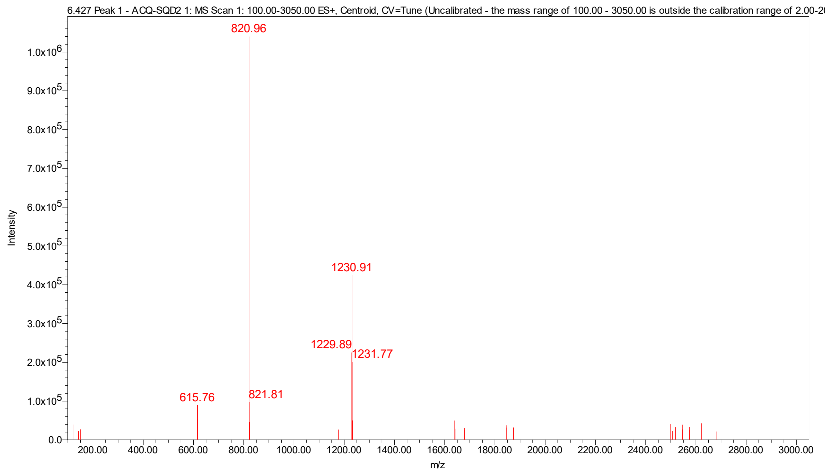

Calculated $[M+4H]^{4+}$:615.45; observed 615.76
Calculated $[M+3H]^{3+}$: 820.27; observed 820.96
Calculated $[M+2H]^{2+}$: 1229.91; observed 1230.91

BCP16e^{TMR}

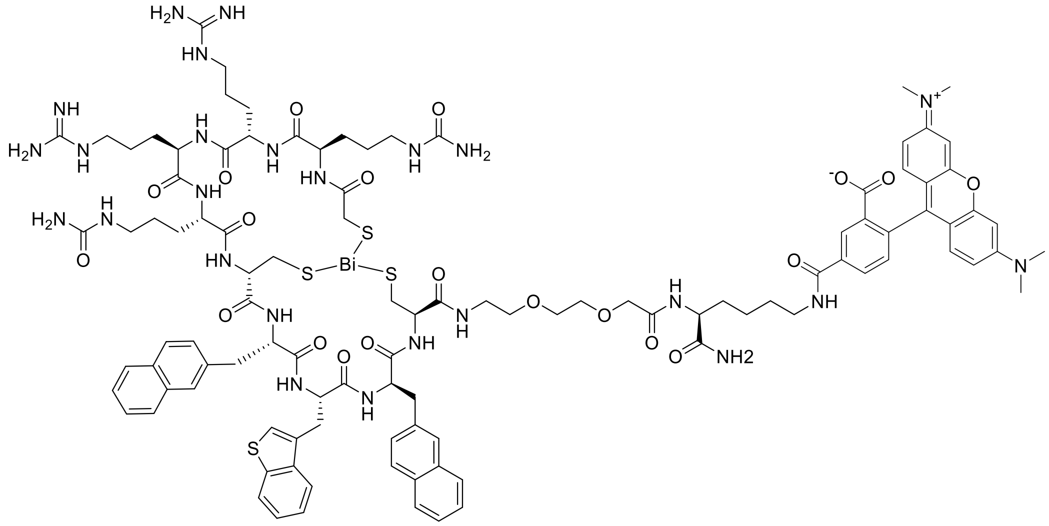

Purity assessment by UPLC (214 nm):

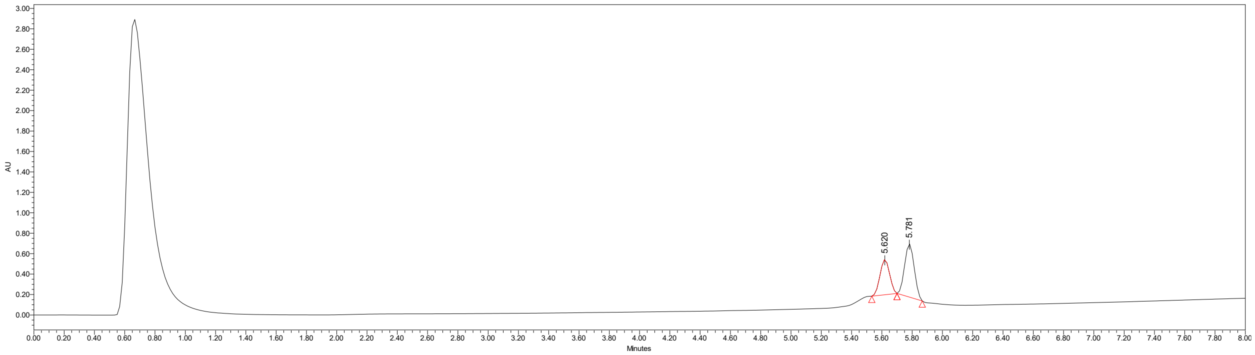

ESI-MS:

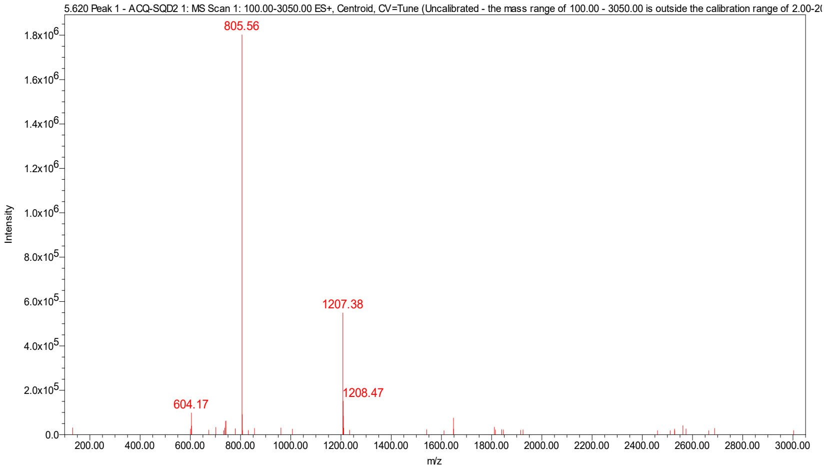

Calculated $[M+4H]^{4+}$:603.97; observed 604.17

Calculated $[M+3H]^{3+}$: 804.96; observed 805.58

Calculated $[M+2H]^{2+}$: 1206.94; observed 1207.38

P1

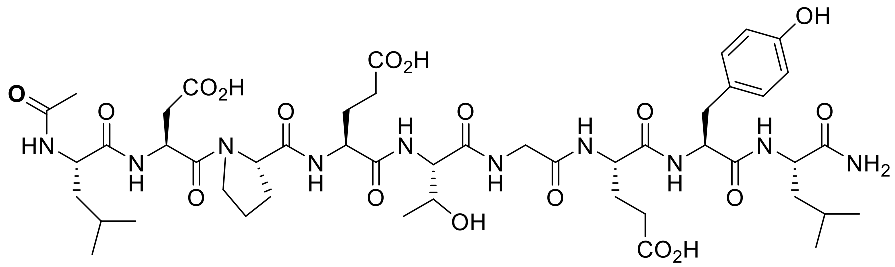

Purity assessment by UPLC (214 nm):

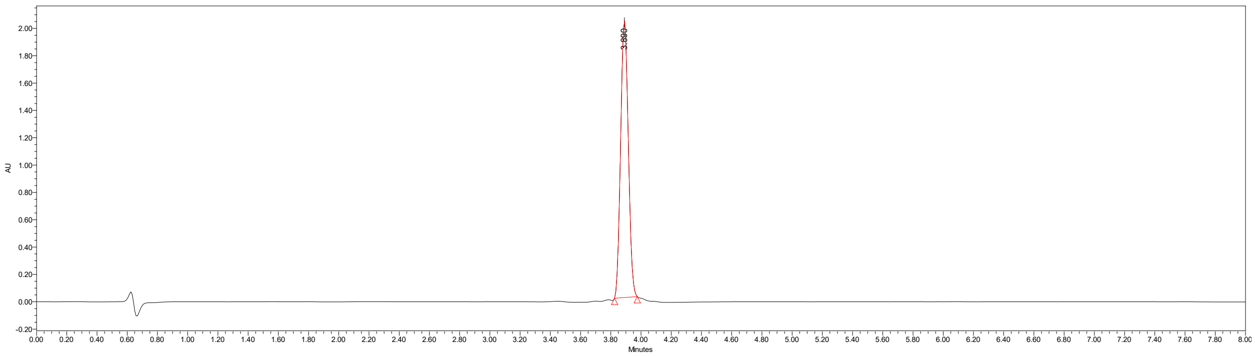

ESI-MS:

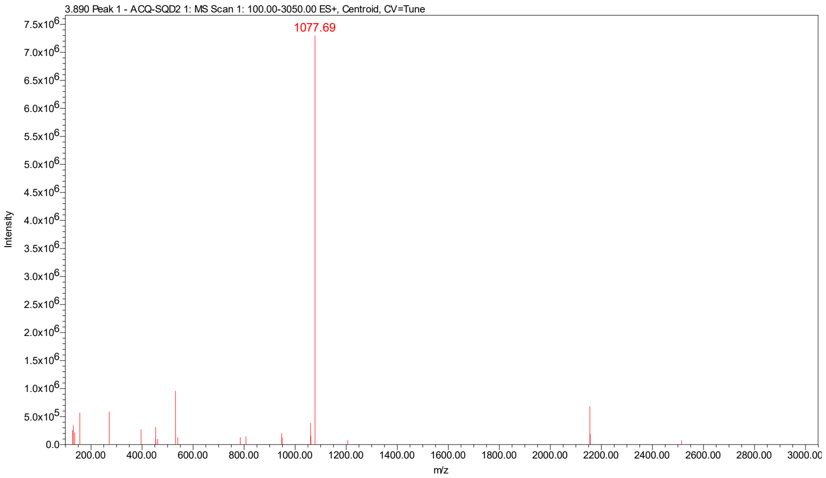

Calculated $[M+1H]^{1+}$: 1076.60; observed 1077.69

CPP12-P1

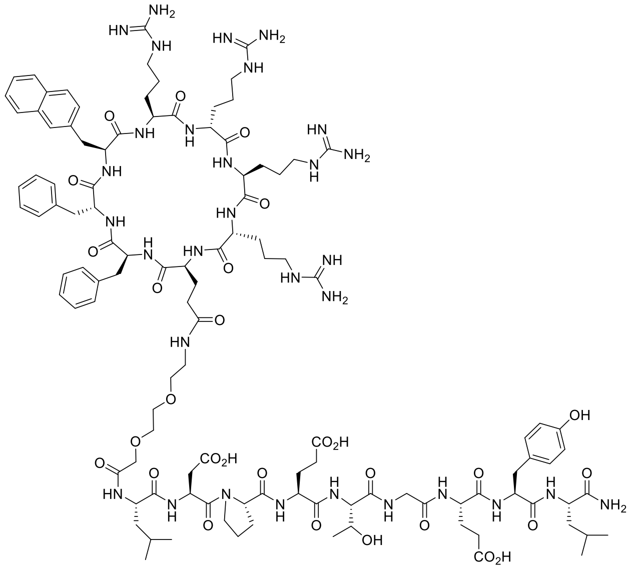

Purity assessment by UPLC (214 nm):

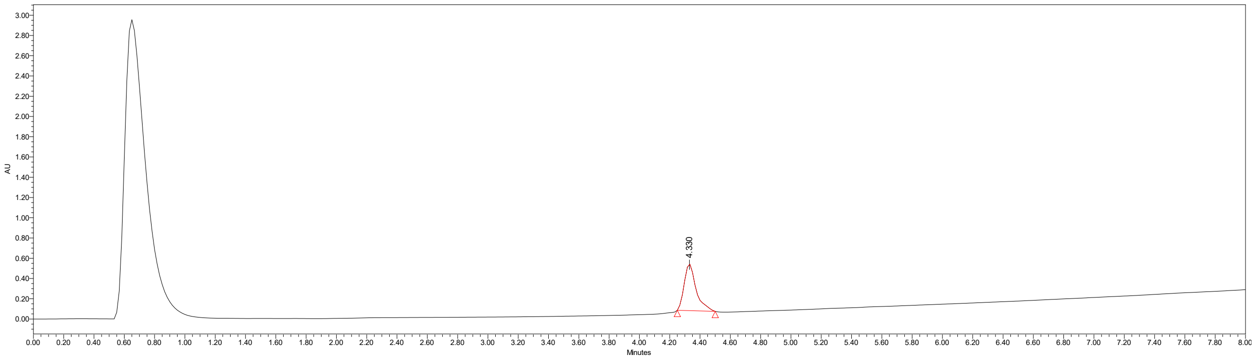

ESI-MS:

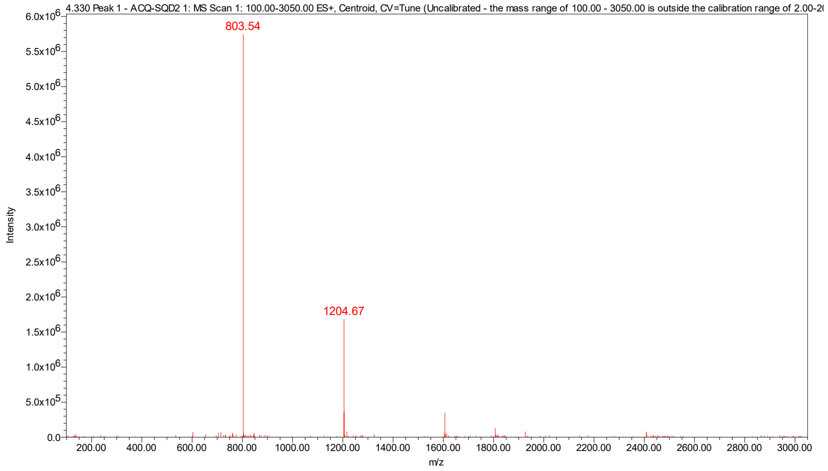

Calculated $[M+3H]^{3+}$: 803.07; observed 803.54
Calculated $[M+2H]^{2+}$: 1204.11; observed 1204.67

BCP16-P1

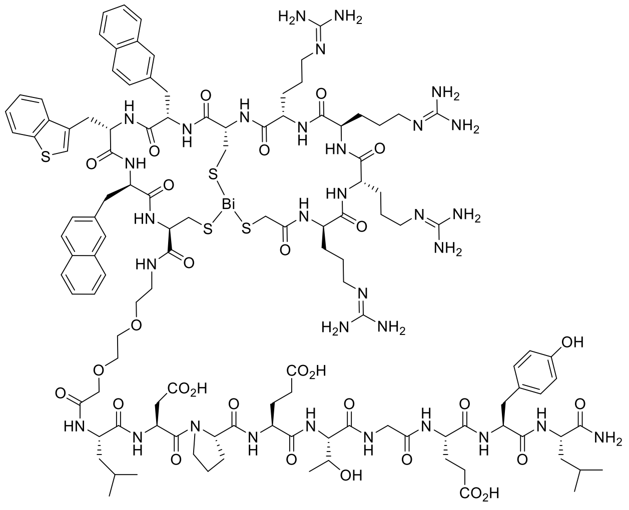

Purity assessment by UPLC (214 nm):

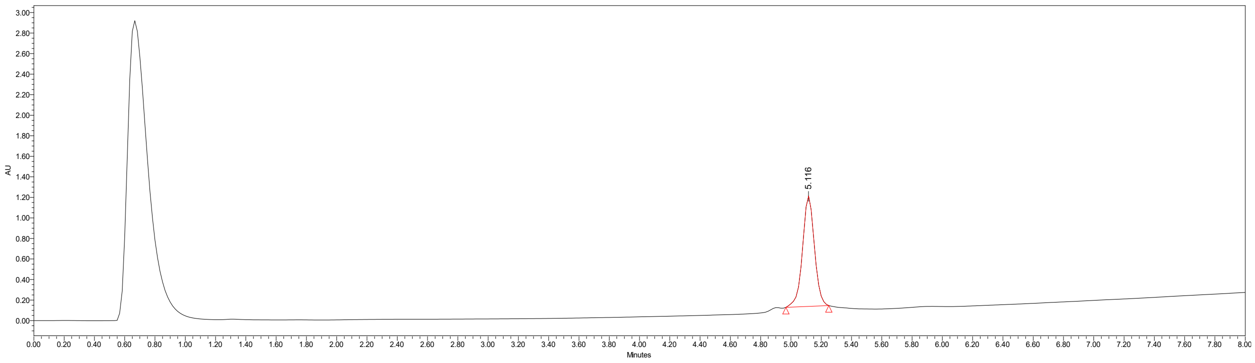

ESI-MS:

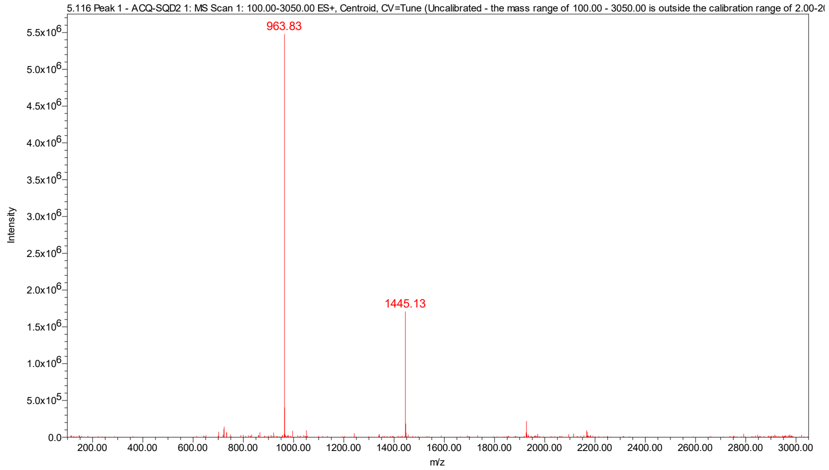

Calculated $[M+3H]^{3+}$: 963.38; observed 963.83
Calculated $[M+2H]^{2+}$: 1444.57; observed 1445.13

BCP16e-P1

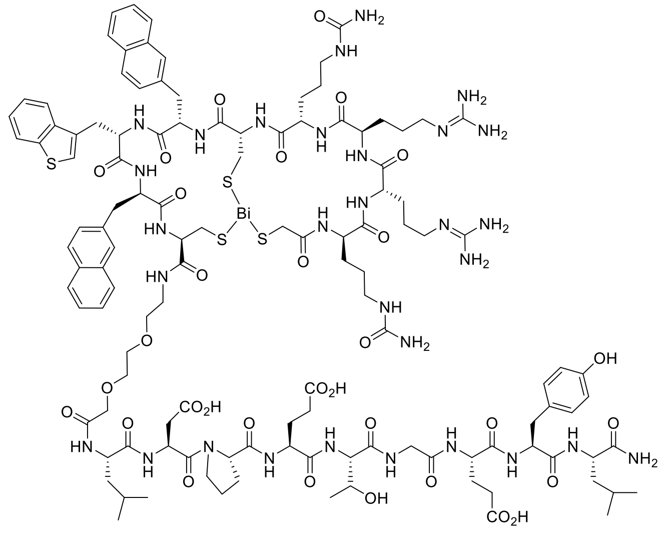

Purity assessment by UPLC (214 nm):

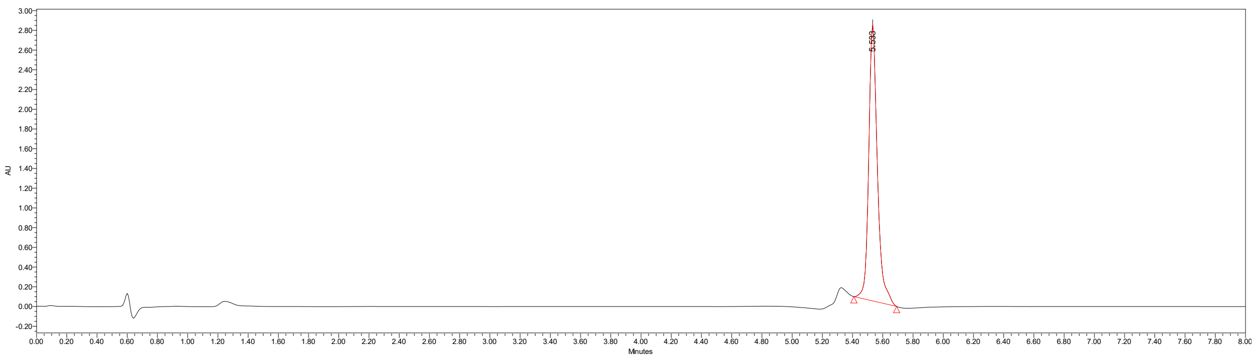

ESI-MS:

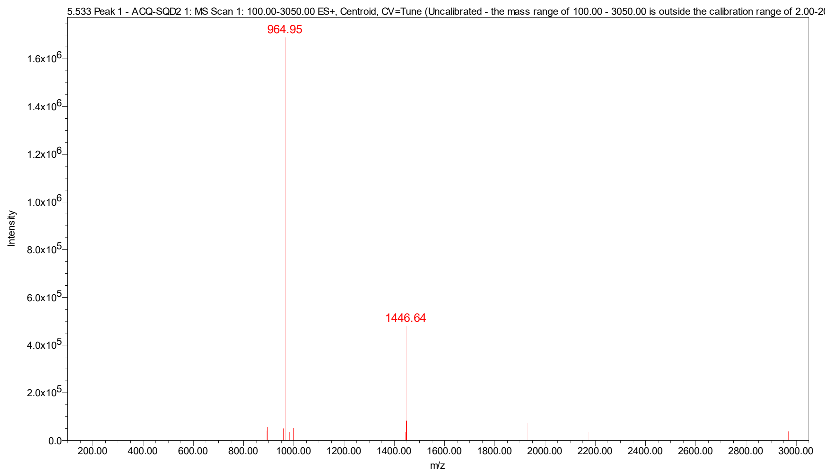

Calculated $[M+3H]^{3+}$: 964.03; observed 964.95

Calculated $[M+2H]^{2+}$: 1445.55; observed 1446.64

BCP16 (for mouse MTD study)

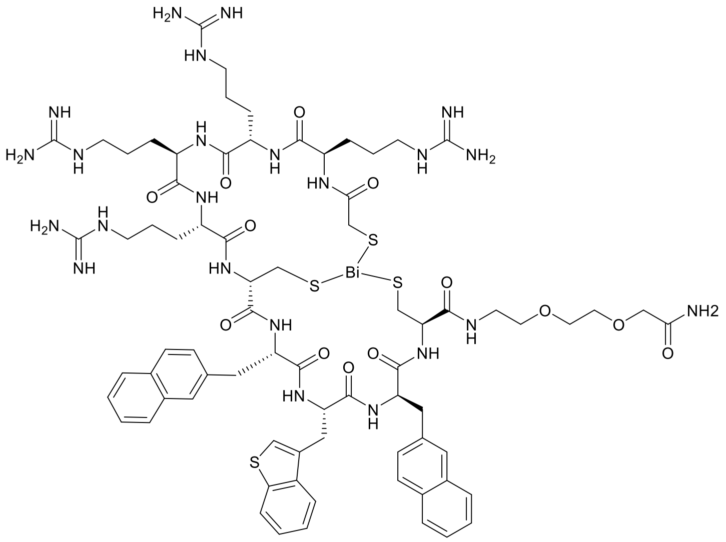

Purity assessment by UPLC (214 nm):

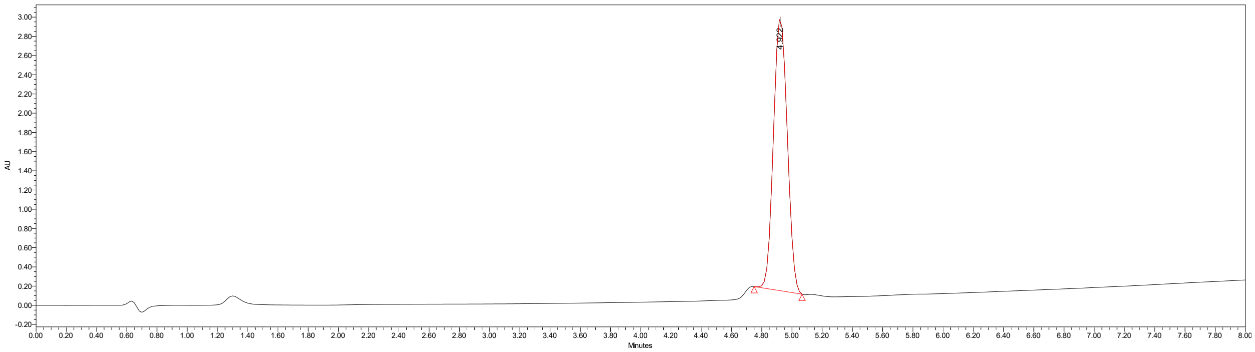

ESI-MS:

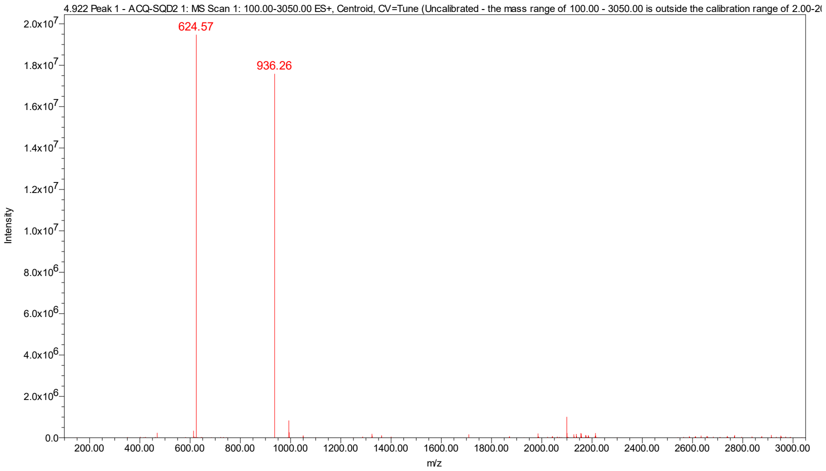

Calculated $[M+3H]^{3+}$: 624.22; observed 624.57
Calculated $[M+2H]^{2+}$: 935.84; observed 936.26

MALDI-TOF-MS:

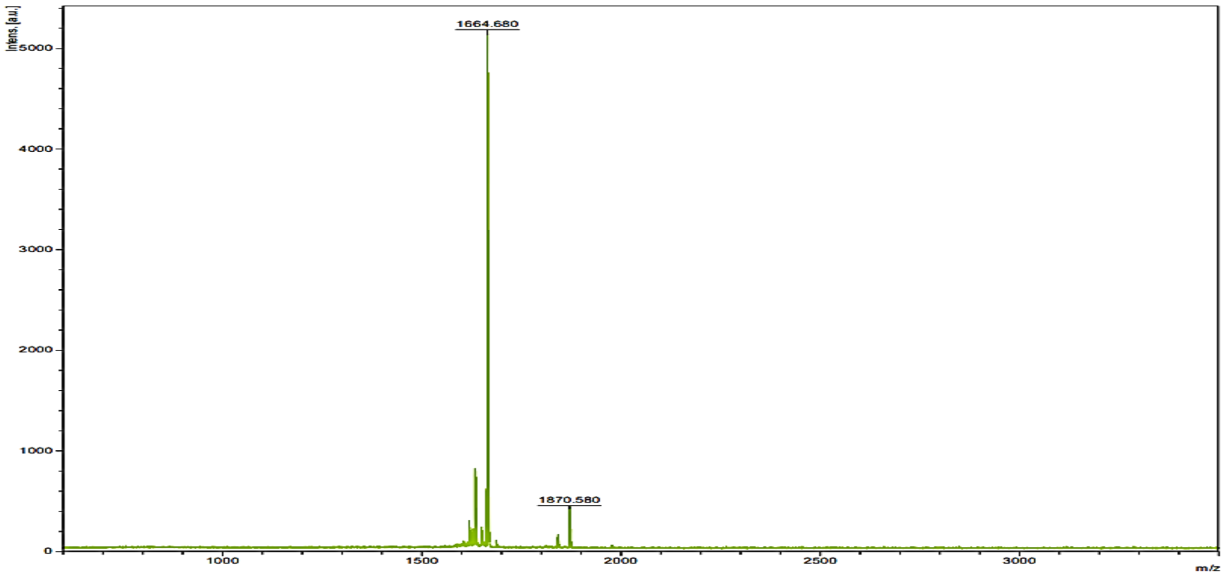

Calculated $[M+H]^+$ (Bismuth bound): 1870.67; observed 1870.58
Calculated $[M+H]^+$ (Bismuth lost): 1664.71; observed 1664.68

BCP16e (for mouse MTD study)

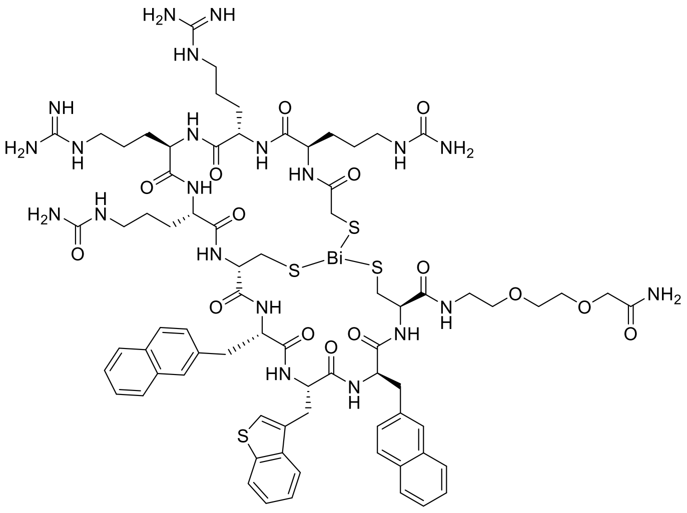

Purity assessment by UPLC (214 nm):

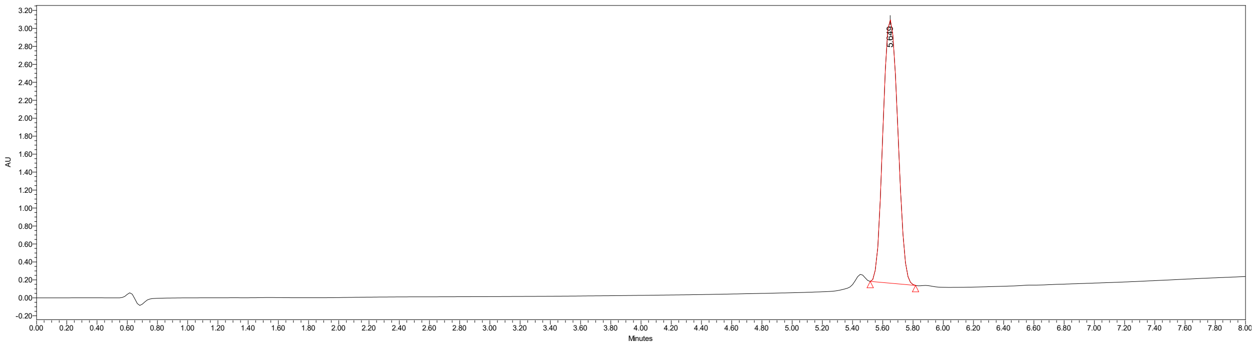

ESI-MS:

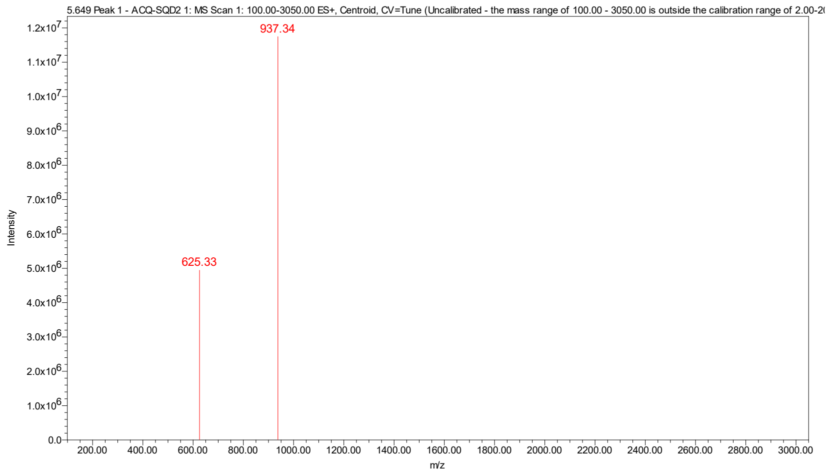

Calculated $[M+3H]^{3+}$: 624.88; observed 625.33
Calculated $[M+2H]^{2+}$: 936.82 observed 937.34

MALDI-TOF-MS:

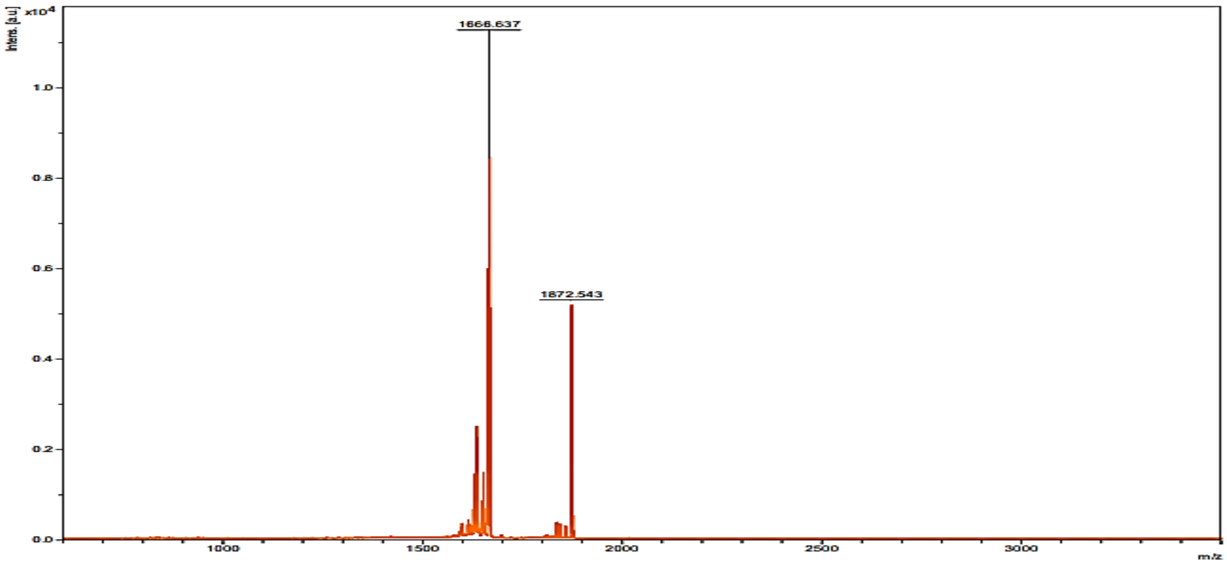

Calculated $[M+H]^+$ (Bismuth bound): 1872.64; observed 1872.543
Calculated $[M+H]^+$ (Bismuth lost): 1666.68; observed 1666.637

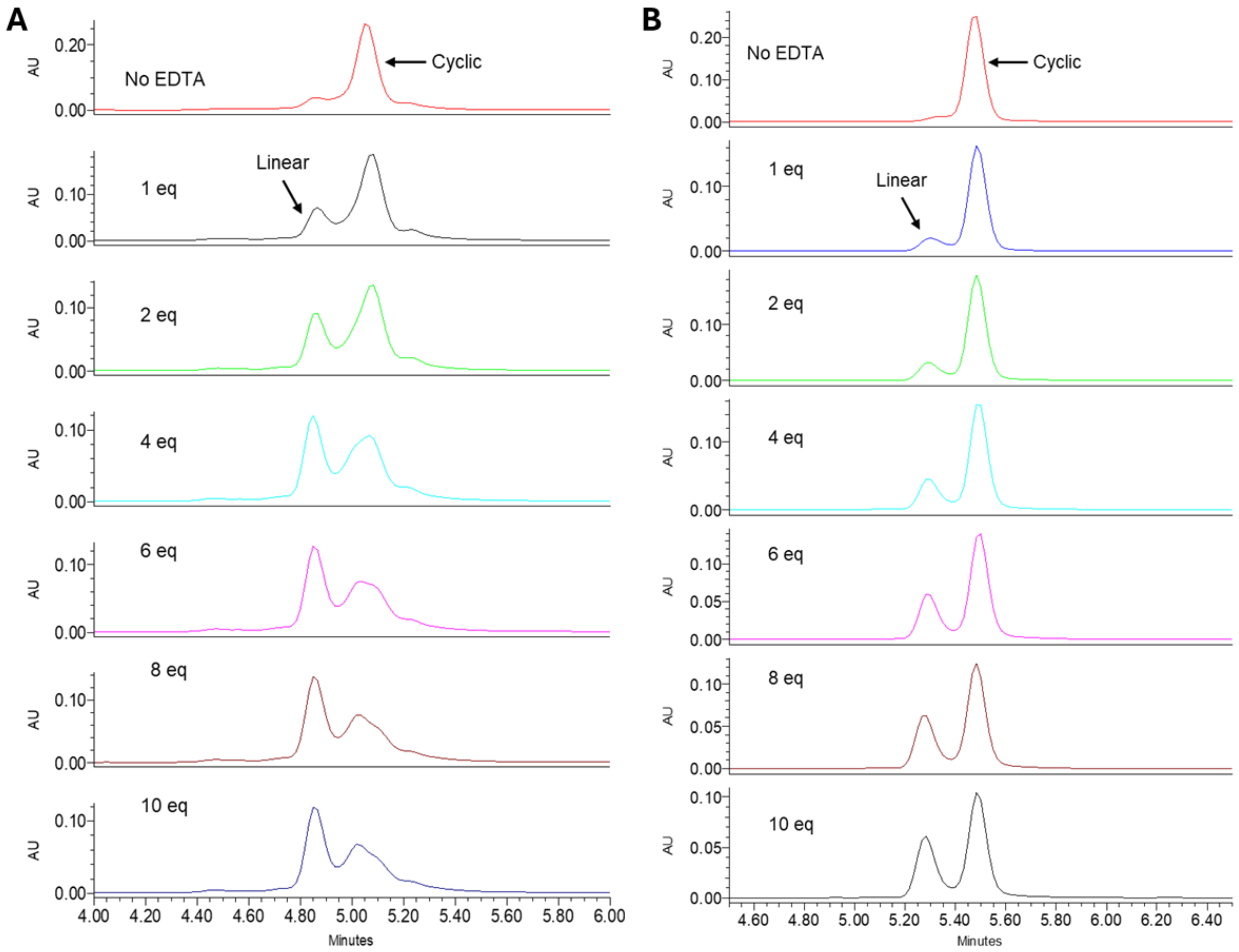

Figure S2. Representative UPLC chromatograms (monitored at 280 nm) showing the dissociation of Bi^{3+} ion from BCP16 (**A**) and BCP16e (**B**) upon incubation with increasing concentrations of EDTA (0–10 equivalents).

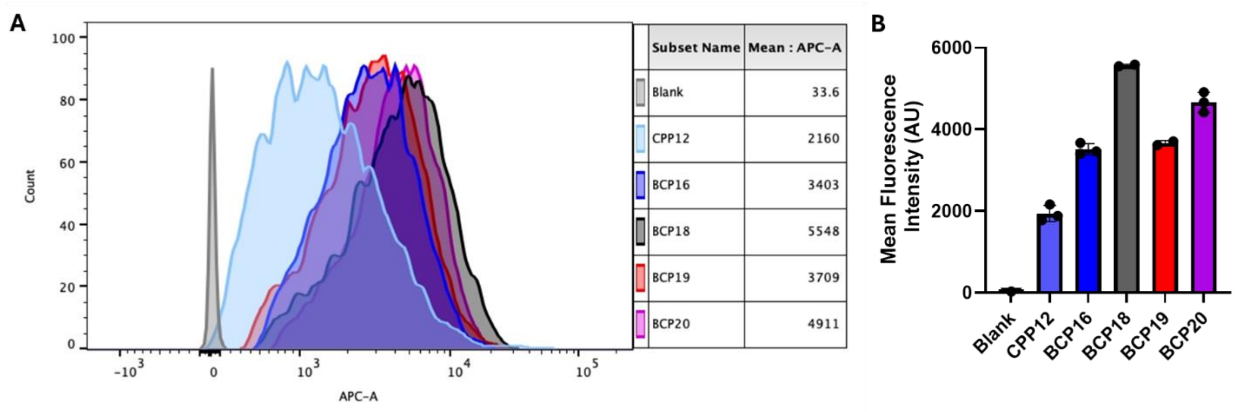

Figure S3: Comparison of the cytosolic entry efficiency of CPP12^{NF}, BCP16^{NF}, BCP18^{NF}, BCP19^{NF}, and BCP20^{NF} in the presence of 1% FBS. (**A**) Flow cytometry analysis of HeLa cells after treatment for 2 h with 2 μM NF-labelled peptides in the presence of 1% FBS (APC channel). Blank represents untreated HeLa cells (no peptide). (**B**) Comparison of the mean fluorescence intensity of HeLa cells from (**A**). Data shown represents the mean ± SD of three independent sets of experiments (n = 3).

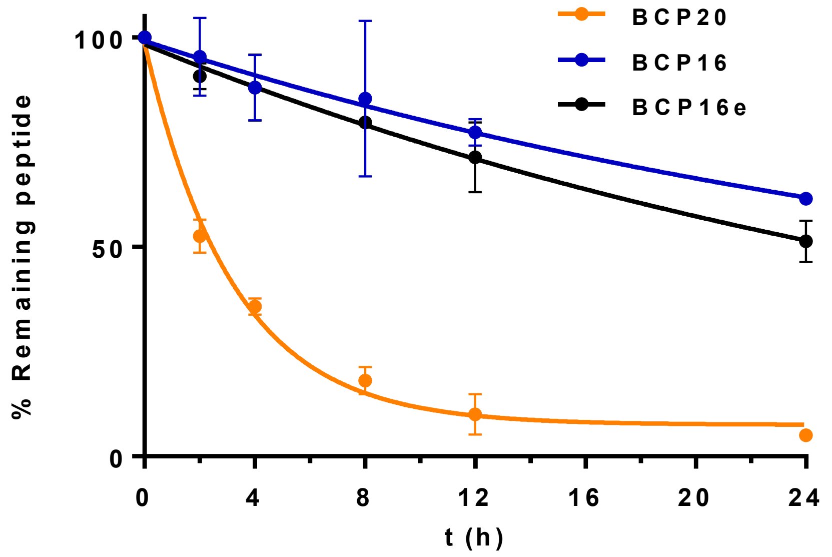

Figure S4: Serum stability of BCP16, BCP16e, and BCP20 ($t_{1/2}$ = 27, 25, and 2.2 h, respectively). Data shown represents the mean ± SD of three independent sets of experiments (n = 3).

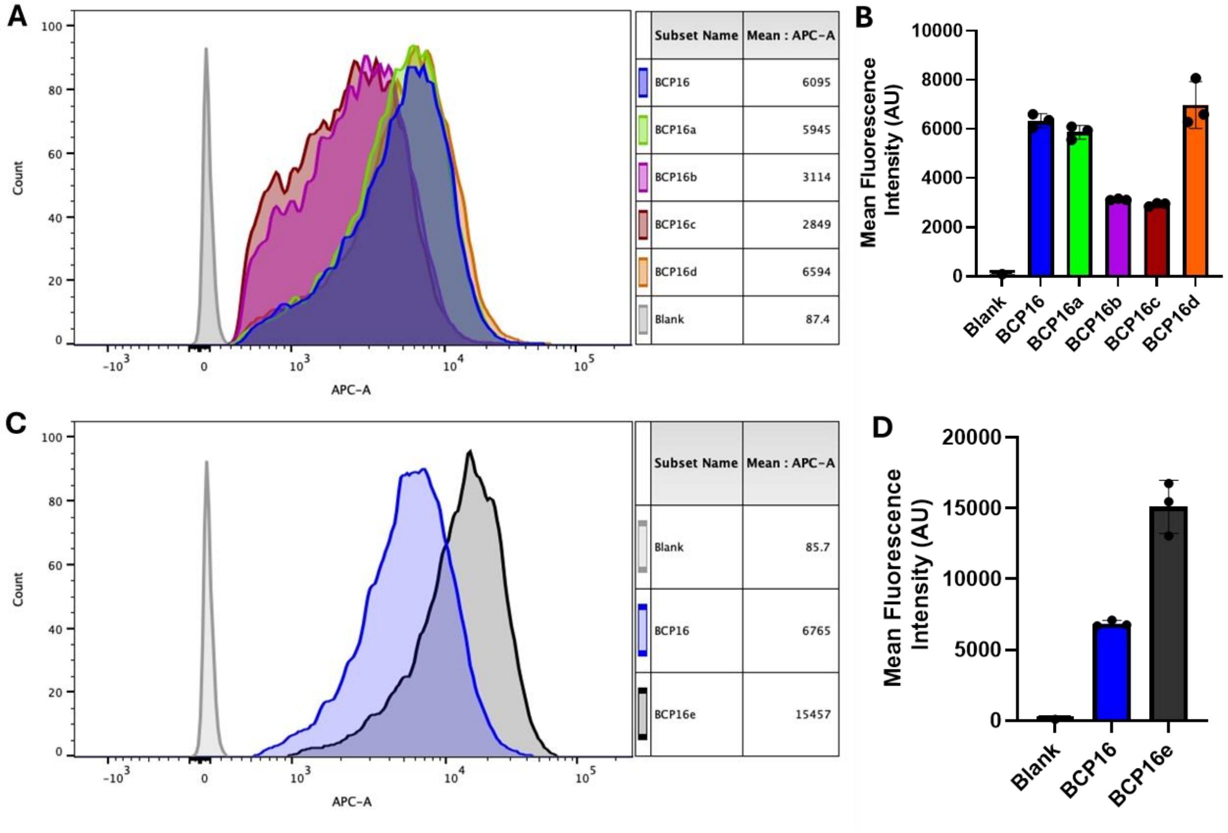

Figure S5. Comparison of the cytosolic entry efficiency of BCP16 and BCP16a-e in the presence of 1% FBS. (**A, C**) Flow cytometry analysis of HeLa cells after treatment for 2 h with 2 μM NF-labelled peptides in the presence of 1% FBS (APC channel). Blank represents untreated HeLa cells (no peptide). (**B, D**) Comparison of the mean fluorescence intensity of HeLa cells from (**A**) and (**C**). Data shown represents the mean ± SD of three independent sets of experiments (n = 3).

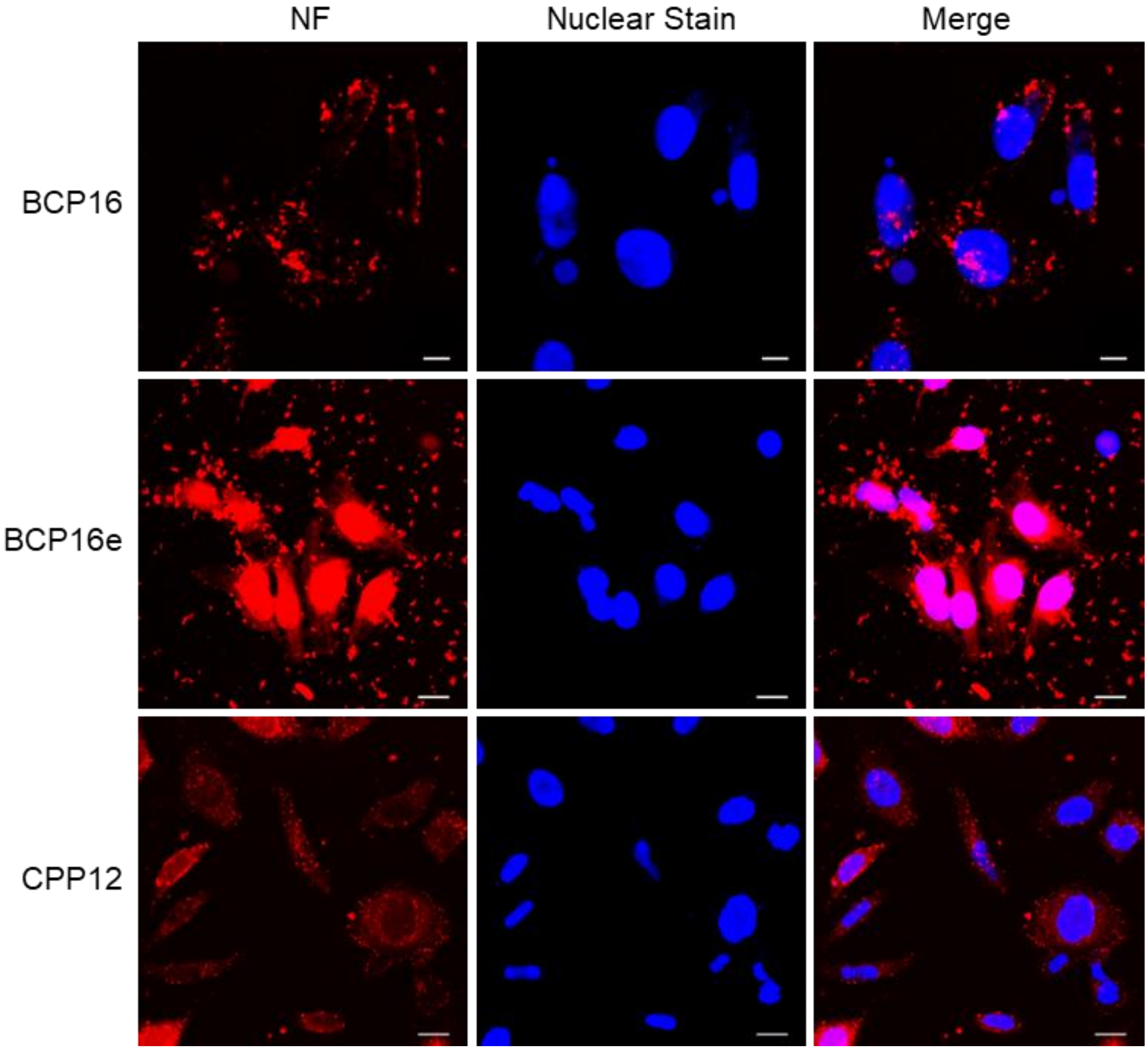

Figure S6. Live-cell confocal microscopic images of HeLa cells after treatment with 2 μM naphthofluorescein (NF)-labeled BCP16, BCP16e, or CPP12 for 2 h in DMEM supplemented with 1% FBS. Scale bars, 20 μm.

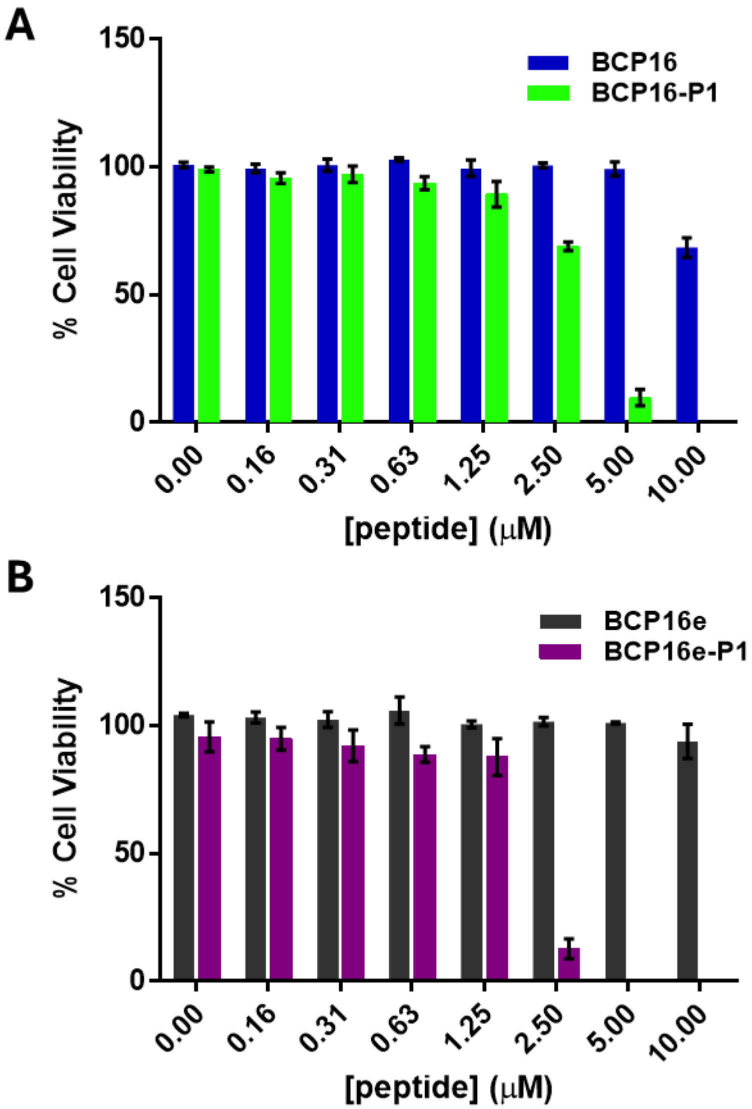

Figure S7. Effect of peptides on HepG2 cell viability as measured by the CellTiter-Glo assay in the presence of 10% FBS. (**A**) HepG2 cells after treatment with indicated concentrations of BCP16 and BCP16-P1. (**B**) HepG2 cells after treatment with BCP16e and BCP16e-P1. Values reported are relative to that of vehicle (no peptide, 100%) and represent the mean ± standard deviation of three biological replicates (n = 3).
